# Female Sex and Mortality in Patients With Gram-Negative Bacteremia: A Systematic Review and Meta-Analysis

**DOI:** 10.1001/jamanetworkopen.2025.43552

**Published:** 2025-11-13

**Authors:** Priscilla La, Rachel Korn, Phillip B. Cox, Divyam Goel, Jean Francois Jabbour, Annette C. Westgeest, Stacey A. Maskarinec, Roberta Monardo, Joshua Parsons, Felicia Ruffin, Merel Lambregts, Yazhong Tao, Garret Smith, Samantha Keller, Mahi Patel, Sarah Cantrell, Vance G. Fowler, Joshua T. Thaden

**Affiliations:** 1 Division of Infectious Diseases, Duke University School of Medicine, Durham, North Carolina; 2 Duke University School of Medicine, Durham, North Carolina; 3 Leiden University Center of Infectious Diseases (LUCID), Leiden University Medical Center, Leiden, the Netherlands; 4 Department of Medicine, San Raffaele Vita-Salute University, Milan, Italy; 5 Duke University Medical Center Library and Archives, Duke University School of Medicine, Durham, North Carolina

## Abstract

**Importance:**

Female sex has been identified as a risk factor for mortality in *Staphylococcus aureus* bloodstream infection (SA-BSI). It is unknown whether this association extends to bloodstream infections with other bacterial species.

**Objective:**

To investigate whether female sex is associated with increased mortality risk among patients with gram-negative bloodstream infection (GN-BSI).

**Data Sources:**

MEDLINE, Embase, and Web of Science were searched from inception to January 8, 2025.

**Study Selection:**

Study inclusion criteria were randomized or observational studies assessing adults with GN-BSI that included at least 100 patients and reported mortality at or before 90 days following GN-BSI, with mortality stratified by sex and, when applicable, by gram-negative bacterial species. Studies with polymicrobial GN-BSI were excluded. For inclusion in the primary analysis, studies must have stratified or statistically adjusted for confounding variables between female and male patients with GN-BSI. A secondary analysis included studies that reported sex-stratified unadjusted mortality.

**Data Extraction and Synthesis:**

One reviewer conducted extraction and quality assessment, which was verified by a second reviewer. Risk of bias and quality were assessed with the Newcastle-Ottawa Quality Assessment Scale. Mortality data were combined as odds ratios (ORs). The study followed the Meta-Analysis of Observational Studies in Epidemiology (MOOSE) reporting guideline.

**Main Outcome and Measures:**

Mortality at or before 90 days following GN-BSI, stratified by sex.

**Results:**

From 9752 studies retrieved, 25 (16 350 patients; 4017 female [25%], 12 333 male [75%]) were included in the primary analysis. Female patients with GN-BSI did not have increased risk of mortality relative to male patients (pooled OR, 0.98 [95% CI, 0.81-1.17]). No publication bias was identified. Subset analyses based on medical comorbidities, timing of mortality end point, bacterial species group, antibiotic resistance phenotype, and publication date did not reveal a set of patients with differences in sex-stratified mortality. A total of 321 studies (147 810 patients) that reported unadjusted mortality were included in a secondary analysis. In this analysis, female sex was associated with decreased risk of mortality (pooled OR, 0.90 [95% CI, 0.86-0.94]).

**Conclusions and Relevance:**

In this systematic review and meta-analysis, female patients with GN-BSI were not at higher risk than male patients of mortality after statistical adjustment. GN-BSI and SA-BSI thus differ in sex-specific mortality outcomes, highlighting the need for further research into the immunological, pathophysiological, and clinical management factors that may be associated with sex disparities in SA-BSI but not in GN-BSI.

## Key Points

**Question:**

Are female patients with gram-negative bloodstream infection at increased risk of death relative to male patients?

**Findings:**

In this systematic review and meta-analysis that included 16 350 patients from 25 studies in the primary analysis, female sex was not associated with increased mortality. No patient subgroups with sex-specific differences in mortality were identified in the adjusted analyses.

**Meaning:**

Findings suggest that in contrast to female patients with *Staphylococcus aureus* bloodstream infection, those with gram-negative bloodstream infection are not at increased risk of death relative to male patients.

## Teaser

This systematic review and meta-analysis explores whether there is an association between biological sex and mortality among patients with gram-negative bloodstream infection.

## Introduction

Biological sex is increasingly recognized as an important determinant of infectious disease outcomes.^1^ In patients with bacteremia, sex-based differences have been observed in disease incidence, treatment, and mortality rates.^1,2^ However, sex remains overlooked as a variable of primary interest in infectious disease research. Understanding the impact of sex is essential for improving patient care in both male and female patients as well as for understanding disease pathogenesis. In a recent large meta-analysis with over 90 000 patients with *Staphylococcus aureus* bloodstream infection (SA-BSI), members of our team observed that female patients experience 18% higher mortality risk than male patients.^3^ However, it is unclear whether this association is specific to SA-BSI or also applies to gram-negative bloodstream infection (GN-BSI). Therefore, the aim of this systematic review and meta-analysis was to evaluate the association between female sex and mortality among patients with GN-BSI.

## Methods

The key question of this systematic review and meta-analysis was: is female sex associated with increased mortality risk in patients with GN-BSI? Our hypothesis was that female sex would be associated with increased mortality risk in patients with GN-BSI. The study protocol is registered on Prospero (CRD420250652146) (eAppendix 1 in Supplement 1). We followed the Meta-Analysis of Observational Studies in Epidemiology (MOOSE) reporting guideline,^4^ as the included studies involved observational data. This study used only summary data from published studies; thus, no institutional review board review or informed consent was required.

### Search Strategy

We conducted a literature search of MEDLINE via Ovid, Embase via Elsevier, and Web of Science’s Science Citation Index Expanded (1900 to the present) and Emerging Sources Citation Index (2019 to the present) via Clarivate from inception to January 8, 2025, using a combination of key words to capture gram-negative bacteria, bacteremia, and sex. An experienced medical librarian (S.C.) devised, developed, and executed the search with input from the entire team. The search was peer reviewed by a second medical librarian according to a modified Peer Review of Electronic Search Strategies checklist.^5^ Studies published in languages other than English were excluded. Authors of relevant papers were contacted directly as needed for clarity or when full text could not be readily obtained. The search strategies for all included databases are given in eAppendix 2 in Supplement 1. References from key manuscripts were also screened. All results were compiled in EndNote and imported into Covidence, a web-based data synthesis software program,^6^ for deduplication and screening.

### Study Selection, Data Extraction, and Quality Assessment

We included studies that (1) were a randomized or observational study evaluating outcomes in adults with GN-BSI, (2) included at least 100 patients of either a single bacterial species or a mixed population of gram-negative bacterial species, (3) performed stratification by gram-negative bacterial species when mixed bacterial populations were reported (eg, both gram-positive and gram-negative bacteria), (4) reported mortality at or before 90 days following GN-BSI, and (5) reported mortality stratified by sex. Exclusion criteria were polymicrobial bloodstream infection, pediatric patients, studies published in a language other than English, and studies using the same or largely overlapping cohort as another study included in this review. In this latter scenario, the study with the largest cohort was included. Titles and abstracts of articles identified through our primary search were screened independently by 2 reviewers (all authors except S.C.). Conflicts at this stage were resolved by a third reviewer (P.L. or J.T.T.). Articles marked for full text review underwent full-text screening by 2 independent reviewers (all authors except S.C.). Discrepancies at this stage were also adjudicated by a third reviewer (P.L. or J.T.T.). Data extraction and quality assessment were performed by 1 reviewer and verified by a second reviewer. Extracted variables included lead author, journal, year of publication, start and end years of study, country, aim of study, study design, number of hospitals, number of patients, population description, and mortality data. Unadjusted mortality stratified by sex was extracted, as well as adjusted mortality when reported, along with the statistical model and the covariates for which mortality was adjusted. If a study described mortality for 2 subgroups (eg, for antibiotic-resistant and antibiotic-susceptible bacteremia separately), both were included. For inclusion in the primary analysis, studies must have stratified or statistically adjusted for confounding variables between female and male patients with GN-BSI. We hypothesized that any sex-specific mortality difference would be mediated by changes in acute severity of illness. Therefore, studies that stratified or statistically adjusted for acute severity of illness with either a validated scoring instrument (eg, Pitt bacteremia score, APACHE-II acute physiology score) or a proxy for increased severity, such as presence of septic shock, were not included in the primary analysis.^7,8^ Studies that adjusted for acute severity of illness were included in an exploratory analysis. A secondary analysis included studies that reported sex-stratified unadjusted mortality. Risk of bias and quality were assessed with the Newcastle-Ottawa Quality Assessment Scale^9^ (eAppendix 3 in Supplement 1) because only observational studies were identified.

### Statistical Analysis

Mortality data were assessed using odds ratios (ORs). If ORs were not reported in a study, we calculated them from raw mortality by sex. If raw data were not available, ORs were calculated from the provided risk ratio or hazard ratio values based on previously published methods.^10,11^ ORs were combined using inverse variance with random effects models. We used the Knapp and Hartung method to adjust the standard errors of the estimated coefficients.^12,13^ The robustness of findings was assessed through influence and sensitivity analyses as detailed in the *Mortality by Sex* section of the Results. We evaluated statistical heterogeneity with the *I*^2^, τ^2^, Cochran *Q* test, and prediction interval statistics. Some meta-analysis subgroups contained a small number of studies (n < 5), and the *I*^2^ statistic may be inaccurate when a low number of studies are included in the analysis.^14^ Given this limitation, examination of heterogeneity primarily relied on the Cochran *Q* test and prediction interval, which was calculated from τ^2^. The prediction interval presents the heterogeneity on the same scale as the original outcomes and contains highly probable values for the true treatment effects in future settings.^15^ To explore potential sources of heterogeneity, we performed meta-analyses on subsets of studies to determine whether variation in factors such as patient variables (eg, age, source of GN-BSI, and medical comorbidities), mortality time point (eg, in-hospital vs 28- to 30-day mortality), bacterial group (eg, Enterobacterales, non–lactose fermenters, and mixed populations), antibiotic resistance phenotypes (eg, carbapenem resistance), and study publication date could be contributing. Statistical analyses were performed with RStudio 2024.12.1 (Posit Software). Publication bias was assessed using funnel plots with the Egger test^16^ when at least 10 studies were included in the analysis. We used the Evidence-Based Practice Center model from the US Agency for Healthcare Research and Quality to grade overall strength of evidence.^17^ A full description of this approach is detailed in eAppendix 4 in Supplement 1. A 2-sided value of *P* < .05 was considered statistically significant.

## Results

### Summary of Included Studies

**Figure 1.**
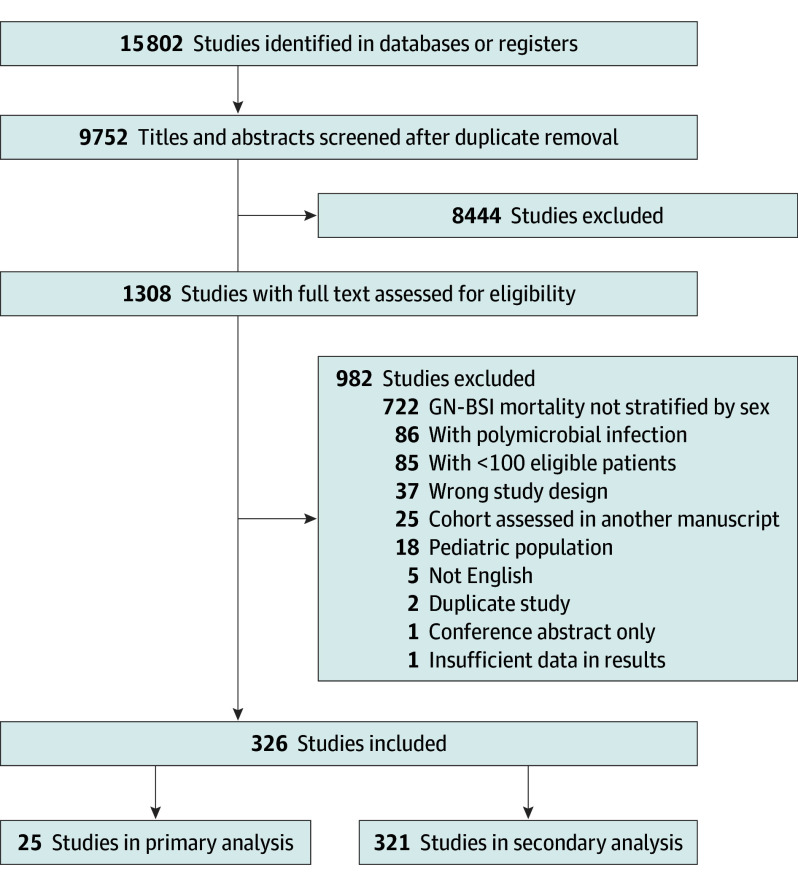
Preferred Reporting Items for Systematic Reviews and Meta-Analyses (PRISMA) Flowchart GN-BSI indicates gram-negative bloodstream infection.

Titles and abstracts of 9752 studies were screened for eligibility, and 8444 studies were excluded for irrelevance. Full texts of 1308 studies were assessed, and 982 studies were excluded. In total, 25 studies (n = 16 350 patients) with 4017 female patients (25%) and 12 333 male patients (75%) were included in the primary analysis.^18,19,20,21,22,23,24,25,26,27,28,29,30,31,32,33,34,35,36,37,38,39,40,41,42^ A total of 321 studies (n = 147 810 patients) were included in a secondary analysis that examined studies reporting unadjusted mortality stratified by sex (Figure 1).^19,21,22,23,24,25,26,27,28,29,30,33,34,35,36,37,38,39,40,41,42,43,44,45,46,47,48,49,50,51,52,53,54,55,56,57,58,59,60,61,62,63,64,65,66,67,68,69,70,71,72,73,74,75,76,77,78,79,80,81,82,83,84,85,86,87,88,89,90,91,92,93,94,95,96,97,98,99,100,101,102,103,104,105,106,107,108,109,110,111,112,113,114,115,116,117,118,119,120,121,122,123,124,125,126,127,128,129,130,131,132,133,134,135,136,137,138,139,140,141,142,143,144,145,146,147,148,149,150,151,152,153,154,155,156,157,158,159,160,161,162,163,164,165,166,167,168,169,170,171,172,173,174,175,176,177,178,179,180,181,182,183,184,185,186,187,188,189,190,191,192,193,194,195,196,197,198,199,200,201,202,203,204,205,206,207,208,209,210,211,212,213,214,215,216,217,218,219,220,221,222,223,224,225,226,227,228,229,230,231,232,233,234,235,236,237,238,239,240,241,242,243,244,245,246,247,248,249,250,251,252,253,254,255,256,257,258,259,260,261,262,263,264,265,266,267,268,269,270,271,272,273,274,275,276,277,278,279,280,281,282,283,284,285,286,287,288,289,290,291,292,293,294,295,296,297,298,299,300,301,302,303,304,305,306,307,308,309,310,311,312,313,314,315,316,317,318,319,320,321,322,323,324,325,326,327,328,329,330,331,332,333,334,335,336,337,338,339,340,341,342^ Characteristics of studies included in the primary analysis are shown in the Table. All studies included in the primary analysis were observational, and all but a single study, a randomized clinical trial, in the secondary analysis were observational. Mortality was most frequently assessed at 28 to 30 days (20 of 25 studies [80%]). The majority of studies were conducted in Europe (12 [48%]), Asia (7 [28%]), and North America (5 [20%]). Most studies focused on species within the Enterobacterales order (17 [68%]). Most studies did not specify a particular antibiotic resistance phenotype (19 [76%]). None of the studies were primarily aimed at examining sex differences in patients with GN-BSI. All studies were rated as having low risk of bias (eAppendix 5 in Supplement 1). Characteristics of studies included in the secondary analysis of unadjusted mortality are shown in eAppendix 6 in Supplement 1.

**Table.** Summary of Studies Included in the Primary Analysis

Variable	No. (%)	
	Studies (n = 25)	Patients (n = 16 350)
Publication year		
Before 2000	1 (4)^38^	815 (5)
2000-2009	1 (4)^20^	958 (6)
2010-2019	5 (20)^19,22,34,35,39^	2215 (14)
2020-2025	18 (72)^18,21,23,24,25,26,27,28,29,30,31,32,33,36,37,40,41,42^	12 362 (76)
Study design		
Cohort	24 (96)^18,19,20,21,22,23,24,25,26,27,28,29,30,31,32,33,34,35,36,37,38,39,41,42^	16 150 (99)
Retrospective quasi-experimental	1 (4)^40^	200 (1)
Continent		
Europe	12 (48)^21,22,26,27,28,29,32,36,37,38,39,41^	4715 (29)
Asia	7 (28)^18,23,24,33,34,35,42^	1838 (11)
North America	5 (20)^19,20,30,31,40^	9669 (59)
Australia	1 (4)^25^	128 (1)
No. of hospitals included		
1	13 (52)^18,21,23,24,25,26,31,32,33,34,35,39,42^	3268 (20)
2-10	7 (28)^19,20,22,27,28,38,40^	3362 (21)
>10	5 (20)^29,30,36,37,41^	9720 (59)
No. of patients included		
100-999	24 (96)^18,19,20,21,22,23,24,25,26,27,28,29,31,32,33,34,35,36,37,38,39,40,41,42^	8311 (51)
1000-9999	1 (4)^30^	8039 (49)
Bacterial species groups		
Enterobacterales only	17 (68)^19,20,22,23,24,26,28,29,32,33,35,36,37,38,40,42^	7042 (43)
Non–lactose fermenters only	3 (12)^25,27,30^	8324 (51)
All gram-negative bacteria	5 (20)^18,21,31,34,39^	984 (6)
Antibiotic resistance phenotype		
Not specified	19 (76)^18,19,20,21,22,23,25,27,29,30,31,32,34,35,38,39,40,41,42^	15 233 (93)
Carbapenem resistant	5 (20)^26,28,33,36,37^	1015 (6)
Extended-spectrum beta-lactamase producer	1 (4)^24^	102 (1)
Population		
All hospitalized patients	17 (68)^18,19,20,22,25,29,30,32,33,34,35,36,37,38,40,41,42^	14 837 (91)
Patients with hematological malignancy	2 (8)^31,39^	454 (3)
Intensive care unit setting	2 (8)^27,28^	308 (2)
Health care–associated or hospital onset	1 (4)^26^	435 (3)
History of transplantation	1 (4)^21^	113 (1)
Older adult patients	1 (4)^24^	101 (1)
Patients with liver cirrhosis	1 (4)^23^	102 (1)
Mortality outcome measure^a^		
7 d	2 (8)^34,37^	326 (2)
28-30 d	20 (80)^19,21,22,23,24,25,26,27,28,29,30,31,33,35,36,38,39,40,41,42^	14 521 (89)
90 d	2 (8)^32,37^	454 (3)
In-hospital	2 (8)^18,20^	1203 (7)

^a^ Studies may report multiple mortality time points.

### Mortality by Sex

**Figure 2.**
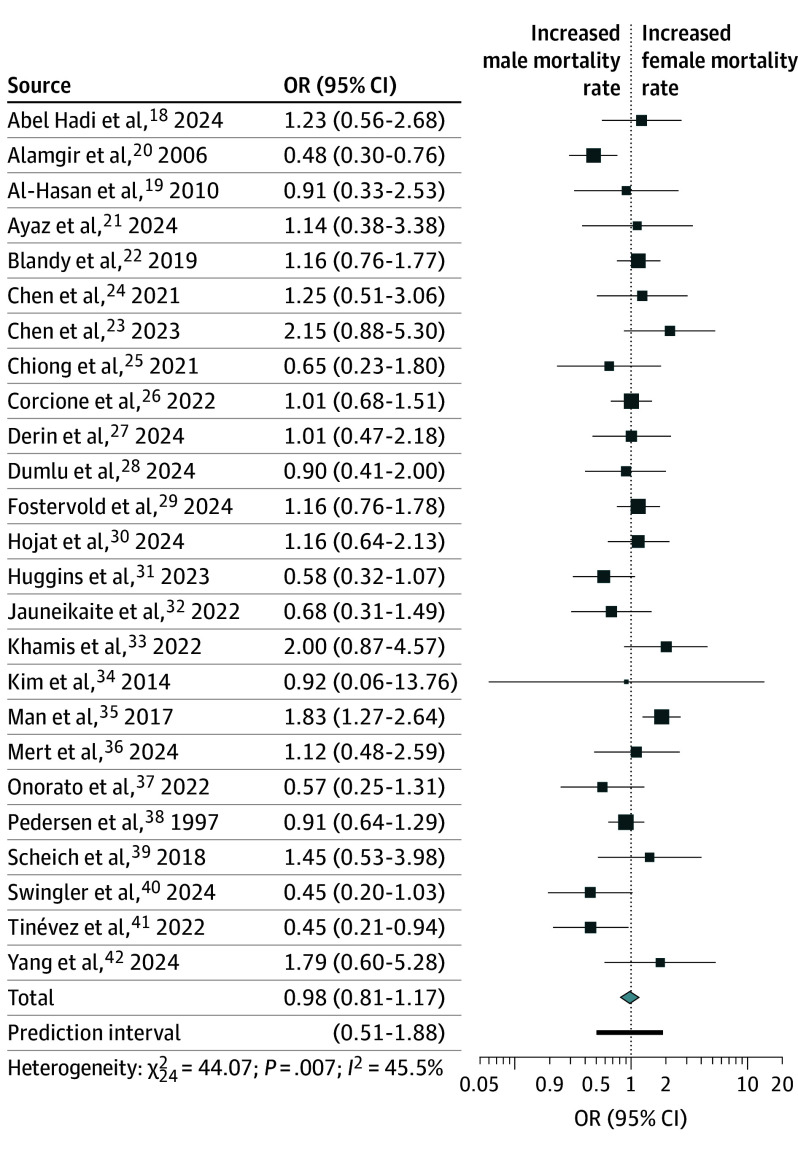
Forest Plot of the Association Between Biological Sex and Mortality Among Patients With Gram-Negative Bloodstream Infection for All Studies Included in the Primary Analysis OR, odds ratio.

Mortality data from 25 studies included in the primary analysis^18,19,20,21,22,23,24,25,26,27,28,29,30,31,32,33,34,35,36,37,38,39,40,41,42^ showed no difference in mortality risk for female compared with male patients (pooled OR, 0.98 [95% CI, 0.81-1.17]) (Figure 2). An influence analysis demonstrated that omission of any single study did not significantly alter results (eAppendix 7 in Supplement 1). No significant publication bias was detected through either a funnel plot analysis (eAppendix 8 in Supplement 1) or the Egger test (*P* = .58). Moderate heterogeneity was observed in this analysis (*I*^2^ = 45.5%; τ^2^ = 0.092; *P* = .008; prediction interval, 0.51-1.88). To determine if these findings may have been associated with our criteria for inclusion in the primary analysis, we performed an exploratory meta-analysis with all studies that reported adjusted sex-stratified mortality among patients with GN-BSI. This meta-analysis included studies that performed an analysis that adjusted for acute severity of illness. In this exploratory meta-analysis of 59 studies (n = 38 660 patients),^18,19,20,21,22,34,36,37,38,39,40,41,42,51,53,54,58,63,68,73,77,78,87,111,116,142,152,157,179,180,182,204,208,211,217,222,245,253,258,276,277,311,316,336,337,339,343^ no association between biological sex and mortality was identified (pooled OR, 0.94 [95% CI, 0.83-1.06]) (eAppendix 9 in Supplement 1).

To identify potential subsets of patients with sex-specific mortality differences and to better understand the observed heterogeneity in the overall meta-analysis, we performed multiple subset analyses. Subset analyses focused on patient clinical factors included in the adjusted models (eAppendix 10 in Supplement 1), mortality timing end points (eAppendix 11 in Supplement 1), bacterial species groups (eAppendix 12 in Supplement 1), bacterial antibiotic resistance phenotypes (eAppendix 13 in Supplement 1), and publication date (eAppendix 14 in Supplement 1) similarly revealed no association between biological sex and mortality among patients with GN-BSI. Examination of these subsets revealed several potential sources of heterogeneity. First, variability in the examined antibiotic resistance phenotype was associated with heterogeneity, as studies focused only on carbapenem-resistant bacteria (eAppendix 13B in Supplement 1) exhibited a nonsignificant Cochran *Q* test result (*P* = .33; *I*^2^ = 12.7%, τ^2^<0.001) and a prediction interval similar to the meta-analysis confidence interval. This finding contrasts with studies that did not specify a particular antibiotic resistance phenotype (*P* = .003; *I*^2^ = 54.1%, τ^2^ = 0.212). Second, variation in bacterial species groups may be a source of heterogeneity, as studies examining non–lactose-fermenting bacteria had a nonsignificant Cochran *Q* test result (*P* = .63; *I*^2^ = 0.0%, τ^2^ = 0) and a prediction interval similar to the meta-analysis confidence interval (eAppendix 12B in Supplement 1). This finding contrasts with studies that examined only Enterobacterales (Cochrane *Q* test, *P* = .001; *I*^2^ = 59.2%, τ^2^ = 0.129) (eAppendix 12A in Supplement 1). Third, statistical adjustment for clinical variables beyond age may be a source of heterogeneity, as studies that also adjusted for source of GN-BSI (eAppendix 10A in Supplement 1; Cochran Q *P* = .27; *I*^2^ = 20.1%, τ^2^ = 0.029), medical comorbidities with a weighted index measure (eAppendix 10B in Supplement 1; Cochran Q *P* = .27; *I*^2^ = 20.1%, τ^2^ = 0.029), or both (eAppendix 10C in Supplement 1; Cochran *Q P = *.87; *I*^2^ = 0.0%, τ^2^ = 0) exhibited nonsignificant Cochran *Q P* values relative to the primary analysis (Figure 2; Cochran Q *P* = .007 *I*^2^ = 45.5%).

In the secondary meta-analysis of studies reporting unadjusted sex-stratified mortality data, female sex was associated with decreased mortality risk compared with male sex among patients with GN-BSI (pooled OR, 0.90 [95% CI, 0.86-0.94]) (eAppendix 15 in Supplement 1). Moderate heterogeneity was observed (*I*^2^ = 49.3%, τ^2^ = 0.065, *P* < .001). Publication bias was indicated by the Egger regression test (*P* = .003). The funnel plot is shown in eAppendix 16 in Supplement 1.

### Evaluation of Evidence

Given that this systematic review contained observational studies that accounted for confounding through statistical adjustment (ie, the adjusted analysis), the baseline strength of evidence was moderate. The mortality effect estimate was downrated due to a serious risk of bias because studies without a sex difference in a univariable analysis would likely not have included this variable in a multivariable analysis. We did not have serious concerns about inconsistency, indirectness, imprecision, or publication bias. An additional limitation included that sex difference was not the primary outcome of interest in any of the studies that were included. Therefore, the overall strength of evidence for the association of female sex with increased mortality risk among patients with GN-BSI was low.

## Discussion

This systematic review and meta-analysis examined the association between biological sex and mortality among patients with GN-BSI. We found that after adjusting for key clinical confounders, there was no significant difference in mortality between female and male patients. In contrast, our secondary analysis of studies reporting unadjusted mortality showed a significantly lower risk of mortality among female patients compared with male patients. These findings suggest that any raw mortality difference between male and female patients with GN-BSI is associated with variation in the covariates included in the adjusted models and not associated with biological sex. All studies in the primary analysis adjusted for age. Adjustments for medical comorbidities and source of GN-BSI were also common. These findings also highlight the importance of statistical adjustment for important confounders when examining observational data.

Members of our team previously performed a systematic review and meta-analysis demonstrating that female patients with SA-BSI are at increased risk of mortality relative to male patients.^3^ Another study involving a large nationwide cohort of patients with SA-BSI also showed that female patients are at increased risk of mortality and further demonstrated that this increased mortality risk may be attributable to the infection.^344^ However, it is unclear whether this sex-specific difference in bloodstream infection mortality is unique to *S aureus* or extends to other bacterial species; therefore, we evaluated the association between biological sex and mortality among patients with GN-BSI in this systematic review and meta-analysis. GN-BSI mortality varies greatly by factors such as age, source of the infection, and medical comorbidities.^343,345,346^ Therefore, our primary analysis focused on studies that either stratified or statistically adjusted for confounding factors between female and male patients that may be associated with mortality.

The challenges of considering GN-BSI as a single entity were demonstrated through our examination of heterogeneity. The primary meta-analysis, which included all GN-BSI studies meeting the inclusion and exclusion criteria, exhibited moderate to large heterogeneity. We explored the sources of this heterogeneity through subset analyses and revealed multiple potential causes. Factors such as the bacterial species groups being addressed, antibiotic resistance phenotype, and choice of covariates to include in the adjusted models all influenced the observed heterogeneity. Relative to the overall cohort of studies included in the primary analysis, studies focused on non–lactose-fermenting gram-negative bacteremia only or carbapenem-resistant organisms only demonstrated lower heterogeneity. Studies that statistically adjusted for both source of GN-BSI and medical comorbidities through a weighted index measure (eg, Charlson Comorbidity Index) similarly demonstrated decreased heterogeneity. In addition, GN-BSI itself, as well as the statistical approaches to address the epidemiology of GN-BSI, are heterogeneous. However, the lack of association between biological sex and GN-BSI mortality in either the primary meta-analysis or any of the subgroup meta-analyses suggests that this finding is relatively robust.

This study expands our limited knowledge of sex-specific outcomes in bloodstream infections and highlights the complex nature of the interaction between sex and bacterial bloodstream infections. Our current findings in GN-BSI, in contrast with those from the prior study in SA-BSI by members of our team,^3^ suggest that sex-based mortality differences in bloodstream infections are specific to *S aureus*. The reasons for this divergence are unclear but are likely multifaceted. Clinical outcomes may be impacted by a broad range of factors, including demographics and medical comorbidities, clinical presentation (eg, source of bloodstream infection, time to diagnosis), treatment (eg, timing and appropriateness of effective antibiotic therapy and diagnostic testing), and immunological response to infection.^51,52^ Given the unique clinical profile of SA-BSI relative to bacteremia caused by other microorganisms, it is plausible that these factors play a less important role in bloodstream infections other than SA-BSI. For example, female patients with SA-BSI have been shown to differ from male patients with respect to medical comorbidities, have shorter duration of therapy, and less often are evaluated with transesophageal echocardiography.^2^ Female patients with SA-BSI may also differ from male patients with respect to the immunological response to infection, although this has largely been unexplored.

The present study also highlights the historical neglect of the association of sex and gender with patient outcomes in infectious disease research.^347,348^ Given the current limited evidence suggesting that female patients with sepsis and with SA-BSI are often subjected to suboptimal management strategies and experience worse outcomes,^43,349,350^ it is surprising that no study in our search focused on sex differences in GN-BSI. To promote more equitable, evidence-based care, research on this issue is necessary.

### Limitations

This study has several limitations. First, sex difference was not the primary outcome of interest in any of the included studies. Therefore, many studies did not include mortality data adjusted for sex, and inclusion of these data could have impacted the results. Due primarily to these issues, the overall strength of evidence for the lack of association between female sex and mortality was low. Second, heterogeneity was present not only in study methodology but also in the disease itself. Clinical outcomes in GN-BSI may differ greatly based on variability in factors such as source of infection, antibiotic resistance, age, medical comorbidities, and gram-negative bacterial species. While we attempted to address this variability through subgroup analyses, it remains plausible that there are GN-BSI subpopulations with sex-specific differences in mortality rates among patients. However, members of our team have previously shown that the host immunological response to different gram-negative bacterial species (ie, *Escherichia coli* and *Klebsiella pneumoniae*) does not differ in a substantial way.^351^ Third, most studies were conducted at academic medical centers, thus potentially biasing results and limiting the generalizability of our findings.

## Conclusions

In this systematic review and meta-analysis, we found no association between biological sex and mortality among patients with GN-BSI after adjusting for confounding variables between female and male patients. This contrasts with prior findings by members of our team for SA-BSI, which indicated that female patients were at a significantly increased risk for mortality compared with male patients. This highlights the need for further research into the immunological, pathophysiological, and clinical management factors that may be associated with sex disparities in SA-BSI but not in GN-BSI.

## Notes

Supplement 1. **eAppendix 1.** Systematic review protocol **eAppendix 2.** Search strategies **eAppendix 3.** Newcastle-Ottawa Quality Assessment Scale for assessing risk of bias in cohort and case-control studies **eAppendix 4.** Approach for grading the overall strength of evidence **eAppendix 5.** Risk of bias (quality) assessment of included cohort studies using the Newcastle-Ottawa Quality Assessment Scale **eAppendix 6.** Summary of studies included in the secondary (unadjusted mortality) analysis **eAppendix 7**. Influence analysis of studies included in the primary analysis (n = 25) **eAppendix 8.** Funnel plot of studies included in the primary analysis **eAppendix 9.** Exploratory analysis of all manuscripts that reported adjusted sex-stratified mortality data in patients with gram-negative bloodstream infection. **eAppendix 10.** Subset meta-analyses, stratified by clinical variables **eAppendix 11.** Subset meta-analyses, stratified by timing of mortality endpoint **eAppendix 12.** Subset meta-analyses, stratified by bacterial species group **eAppendix 13.** Subset meta-analyses, stratified by bacterial antibiotic resistance phenotype **eAppendix 14.** Subset meta-analyses, stratified by publication date **eAppendix 15.** Secondary analysis of unadjusted sex-stratified mortality in patients with gram-negative bloodstream infection **eAppendix 16.** Funnel plot of studies included in the secondary analysis

Supplement 2.
Data Sharing Statement
