# Supplementary material for: Female Sex and Mortality in Patients With Gram-Negative Bacteremia: A Systematic Review and Meta-Analysis
Source: JAMA Netw Open. 2025 Nov 13;8(11):e2543552. doi: 10.1001/jamanetworkopen.2025.43552 (PMC12616467; doi:10.1001/jamanetworkopen.2025.43552)
Supplement: Supplement 1. — eAppendix 1. Systematic review protocol eAppendix 2. Search strategies eAppendix 3. Newcastle-Ottawa Quality Assessment Scale for assessing risk of bias in cohort and case-control studies eAppendix 4. Approach for grading the overall strength of evidence eAppendix 5. Risk of bias (quality) assessment of included cohort studies using the Newcastle-Ottawa Quality Assessment Scale eAppendix 6. Summary of studies included in the secondary (unadjusted mortality) analysis eAppendix 7. Influence analysis of studies included in the primary analysis (n = 25) eAppendix 8. Funnel plot of studies included in the primary analysis eAppendix 9. Exploratory analysis of all manuscripts that reported adjusted sex-stratified mortality data in patients with gram-negative bloodstream infection. eAppendix 10. Subset meta-analyses, stratified by clinical variables eAppendix 11. Subset meta-analyses, stratified by timing of mortality endpoint eAppendix 12. Subset meta-analyses, stratified by bacterial species group eAppendix 13. Subset meta-analyses, stratified by bacterial antibiotic resistance phenotype eAppendix 14. Subset meta-analyses, stratified by publication date eAppendix 15. Secondary analysis of unadjusted sex-stratified mortality in patients with gram-negative bloodstream infection eAppendix 16. Funnel plot of studies included in the secondary analysis [file jamanetwopen-e2543552-s001.pdf]

Supplementary Online Content

La P, Korn R, Cox PB, et al. Female sex and mortality in patients with gram-negative bacteremia: a systematic review and meta-analysis. *JAMA Netw Open*. 2025;8(11):e2543552. doi:10.1001/jamanetworkopen.2025.43552

eAppendix 1. Systematic review protocol

eAppendix 2. Search strategies

eAppendix 3. Newcastle-Ottawa Quality Assessment Scale for assessing risk of bias in cohort and case-control studies

eAppendix 4. Approach for grading the overall strength of evidence

eAppendix 5. Risk of bias (quality) assessment of included cohort studies using the Newcastle-Ottawa Quality Assessment Scale

eAppendix 6. Summary of studies included in the secondary (unadjusted mortality) analysis

eAppendix 7. Influence analysis of studies included in the primary analysis (n=25)

eAppendix 8. Funnel plot of studies included in the primary analysis

eAppendix 9. Exploratory analysis of all manuscripts that reported adjusted sex-stratified mortality data in patients with gram-negative bloodstream infection.

eAppendix 10. Subset meta-analyses, stratified by clinical variables

eAppendix 11. Subset meta-analyses, stratified by timing of mortality endpoint

eAppendix 12. Subset meta-analyses, stratified by bacterial species group

eAppendix 13. Subset meta-analyses, stratified by bacterial antibiotic resistance phenotype

eAppendix 14. Subset meta-analyses, stratified by publication date

eAppendix 15. Secondary analysis of unadjusted sex-stratified mortality in patients with gram-negative bloodstream infection

eAppendix 16. Funnel plot of studies included in the secondary analysis

eReferences.

© 2025 La P et al. *JAMA Network Open*.

This supplementary material has been provided by the authors to give readers additional information about their work.

© 2025 La P et al. *JAMA Network Open*.

eAppendix 1. Systematic review protocol

Project Title: Association of female sex at birth with mortality in patients with gram-negative bacterial bloodstream infections

Investigators: Priscilla La, BS; Annette C. Westgeest, MD; Joshua T. Thaden, MD, PhD; Vance G. Fowler, Jr., MD, MHS; Samantha Keller; Joshua Parsons, MD, PhD; Felicia Ruffin, PhD; Rachel Korn, BS; Jeffrey Jabbour, MD; Merel Lambregts, MD; Phillip Cox, MS; Garret Smith; Mahi Patel; Divyam Goel, BS; Roberta Monardo, MD, PhD; Yazhong Tao, PhD

Search Librarian: Sarah Cantrell, PhD

I. Background

We and others recently showed that females with *Staphylococcus aureus* bloodstream infection have increased mortality relative to males. However, it is unknown whether this sex-specific difference in mortality is unique to *S. aureus* or extends to other infections. In this project, we aim address this issue by determining if sex-specific differences in patient mortality exist for additional organisms – gram-negative bacteria. The purpose of this project is to conduct a systematic review and meta-analysis assessing the association between female sex at birth and mortality in patients with gram-negative bacteremia.

Project Timeline
Project Start: December 17, 2024
Anticipated draft manuscript due: May 31, 2025
Anticipated manuscript submission: June 30, 2025

II. Key question

The key question for this systematic review is: For patients with gram-negative bacteremia, is female sex at birth associated with increased patient mortality?

A. Population
The population will be adults (age 18 years and older) with gram-negative bacteremia.

B. Intervention/Prognostic factor
The prognostic factor is female sex at birth.

C. Comparators
The comparator group will be male sex at birth patients.

D. Outcomes
The outcome will be mortality, up to 1 year.

© 2025 La P et al. *JAMA Network Open*.

III. Methods

A. Criteria for inclusion and exclusion of studies in this systematic review

Inclusion criteria. Included studies must meet all the following criteria:

1. Observational studies or randomized controlled trials
2. Report on adults aged 18 years or older
3. Study has greater than 100 patients with gram-negative bacteremia. Either single bacterial species (e.g., *Escherichia coli*) or a mixed population of gram-negative bacterial species is acceptable.
4. When mixed bacterial populations are reported (e.g., both gram-positive and gram-negative bacteria), stratification by gram-negative bacterial species is performed
5. Patients are stratified by sex at birth
6. Mortality in hospital or up to 90 day mortality is reported
7. English language publication
8. For inclusion in the primary analysis, studies must stratify patients by source of bacteremia or statistically adjust for source of bacteremia with an appropriate approach (e.g., regression analyses). Studies meeting criteria 1-7 but not 8 can still be included in the secondary analysis.

Exclusion criteria

1. No stratification of mortality by gender
2. Polymicrobial bloodstream infections

B. Literature search strategies

We will conduct a primary search of MEDLINE, Embase and Web of Science. We will further evaluate the bibliographies of any systematic or nonsystematic reviews for relevant studies. To ensure completeness, search strategies will be developed and executed by a medical librarian, with input on terms from the research team. Using pre-specified inclusion/exclusion criteria, titles and abstracts of articles included in the existing reviews and identified through our primary search will be reviewed independently by two reviewers for potential relevance. Articles included by any two reviewers will undergo full-text screening. Conflicts at this stage will be resolved by a third person. At the full-text screening stage, articles will be reviewed independently by two reviewers. The screeners must agree on a final inclusion/exclusion decision. Conflicts at this stage will be resolved by a third person. Articles meeting eligibility criteria will be included for data abstraction. All results will be tracked in Covidence, a web-based data synthesis software program, and, for manuscript preparation, EndNote X20 reference management software (Thomson Reuters).

C. Data abstraction and data management

Data from published reports will be abstracted into a customized Covidence data extraction form by one reviewer and overread by a second reviewer. Disagreements will be resolved by consensus or by obtaining a third reviewer's opinion when consensus

© 2025 La P et al. *JAMA Network Open*.

cannot be reached. Key characteristics abstracted will include patient descriptors (e.g., age, gender, race, level of acute illness, source of BSI, among others) and mortality. We will capture any subgroup analyses of particular interest.

D. Assessment of methodological quality of individual studies

Quality assessment will be performed by the researcher abstracting or evaluating the included article; this initial assessment will then be overread by a second reviewer. Disagreements will be resolved between the two reviewers or when needed by arbitration from a third reviewer. Risk of bias will be assessed with the Newcastle-Ottawa Scale for observational studies and Cochrane Criteria for randomized controlled trials. We will assign a summary quality score (low, medium, or high risk of bias) to individual studies.

E. Data synthesis

We will summarize the primary literature by abstracting the relevant data. We will develop a summary table describing the key outcomes and the types of study designs used to evaluate the association of sex at birth and mortality in patients with gram-negative bacteremia.

We will then determine the feasibility of completing a quantitative synthesis (i.e., meta-analysis) to estimate summary effects. Feasibility depends on the volume of relevant literature, conceptual homogeneity of the studies, and completeness of results reporting. We will aggregate outcomes when there are at least three studies with the same outcome, based on the rationale that one or two studies do not provide adequate evidence for summary effects.

Studies included in this systematic review will report the dichotomous outcome of mortality. If quantitative synthesis is possible, mortality will be reported using risk ratio or odds ratio. A large source of potential bias in this study is due to the impact of source of bacteremia on patient mortality. Bacteremia from a urinary source has been associated with decreased mortality in multiple studies and is more common in females. Thus the primary analysis will focus on studies that either statistically adjust for the source of bacteremia (e.g., regression model that accounts for source of bacteremia), matches males and females based on source of bacteremia, or performs a similar analysis that accounts for this variable. Secondary analyses will include unadjusted mortality and adjusted mortality that did not necessarily account for source of bacteremia as a model covariate. We will evaluate for statistical heterogeneity using visual inspection and Cochrane's Q and I^2 statistics. Publication bias will be assessed using findings from the ClinicalTrials.gov search (described previously) and using funnel plots (when there are >10 studies in an analysis).

If a quantitative synthesis is not feasible, we will analyze the data qualitatively. We will give more weight to the evidence from higher quality studies with more precise estimates

© 2025 La P et al. *JAMA Network Open*.

of effect. We will analyze potential reasons for inconsistency in mortality risk across studies by evaluating differences in the study population and outcome definitions.

F. Grading the evidence of the key question

The strength of evidence for the key question will be assessed using the approach described in AHRQ's "Methods Guide." In brief, this approach requires assessment of four domains: risk of bias, consistency, directness, and precision. Additional domains are to be used when appropriate: coherence, impact of plausible residual confounders, strength of association (magnitude of effect), and publication bias. These domains will be considered qualitatively, and a summary rating will be assigned after discussion by two providers as high, moderate, or low strength of evidence. In some cases, high, moderate, or low ratings will be impossible or imprudent to make. In these situations, a grade of insufficient will be assigned.

© 2025 La P et al. *JAMA Network Open*.

eAppendix 2. Search strategies

Librarian searcher: Sarah Cantrell, MLIS; Duke University Medical Center Library & Archives, Duke University School of Medicine

Peer-review of search conducted by: Samantha J. Kaplan, PhD MLIS; Duke University Medical Center Library & Archives, Duke University School of Medicine

Database: MEDLINE (via Ovid)
Search date: 1/8/2025
Note: Ovid MEDLINE® ALL 1946 to January 06, 2025

Search Set	Search Strategy	Results
#1 *Gram-negative bacteria terms*	exp Gram-Negative Bacteria/ OR exp Gram-Negative Bacterial Infections/ OR ("gram negative" OR gram-negative OR GNB OR GNBs OR enterobacteriaceae OR enterobacter OR enterobacterales OR escherichia OR "E.coli" OR "E. coli." OR "E coli" OR klebsiella OR proteus OR salmonella OR salmonellae OR serratia OR yersinia OR citrobacer OR morganella OR providencia OR stenotrophomonas OR moraxellaceae OR acinetobacter OR neisseriaceae OR gonorrhea OR meningococcal OR meningococcemia OR "neisseria meningitidis" OR pasteurellaceae OR actinobacillus OR actinobacillosis OR haemophilus OR pasteurella OR "hemorrhagic septicemia" OR "hemorrhagic septicaemia" OR "haemorrhagic septicaemia" OR "pneumonic pasteurellosis" OR pseudomonas).ti,ab.	1294230
#2 *Bacteremia terms*	Bacteremia/ OR (bacteremia OR bacteraemia OR bacteremic OR bacteraemic OR "bloodstream infection" OR "blood stream infection" OR "bloodstream infections" OR "blood stream infections" OR "blood infection" OR "blood infections" OR pyemia OR pyemic OR pyemias OR pyohemia OR pyohemias OR pyaemia OR payaemic).ti,ab.	62413
#3 *Mortality terms*	exp mortality/ or mortality.fs. or (mortality OR mortalities OR fatal OR fatality OR fatalities OR death OR deaths OR dying OR die OR died).ti,ab.	2710848
#4 *Combining*	1 and 2 and 3	9221
#5 *Animal exclusion*	4 not (exp animals/ not exp humans/)	8554
#6 *Pediatrics exclusion*	5 not ((exp adolescent/ or exp child/ or exp infant/) not exp adult/)	7352
#7 *Language filter*	6 and English.lg.	6866
#8 *Study design exclusion*	7 not (case reports OR editorial OR congress).pt.	6021
#9 *Review article exclusion – will be placed in separate Covidence*	8 not ("systematic review".pt. or review.pt. or "systematic review".ti. or "umbrella review.".ti. or "narrative review".ti. or "literature review".ti.)	5407

© 2025 La P et al. *JAMA Network Open*.

project for review		
#10<br>*Study design filter – trials, observational studies*	exp Evaluation Studies as Topic/ or exp Cohort Studies/ or exp Longitudinal Studies/ or exp Case-Control Studies/ or exp Cross-Sectional Studies/ or exp Controlled Before-After Studies/ or exp Interrupted Time Series Analysis/ or exp Prospective Studies/ or exp Retrospective Studies/ or exp Follow-Up Studies/ or "randomized controlled trial".pt. or "controlled clinical trial".pt. or clinical trial.pt. or "comparative study".pt. or "evaluation study".pt. or "observational study".pt. or (randomized or randomised or randomization or randomisation or placebo or randomly or trial or groups or "clinical trials" or "evaluation study" or "evaluation studies" or "intervention study" or "intervention studies" or cohort or cohorts or case-control* or "case control*" or cross-sectional* or "cross sectional*" or longitudinal or longitudinally or prospective or prospectively or retrospective or retrospectively or "follow up" or "follow-up" or "comparative study" or "comparative studies" or nonrandom or "non-random" or nonrandomized or "non-randomized" or nonrandomised or "non-randomised" or quasi-experiment* or quasiexperiment* or quasirandom* or quasi-random* or quasi-control* or quasicontrol* or "pre-post" or "pre post" or posttest or "post-test" or "post test" or pretest or "pre-test" or "pre test" or "repeated measure" or "repeated measures").ti,ab. or (before and after).ti,ab. or (before and during).ti,ab. or ("time series" and interrupt*).ti,ab. or ("time points" and (multiple or one or two or three or four or five or six or seven or eight or nine or ten or month or monthly or day or daily or week or weekly or hour or hourly)).ti,ab.	
#11<br>Final combination	9 and 10	4289
Validation set	11 AND ("29020307" or "19040476" or "32114010" or "38574775" or "12955633").ui.	5/5

Database: Embase (via Elsevier)

Search date: 1/8/2025

Note: search from results page

Search Set	Search Strategy	Results
#1<br>*Gram-negative bacteria terms*	'Gram negative bacterium'/exp OR 'Gram negative infection'/exp OR ('gram negative' OR gram-negative OR GNB OR GNBs OR enterobacteriaceae OR enterobacter OR enterobacterales OR escherichia OR 'Ecoli' OR 'E coli' OR 'E.coli' OR klebsiella OR proteus OR salmonella OR salmonellae OR serratia OR yersinia OR citrobacer OR morganella OR providencia OR stenotrophomonas OR moraxellaceae OR acinetobacter OR neisseriaceae OR gonorrhea OR meningococcal OR meningococcemia OR 'neisseria meningitidis' OR pasteurellaceae OR actinobacillus OR actinobacillosis OR haemophilus OR pasteurella OR 'hemorrhagic septicemia' OR 'hemorrhagic	1633557

© 2025 La P et al. *JAMA Network Open*.

	septicaemia' OR 'haemorrhagic septicaemia' OR 'pneumonic pasteurellosis' OR pseudomonas):ti,ab	
#2 *Bacteremia terms*	'bacteremia'/exp OR (bacteremia OR bacteraemia OR bacteremic OR bacteraemic OR 'bloodstream infection' OR 'blood stream infection' OR 'bloodstream infections' OR 'blood stream infections' OR 'blood infection' OR 'blood infections' OR pyemia OR pyemic OR pyemias OR pyohemia OR pyohemias OR pyaemia OR payaemic):ti,ab	104669
#3 *Mortality terms*	'mortality'/exp OR mortality.fs. OR (mortality OR mortalities OR fatal OR fatality OR fatalities OR death OR deaths OR dying OR die OR died):ti,ab	3818378
#4 *Combining*	#1 AND #2 AND #3	17423
#5 *Animal exclusion*	#4 AND [humans]/lim	15307
#6 *Pediatrics exclusion*	#5 NOT (('adolescent'/exp OR 'child'/exp OR 'infant'/exp) NOT 'adult'/exp)	13153
#7 *Language filter*	#6 AND [english]/lim	12514
#8 *Study design exclusion*	#7 NOT ('case report'/exp OR 'case study'/exp OR 'editorial'/exp OR [editorial]/lim OR 'note'/exp OR [note]/lim OR [conference abstract]/lim OR 'conference abstract'/exp OR 'conference abstract'/it)	7991
#9 *Review article exclusion – will be placed in separate Covidence project for review*	#8 NOT ('review'/exp OR 'systematic review':ti OR 'umbrella review.':ti OR 'narrative review':ti OR 'literature review':ti)	6846
#10 *Study design filter – trials, observational studies*	'randomized controlled trial'/exp OR 'crossover procedure'/exp OR 'double blind procedure'/exp OR 'single blind procedure'/exp OR 'clinical study'/exp OR 'controlled study'/exp OR 'evaluation study'/exp OR 'intervention study'/exp OR 'cohort analysis'/exp OR 'follow up'/exp OR 'comparative effectiveness'/exp OR 'longitudinal study'/exp OR 'evaluation study'/exp OR 'prospective study'/exp OR 'time series analysis'/exp OR 'cross-sectional study'/exp OR 'case control study'/exp OR 'retrospective study'/exp OR (randomized OR randomised OR randomization OR randomisation OR placebo OR randomly OR trial OR groups OR 'clinical trials' OR 'evaluation study' OR 'evaluation studies' OR 'intervention study' OR 'intervention studies' OR cohort OR cohorts OR 'case-control' OR 'case-controls' OR 'case-controlled' OR 'case control' OR 'case controls' OR 'case controlled' OR 'cross-sectional' OR 'cross sectional' OR 'cross-sectionally' OR 'cross sectionally' OR longitudinal OR longitudinally OR prospective OR prospectively OR retrospective OR retrospectively OR 'follow up' OR 'follow-up' OR 'comparative study' OR 'comparative studies' OR nonrandom OR 'non-random' OR nonrandomized OR 'non-randomized' OR nonrandomised OR 'non-randomised' OR 'quasi-experiment' OR 'quasi experiment' OR 'quasi-experimental' OR 'quasi experimental'	23302034

	OR quasirandom* OR 'quasi-randomized' OR 'quasi randomized' OR 'quasi-randomised' OR 'quasi randomised' OR 'quasi-controlled' OR 'quasi controlled' OR quasicontrol* OR 'pre-post' OR 'pre post' OR posttest OR 'post-test' OR 'post test' OR pretest OR 'pre-test' OR 'pre test' OR 'repeated measure' OR 'repeated measures'):ti,ab OR (before AND after):ti,ab OR (before AND during):ti,ab OR ('time series' AND interrupt*):ti,ab OR ('time points' AND (multiple OR one OR two OR three OR four OR five OR six OR seven OR eight OR nine OR ten OR month OR monthly OR day OR daily OR week OR weekly OR hour OR hourly)):ti,ab	
#11 Final combination	#9 AND #10	6197

Database: Web of Science – Science Citation Index Expanded (1900 – present) and Emerging Sources Citation Index (2019-present)

Search date:

Note: select indices under Editions; use Advanced Search

Search Set	Search Strategy	Results
#1 *Gram-negative bacteria terms*	TS=("gram negative" OR gram-negative OR GNB OR GNBs OR enterobacteriaceae OR enterobacter OR enterobacterales OR escherichia OR "E.coli" OR "E. coli." OR "E coli" OR klebsiella OR proteus OR salmonella OR salmonellae OR serratia OR yersinia OR citrobacer OR morganella OR providencia OR stenotrophomonas OR moraxellaceae OR acinetobacter OR neisseriaceae OR gonorrhea OR meningococcal OR meningococcemia OR "neisseria meningitidis" OR pasteurellaceae OR actinobacillus OR actinobacillosis OR haemophilus OR pasteurella OR "hemorrhagic septicemia" OR "hemorrhagic septicaemia" OR "haemorrhagic septicaemia" OR "pneumonic pasteurellosis" OR pseudomonas)	1054118
#2 *Bacteremia terms*	TS=(bacteremia OR bacteraemia OR bacteremic OR bacteraemic OR "bloodstream infection" OR "blood stream infection" OR "bloodstream infections" OR "blood stream infections" OR "blood infection" OR "blood infections" OR pyemia OR pyemic OR pyemias OR pyohemia OR pyohemias OR pyaemia OR payaemic)	72076
#3 *Mortality terms*	TS=(mortality OR mortalities OR fatal OR fatality OR fatalities OR death OR deaths OR dying OR die OR died)	3132289
#4 *Combining*	#1 AND #2 AND #3	9454
#5 *Animal exclusion*	#4 NOT TI=(animal OR animals OR porcine OR pig OR pigs OR equine OR horse OR horses OR murine OR mouse OR mice OR Rodentia OR rat OR rats OR canine OR dog OR dogs OR feline OR cat OR cats)	9122
#6 *Language filter*	Refined by Languages: English	8833

© 2025 La P et al. *JAMA Network Open*.

#7<br>*Study design exclusion*	Exclude Document Types: Editorial Material OR Meeting Abstract OR Book Chapter OR Reprint OR Data Paper OR Retracted Publication OR Note)	8698
#8	#7 NOT TI=("case report" OR "case reports" OR "case study")	8541
#9<br>*Review article exclusion – will be placed in separate Covidence project for review*	Exclude Document Types: Review Article	7710
#10	#9 NOT TI=("systematic review" OR "systematic reviews" OR "scoping review" OR "scoping reviews" OR "literature review" OR "literature reviews" OR "narrative review" OR "narrative reviews" OR meta-analysis OR "umbrella review")	7662
#11<br>*Study design filter – trials, observational studies*	TS=(randomized OR randomised OR randomization OR randomisation OR placebo OR randomly OR trial OR groups OR "clinical trials" OR "evaluation study" OR "evaluation studies" OR "intervention study" OR "intervention studies" OR cohort OR cohorts OR case-control* OR "case control*" OR cross-sectional* OR "cross sectional*" OR longitudinal OR longitudinally OR prospective OR prospectively OR retrospective OR retrospectively OR "follow up" OR "follow-up" OR "comparative study" OR "comparative studies" OR nonrandom OR "non-random" OR nonrandomized OR "non-randomized" OR nonrandomised OR "non-randomised" OR quasi-experiment* OR quasiexperiment* OR quasirandom* OR quasi-random* OR quasi-control* OR quasicontrol* OR "pre-post" OR "pre post" OR posttest OR "post-test" OR "post test" OR pretest OR "pre-test" OR "pre test" OR "repeated measure" OR "repeated measures") OR TS=(before and after) OR TS=(before and during) OR TS=("time series" and interrupt*) OR TS=(("time points" and (multiple OR one OR two OR three OR four OR five OR six OR seven OR eight OR nine OR ten OR month OR monthly OR day OR daily OR week OR weekly OR hour OR hourly)))	11656632
#12<br>Final combination	#10 AND #11	5316

© 2025 La P et al. *JAMA Network Open*.

eAppendix 3. Newcastle-Ottawa Quality Assessment Scale for assessing risk of bias in cohort and case-control studies

The detailed criteria used to assess these study types, as well as the thresholds for converting the Newcastle-Ottawa scale to AHRQ standards (good, fair, and poor), are shown below.

A. Cohort studies

Selection

1. Representativeness of the exposed cohort
   a. Truly representative of the average person with gram-negative bloodstream infection in the community (*)
   b. Somewhat representative of the average person with gram-negative bloodstream infection in the community (*)
   c. Selected group of patients
   d. No description of the derivation of the cohort

2. Selection of the non-exposed cohort
   a. Drawn from the same community as the exposed cohort (*)
   b. Drawn from a different source
   c. No description of the derivation of the non-exposed cohort

3. Ascertainment of exposure
   a. Secure record (e.g. medical records) (*)
   b. Structured interview (*)
   c. Written self report
   d. No description

4. Demonstration that outcome of interest was not present at start of study
   a. Yes (*)
   b. No

Comparability of cohorts on basis of design or analysis

1. Study controls for at least one factor besides biological sex
   a. Yes (*)
   b. No

2. Study does not control for acute severity of illness
   a. Yes (*)
   b. No

Outcome

1. Assessment of outcome
   a. Independent blind assessment (*)
   b. Record linkage (*)
   c. Self report
   d. No description

2. Was follow-up long enough for outcomes to occur
    a. Yes (In-hospital mortality or mortality ≤30 days) (*)
    b. No

3. Adequacy of follow up of cohorts
    a. Complete follow up (all subjects accounted for) (*)
    b. Subjects lost to follow up unlikely to introduce bias (≤10% lost to follow-up, or description provided of those lost) (*)
    c. Follow up rate < 90% and no description of those lost
    d. No statement

Thresholds used to convert the Newcastle-Ottawa scale to AHRQ standards (good, fair, and poor):

Good quality/low risk of bias: 3 or 4 stars in selection domain AND 1 or 2 stars in comparability domain AND 2 or 3 stars in outcome/exposure domain

Fair quality/medium risk of bias: 2 stars in selection domain AND 1 or 2 stars in comparability domain AND 2 or 3 stars in outcome/exposure domain.

Poor quality/high risk of bias: 0 or 1 star in selection domain OR 0 stars in comparability domain OR 0 or 1 stars in outcome/exposure domain

B. Case-control studies

Selection

1. Is the case definition adequate?
    a. Yes, with independent validation (*)
    b. Yes, with record linkage or based on self-reports
    c. No description

2. Representativeness of the cases
    a. Consecutive or obviously representative series of cases (*)
    b. Potential for selection biases or not stated

3. Selection of Controls
    a. Community controls (*)
    b. Hospital controls
    c. No description

4. Definition of Controls
    a. No history of disease (endpoint) (*)
    b. No description of source

Comparability of cases and controls on the basis of the design or analysis

1. Study controls for at least one factor besides biological sex
    a. Yes (*)

© 2025 La P et al. *JAMA Network Open*.

    b. No

2. Study does not control for acute severity of illness
    a. Yes (*)
    b. No

Exposure

1. Ascertainment of exposure
    a. Secure record (e.g. medical records) (*)
    b. Structured interview where blind to case/control status (*)
    c. Interview not blinded to case/control status
    d. Written self-report or medical record only
    e. No description

2. Same method of ascertainment for cases and controls
    a. Yes (*)
    b. No

3. Non-Response rate
    a. Same rate for both groups (*)
    b. Non respondents described
    c. Rate different and no designation

Thresholds used to convert the Newcastle-Ottawa scale to AHRQ standards (good, fair, and poor):

Good quality/low risk of bias: 3 or 4 stars in selection domain AND 1 or 2 stars in comparability domain AND 2 or 3 stars in outcome/exposure domain

Fair quality/medium risk of bias: 2 stars in selection domain AND 1 or 2 stars in comparability domain AND 2 or 3 stars in outcome/exposure domain.

Poor quality/high risk of bias: 0 or 1 star in selection domain OR 0 stars in comparability domain OR 0 or 1 stars in outcome/exposure domain

© 2025 La P et al. *JAMA Network Open*.

eAppendix 4. Approach for grading the overall strength of evidence

We used the Evidence-based Practice Center (EPC) model from the U.S. Agency for Healthcare Research and Quality (AHRQ) to grade the overall strength of evidence.[1] The EPC approach evaluates the following domains: study limitations/risk of bias, consistency, directness, precision, and reporting bias. In brief, the EPC classification system applies an overall strength of evidence grade rating to an estimate effect from a body of evidence: high (we are very confident that the estimate of effect lies close to the true effect for this outcome), moderate (we are moderately confident that the estimate of effect lies close to the true effect for this outcome), low (we have limited confidence that the estimate of effect lies close to the true effect for this outcome), or insufficient (we have no evidence, we are unable to estimate an effect, or we have no confidence in the estimate of effect for this outcome). The initial strength of evidence grade was moderate given that the included observational studies in the primary analysis reduced bias from confounding through matching or statistical adjustment.[1] This baseline category could be rated down if the included studies demonstrated high risk of bias, imprecision, inconsistency, indirectness, or reporting bias.

© 2025 La P et al. *JAMA Network Open*.

eAppendix 5. Risk of bias (quality) assessment of included cohort studies using the Newcastle-Ottawa Quality Assessment Scale

The Newcastle-Ottawa Quality Assessment Scale determines a study's risk of bias through nine questions (detailed in eAppendix 3). For each study, the grades for the nine questions are shown below. Grades that receive a star are highlighted in green, while those that do not are highlighted in red. Based on the grades from each question in the Newcastle-Ottawa Scale, an overall risk of bias (high, medium, low) can be assigned.

Study	**Selection:** Representativeness of the exposed cohort	**Selection:** Selection of non-exposed cohort	**Selection:** Ascertainment of exposure	**Selection:** Outcome not present at start of study	**Comparability:** Controls for level of acute illness	**Comparability:** Controls for any additional factor	**Outcome:** Assessment of outcome	**Outcome:** Was follow-up long enough	**Outcome:** Adequacy of follow-up	Risk of bias
Abdel Hadi 2024	B	A	A	A	A	A	A	A	A	Low
Alamgir 2006	B	A	A	A	A	A	A	A	A	Low
Al-Hasan 2010	B	A	A	A	A	A	A	A	A	Low
Ayaz 2024	C	A	A	A	A	A	A	A	A	Low
Blandy 2019	B	A	A	A	A	A	A	A	A	Low
Chen 2021	C	A	A	A	A	A	A	A	A	Low
Chen 2023	C	A	A	A	A	A	A	A	A	Low
Chiong 2021	B	A	A	A	A	A	A	A	A	Low
Corcione 2022	C	A	A	A	A	A	A	A	A	Low
Derin 2024	C	A	A	A	A	A	A	A	A	Low
Dumlu 2024	C	A	A	A	A	A	A	A	A	Low
Fostervold 2024	B	A	A	A	A	A	A	A	B	Low
Hojat 2024	B	A	A	A	A	A	A	A	A	Low
Huggins 2023	C	A	A	A	A	A	A	A	A	Low
Jauneikaite 2022	B	A	A	A	A	A	A	A	A	Low

© 2025 La P et al. *JAMA Network Open*.

Khamis 2022	C	A	A	A	A	A	A	A	A	Low
Kim 2014	A	A	A	A	A	A	A	A	A	Low
Man 2017	B	A	A	A	A	A	A	A	A	Low
Mert 2024	C	A	A	A	A	A	A	A	A	Low
Onorato 2022	C	A	A	A	A	A	A	A	A	Low
Pedersen 1997	B	A	A	A	A	A	A	A	B	Low
Scheich 2018	C	A	A	A	A	A	A	A	A	Low
Swingler 2024	C	A	A	A	A	A	A	A	A	Low
Tinevez 2022	B	A	A	A	A	A	A	A	A	Low
Yang 2024	B	A	A	A	A	A	A	A	A	Low

© 2025 La P et al. *JAMA Network Open*.

eAppendix 6. Summary of studies included in the secondary (unadjusted mortality) analysis

	Studies, No. (%) (n=321)	Patients, No. (%) (n=147,810)
**Publication year**		
Before 2000	6 (1.9)	1,769 (1.2)
2000-2009	18 (5.6)	3,923 (2.7)
2010-2019	141 (43.9)	61,885 (41.9)
2020-2025	156 (48.6)	80,233 (54.3)
**Study design**		
Cohort study	307 (95.6)	143,704 (97.2)
Case control	11 (3.4)	2,005 (1.4)
Retrospective quasi-experimental	1 (0.3)	200 (0.1)
Cross sectional	1 (0.3)	1,608 (1.1)
Randomized control trial	1 (0.3)	293 (0.2)
**Continent**		
Asia	182 (56.7)	52,457 (35.5)
Europe	87 (27.1)	55,696 (37.7)
North America	35 (10.9)	32,014 (21.7)
South America	8 (2.5)	1,291 (0.8)
Multiple	6 (1.9)	4,506 (3.0)
Oceania	2 (0.6)	238 (0.2)
Africa	1 (0.3)	1,608 (1.1)
**No. of hospitals included**		
1	215 (67.0)	68,179 (46.1)
2-19	86 (26.8)	29,533 (20.0)
20-99	14 (4.4)	7,071 (4.8)
≥ 100	6 (1.9)	43,027 (29.1)
**No. of patients included**		
100-999	301 (93.8)	78,601 (53.2)
1000-9,999	18 (5.6)	42,361 (28.7)
$\geq 10,000$	2 (0.6)	26,848 (18.2)
**Bacterial species**		
Enterobacterales	176 (54.8)	86,782 (58.7)
All gram-negative bacteria	44 (13.7)	24,281 (16.4)
Non-lactose fermenters	92 (28.7)	31,579 (21.4)
Mixed species	9 (2.8)	5,168 (3.5)
**Mortality outcome measure***		
In-hospital	47 (14.7)	45,286 (30.7)
5-7-days	10 (3.1)	3,543 (2.4)
14-15-days	31 (9.7)	6,676 (4.5)
21-days	4 (1.2)	827 (0.6)
28-30-days	229 (71.3)	90,276 (61.1)
60-days	2 (0.6)	1,368 (0.9)
90-days	5 (1.6)	3,026 (2.0)

*papers can report multiple mortality timepoints

© 2025 La P et al. *JAMA Network Open*.

eAppendix 7. Influence analysis of studies included in the primary analysis (n=25)

Exclusion of any single study did not alter the overall results of the meta-analysis.

	OR	95%-CI	p-value	tau^2	tau	I^2	95%-PI
Omitting Abdel Hadi 2024	0.9739	[0.8050; 1.1782]	0.7767	0.0959	0.3096	48.1%	[0.4979; 1.9051]
Omitting Alamgir 2006	1.0354	[0.8709; 1.2310]	0.6812	0.0545	0.2334	32.3%	[0.6188; 1.7324]
Omitting Al-Hasan 2010	0.9839	[0.8135; 1.1900]	0.8616	0.0949	0.3080	48.3%	[0.5049; 1.9174]
Omitting Ayaz 2024	0.9789	[0.8097; 1.1834]	0.8180	0.0944	0.3073	48.3%	[0.5031; 1.9047]
Omitting Blandy 2019	0.9707	[0.8001; 1.1777]	0.7530	0.0998	0.3159	47.8%	[0.4894; 1.9252]
Omitting Chen 2023	0.9603	[0.8013; 1.1507]	0.6472	0.0858	0.2930	44.9%	[0.5082; 1.8144]
Omitting Chiong 2021	0.9925	[0.8224; 1.1977]	0.9344	0.0926	0.3043	47.5%	[0.5132; 1.9192]
Omitting Corcione 2022	0.9795	[0.8063; 1.1898]	0.8273	0.1025	0.3202	48.3%	[0.4895; 1.9600]
Omitting Derin 2024	0.9808	[0.8099; 1.1877]	0.8356	0.0972	0.3117	48.3%	[0.4992; 1.9269]
Omitting Dumlu 2024	0.9849	[0.8135; 1.1924]	0.8704	0.0967	0.3109	48.3%	[0.5022; 1.9315]
Omitting Fostervold 2024	0.9710	[0.8003; 1.1780]	0.7554	0.0998	0.3159	47.8%	[0.4896; 1.9257]
Omitting Hojat 2024	0.9737	[0.8037; 1.1797]	0.7764	0.0980	0.3131	48.1%	[0.4941; 1.9190]
Omitting Huggins 2023	1.0084	[0.8379; 1.2136]	0.9265	0.0851	0.2918	44.2%	[0.5347; 1.9015]
Omitting Jauneikaite 2022	0.9954	[0.8242; 1.2021]	0.9598	0.0930	0.3050	47.2%	[0.5138; 1.9284]
Omitting Khamis 2022	0.9597	[0.8000; 1.1513]	0.6448	0.0860	0.2933	45.0%	[0.5076; 1.8145]
Omitting Kim 2014	0.9826	[0.8141; 1.1861]	0.8490	0.0912	0.3021	48.3%	[0.5108; 1.8904]
Omitting Kousouli 2019	0.9684	[0.7995; 1.1729]	0.7318	0.0970	0.3114	47.5%	[0.4929; 1.9024]
Omitting Man 2017	0.9358	[0.7851; 1.1155]	0.4427	0.0527	0.2296	30.1%	[0.5634; 1.5544]
Omitting Mert 2024	0.9777	[0.8079; 1.1832]	0.8090	0.0962	0.3101	48.3%	[0.4994; 1.9142]
Omitting Onorato 2022	1.0005	[0.8309; 1.2047]	0.9954	0.0893	0.2989	46.1%	[0.5231; 1.9137]
Omitting Pedersen 1997	0.9872	[0.8124; 1.1996]	0.8926	0.1026	0.3204	47.9%	[0.4931; 1.9766]
Omitting Scheich 2018	0.9726	[0.8056; 1.1741]	0.7627	0.0934	0.3056	47.8%	[0.5016; 1.8858]
Omitting Swingler 2024	1.0088	[0.8426; 1.2077]	0.9208	0.0824	0.2870	43.6%	[0.5405; 1.8828]
Omitting Tinevez 2022	1.0134	[0.8477; 1.2115]	0.8789	0.0785	0.2802	42.2%	[0.5505; 1.8654]
Omitting Yang 2024	0.9692	[0.8048; 1.1670]	0.7304	0.0913	0.3022	47.1%	[0.5034; 1.8659]
Random effects model (HK)	0.9824	[0.8178; 1.1801]	0.8432	0.0904	0.3007	46.1%	[0.5129; 1.8817]

© 2025 La P et al. *JAMA Network Open*.

eAppendix 8. Funnel plot of studies included in the primary analysis
No significant publication bias was observed (p=0.58).

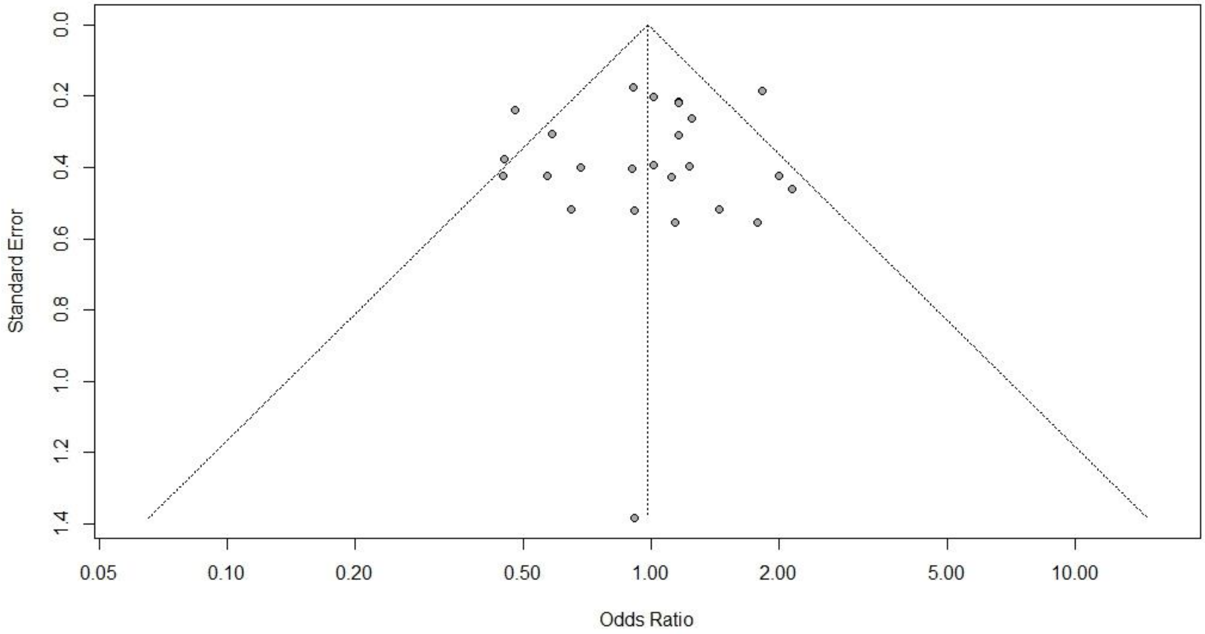

© 2025 La P et al. *JAMA Network Open*.

eAppendix 9. Exploratory analysis of all studies that reported adjusted sex-stratified mortality data in patients with gram-negative bloodstream infection

No association between biological sex and mortality was identified.

Study	logOR	SE(logOR)	OR	95%-CI	Weight
Abdel Hadi 2024	0.2070	0.3975	1.23	[0.56; 2.68]	1.4%
Alamgir 2006	-0.7367	0.2378	0.48	[0.30; 0.76]	2.4%
Al-Hasan 2010	-0.0931	0.5220	0.91	[0.33; 2.53]	1.0%
Aryee 2020	-0.2231	0.2581	0.80	[0.48; 1.33]	2.3%
Atamna 2019	-1.3863	0.3536	0.25	[0.13; 0.50]	1.6%
Ayaz 2024	0.1278	0.5557	1.14	[0.38; 3.38]	0.9%
Babich 2020	0.2927	0.1381	1.34	[1.02; 1.76]	3.2%
Battle 2019	0.0886	0.2315	1.09	[0.69; 1.72]	2.5%
Blandy 2019	0.1508	0.2148	1.16	[0.76; 1.77]	2.6%
Boattini 2023	0.4943	0.3994	1.64	[0.75; 3.59]	1.4%
Camins 2011	-0.6931	0.4571	0.50	[0.20; 1.22]	1.2%
Chapelet 2017	-0.1625	0.7968	0.85	[0.18; 4.05]	0.5%
Chen 2020	-0.3436	0.1079	0.71	[0.57; 0.88]	3.5%
Chen 2021	0.2231	0.4571	1.25	[0.51; 3.06]	1.2%
Chen 2023	0.7674	0.4594	2.15	[0.88; 5.30]	1.2%
Cheong 2008	-0.5621	0.3826	0.57	[0.27; 1.21]	1.5%
Chiong 2021	-0.4318	0.5188	0.65	[0.23; 1.80]	1.0%
Corcione 2022	0.0129	0.2033	1.01	[0.68; 1.51]	2.7%
Derin 2024	0.0133	0.3918	1.01	[0.47; 2.18]	1.4%
Dumlu 2024	-0.1006	0.4043	0.90	[0.41; 2.00]	1.4%
Falcone 2023	0.1985	0.1680	1.22	[0.88; 1.70]	3.0%
Florez Riano 2024	-0.5570	0.7983	0.57	[0.12; 2.74]	0.5%
Fostervold 2024	0.1484	0.2189	1.16	[0.76; 1.78]	2.6%
Hojat 2024	0.1508	0.3082	1.16	[0.64; 2.13]	1.9%
Huggins 2023	-0.5365	0.3077	0.58	[0.32; 1.07]	1.9%
Huh 2020	0.1740	0.1905	1.19	[0.82; 1.73]	2.8%
Jauneikaite 2022	-0.3857	0.4005	0.68	[0.31; 1.49]	1.4%
Kanchanasuwan 2022	0.6349	0.5489	1.89	[0.64; 5.53]	0.9%
Khamis 2022	0.6931	0.4219	2.00	[0.87; 4.57]	1.3%
Kim 2014	-0.0888	1.3831	0.92	[0.06; 13.76]	0.2%
Kim 2019	-0.1674	0.2680	0.85	[0.50; 1.43]	2.2%
Lee 2010	-0.0583	0.4991	0.94	[0.35; 2.51]	1.0%
Lee 2021 (1)	-0.1278	0.5019	0.88	[0.33; 2.35]	1.0%
Lee 2021 (2)	-0.5596	0.4333	0.57	[0.24; 1.34]	1.3%
Lines 2019	-0.2954	0.2270	0.74	[0.48; 1.16]	2.5%
Lye 2012	-0.5596	0.2333	0.57	[0.36; 0.90]	2.4%
Man 2017	0.6057	0.1857	1.83	[1.27; 2.64]	2.8%
Maskarinec 2020	-0.0390	0.1084	0.96	[0.78; 1.19]	3.5%
Meng 2021	0.4650	0.4648	1.59	[0.64; 3.96]	1.2%
Mert 2024	0.1130	0.4277	1.12	[0.48; 2.59]	1.3%
Montero 2020	-0.1308	0.2898	0.88	[0.50; 1.55]	2.0%
Onorato 2022	-0.5636	0.4237	0.57	[0.25; 1.31]	1.3%
Park 2022	0.0991	0.4006	1.10	[0.50; 2.42]	1.4%
Pedersen 1997	-0.0953	0.1768	0.91	[0.64; 1.29]	2.9%
Pena 2015	-0.3669	0.3166	0.69	[0.37; 1.29]	1.9%
Perez-Nadales 2023	0.9074	0.4290	2.48	[1.07; 5.74]	1.3%
Sathya Kumar 2023	-0.4968	0.4246	0.61	[0.26; 1.40]	1.3%
Scheich 2018	0.3707	0.5160	1.45	[0.53; 3.98]	1.0%
Scheuerman 2018 (Ec)	-0.0770	0.2896	0.93	[0.52; 1.63]	2.0%
Scheuerman 2018 (Kp)	0.3133	0.2279	1.37	[0.88; 2.14]	2.5%
Swingler 2024	-0.8019	0.4235	0.45	[0.20; 1.03]	1.3%
Tang 2021	-0.1354	0.2990	0.87	[0.49; 1.57]	2.0%
Tinevez 2022	-0.7985	0.3769	0.45	[0.21; 0.94]	1.5%
Trecarichi 2019	-2.1582	0.8052	0.12	[0.02; 0.56]	0.5%
Wu 2012	-0.1398	0.4016	0.87	[0.40; 1.91]	1.4%
Xu 2022	1.0996	0.3705	3.00	[1.45; 6.21]	1.6%
Yang 2024	0.5798	0.5529	1.79	[0.60; 5.28]	0.9%
Zhao 2022	0.4308	0.4875	1.54	[0.59; 4.00]	1.1%
Zhao 2023	-0.4511	0.5663	0.64	[0.21; 1.93]	0.9%
Zhou 2021	-0.2191	0.5024	0.80	[0.30; 2.15]	1.0%
**Random effects model (HK)**			**0.94**	**[0.83; 1.06]**	**100.0%**
**Prediction interval**				**[0.51; 1.74]**	

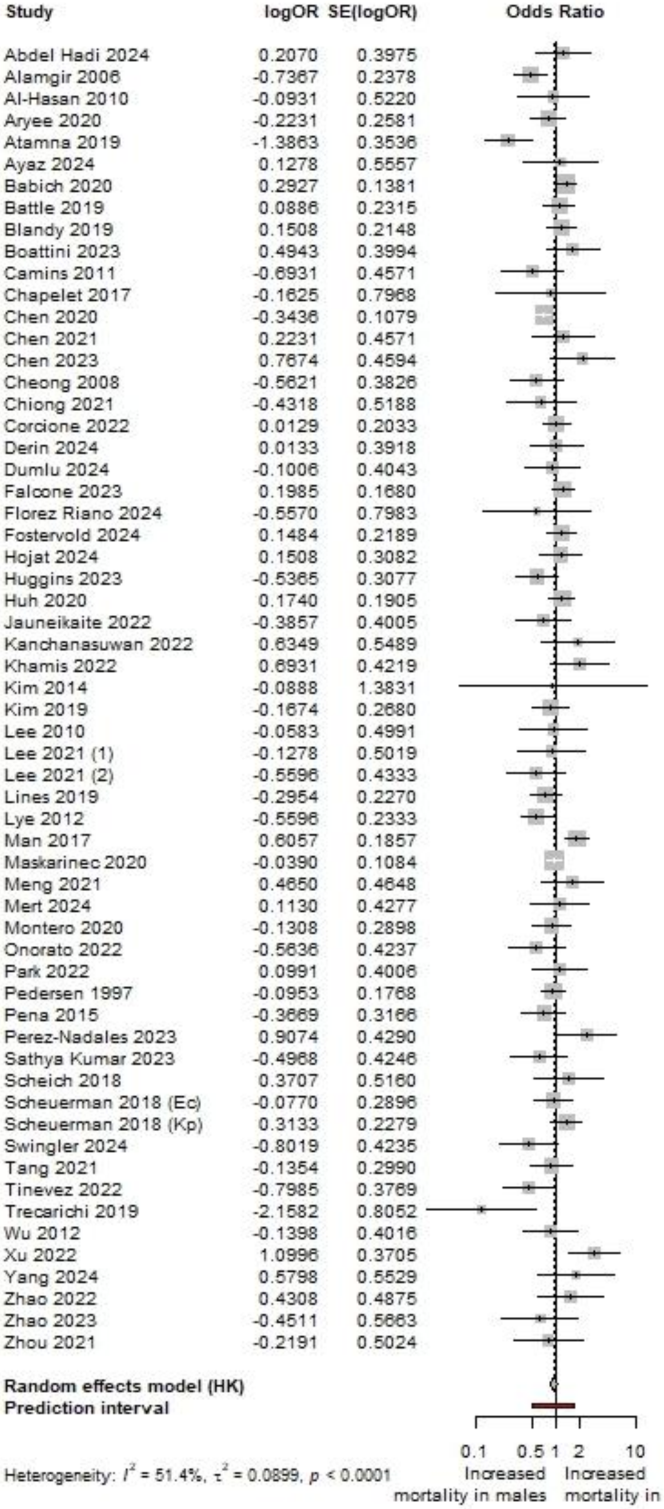

Heterogeneity: $I^2 = 51.4\%$, $\tau^2 = 0.0899$, $p < 0.0001$

© 2025 La P et al. *JAMA Network Open*.

eAppendix 10. Subset meta-analyses, stratified by clinical variables
Prior work has shown that variables including age, source of bloodstream infection, and medical comorbidities impact patient mortality in Gram-negative bloodstream infections, and can potentially differ between male and female patients.[2–4] Therefore, we performed subset meta-analyses of studies that adjusted for these variables. All studies in the primary analysis adjusted for age, and hence this subset analysis was not necessary. Subset analysis of studies that adjusted for source of bloodstream infection (A) did not reveal an association between biological sex and mortality. Subset analysis of studies that adjusted for medical comorbidities was complicated by heterogeneity in included disease states. Therefore, we performed a subset analysis of all studies that included a weighted index measure to describe the burden of medical comorbidities. Here, these measures included the Charlson comorbidity index,[5] Elixhauser index,[6] and McCabe score.[7] Meta-analysis of this subset of studies (B) did not reveal an association between biological sex and mortality. Only a limited number of studies adjusted for age, source of bloodstream infection, and a medical comorbidity index. A meta-analysis of these studies did not reveal an association between biological sex and mortality (C).

A.

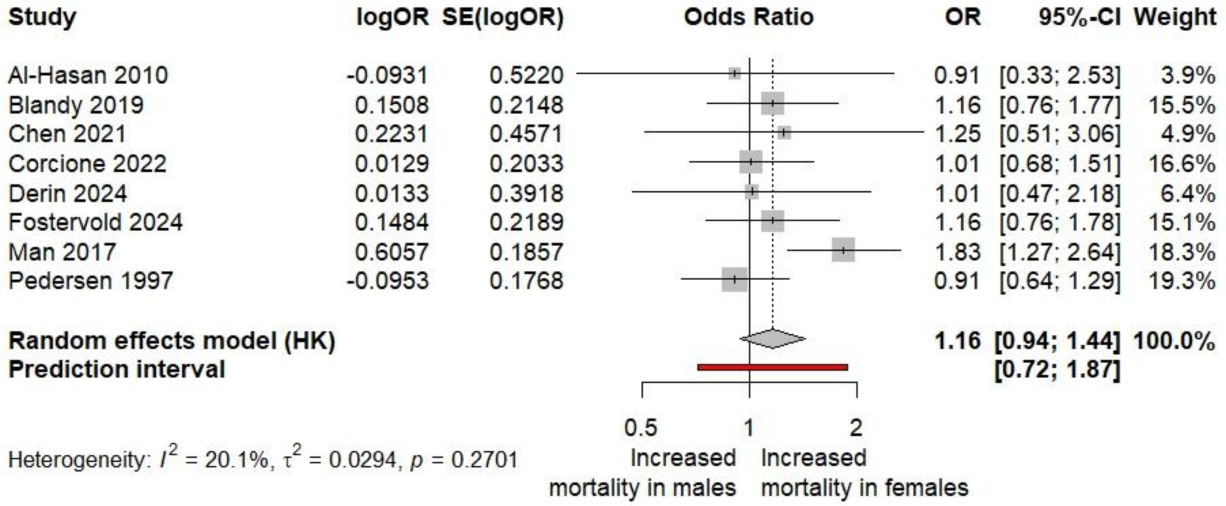

B.

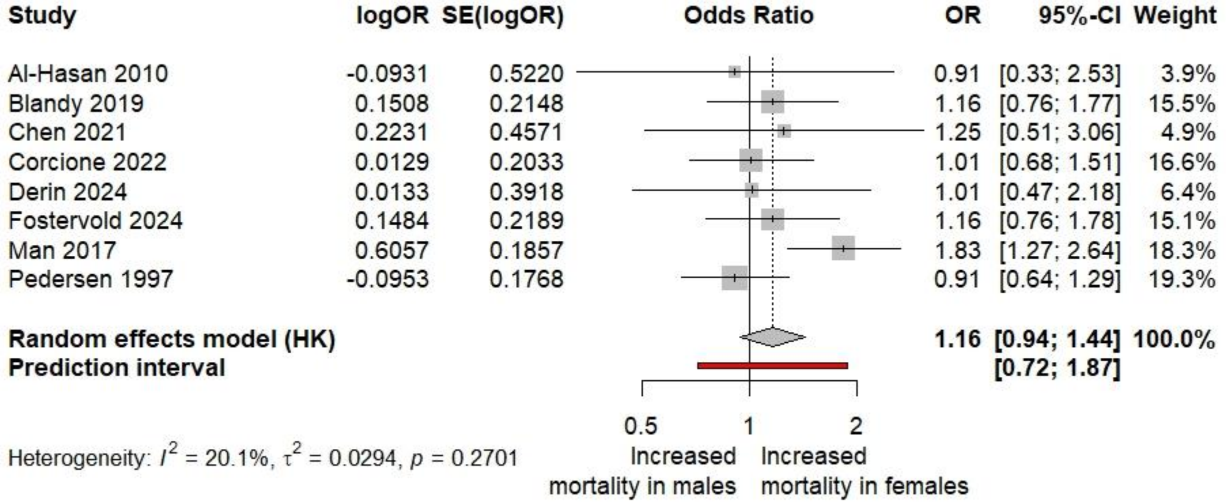

© 2025 La P et al. *JAMA Network Open*.

C.

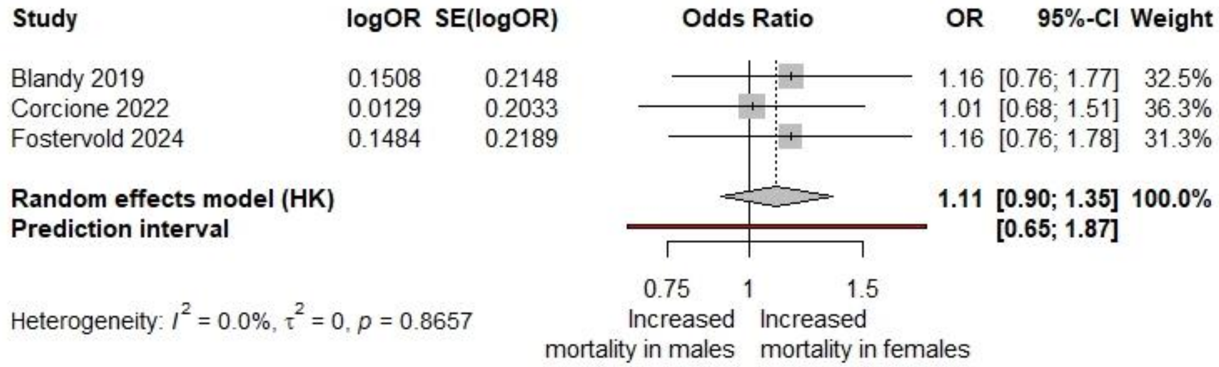

© 2025 La P et al. *JAMA Network Open*.

eAppendix 11. Subset meta-analyses, stratified by timing of mortality endpoint

Studies included in the primary analysis that reported mortality data from 28 to 30 days from the initial positive blood culture are shown here. No association between female sex and mortality at this endpoint was identified. Subset analyses of studies that addressed mortality at other endpoints (e.g., 90 days, in-hospital mortality, etc.) were not performed as there were no more than 2 such studies for each of these endpoints.

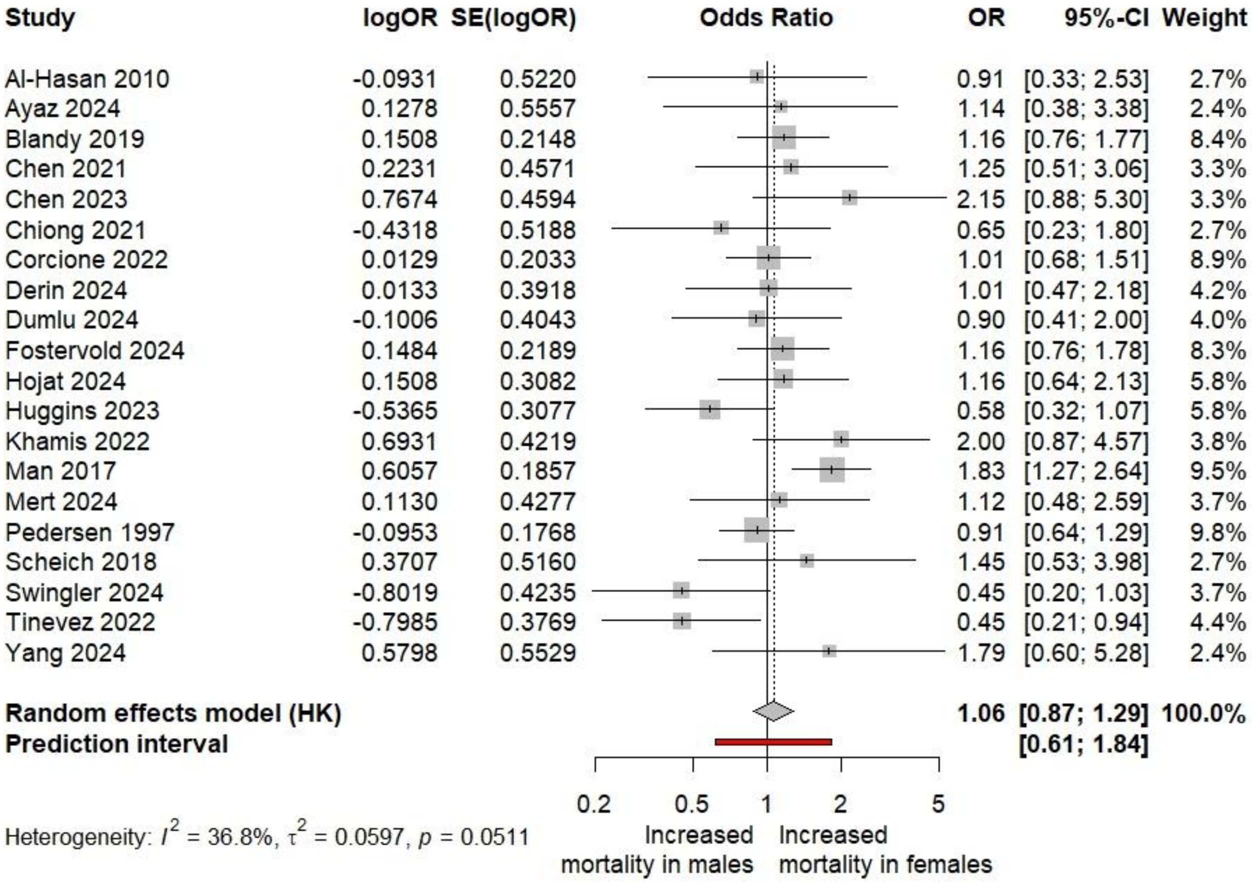

© 2025 La P et al. *JAMA Network Open*.

eAppendix 12. Subset meta-analyses, stratified by bacterial species group

Studies included in the primary analysis that reported mortality data from patients with Enterobacterales bloodstream infection only (A), non-lactose-fermenting gram-negative bloodstream infection only (B), or from a mixture of Enterobacterales and non-lactose-fermenters (C) are shown here. No association between biological sex and patient mortality was identified in these subsets.

A.

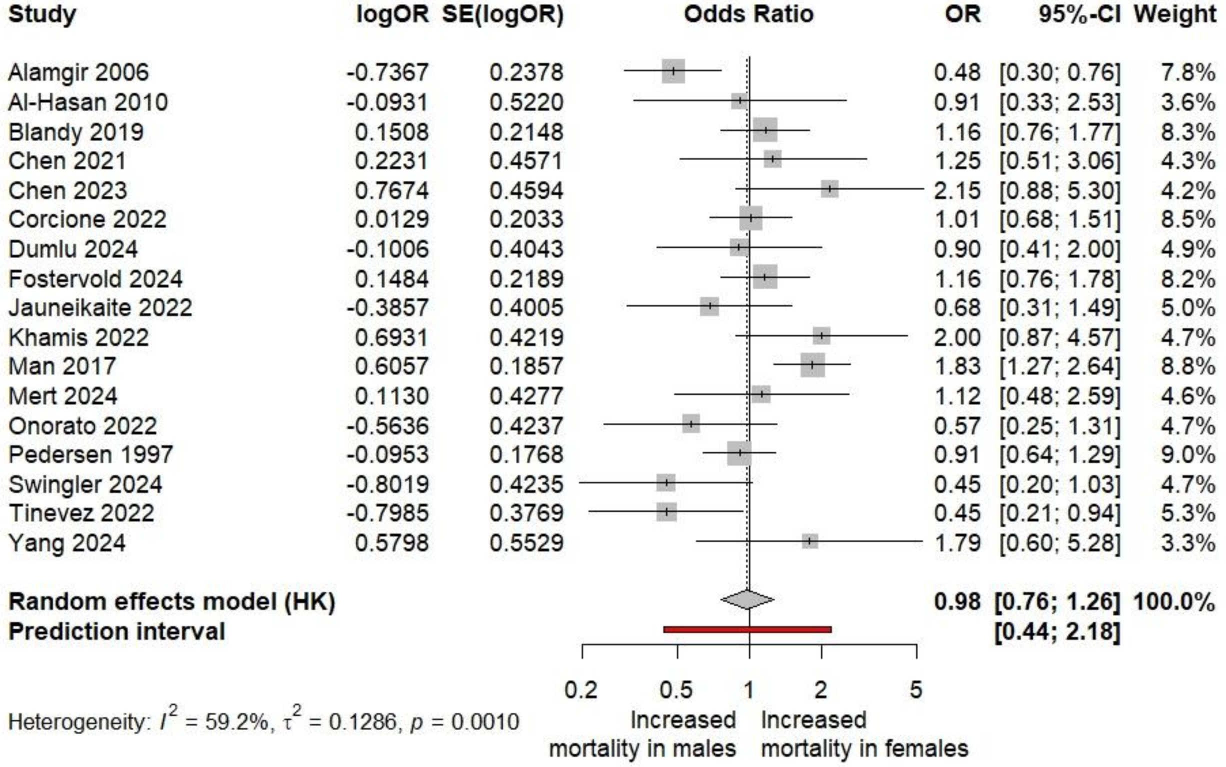

B.

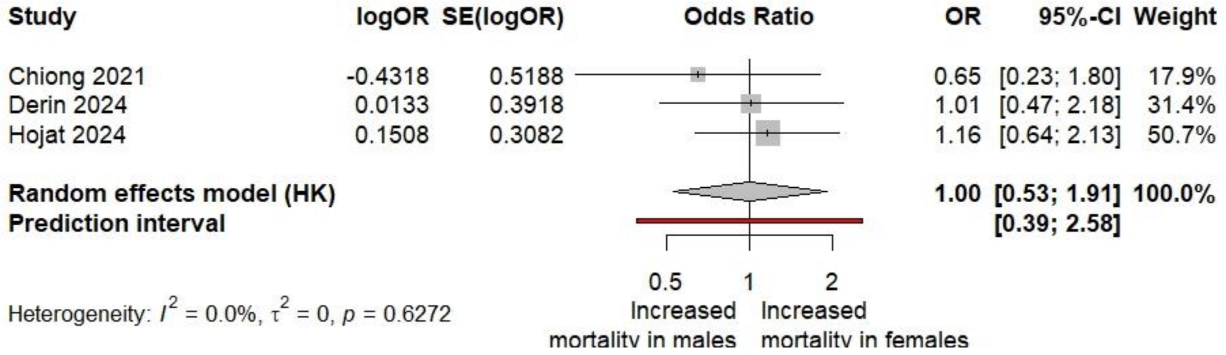

© 2025 La P et al. *JAMA Network Open*.

C.

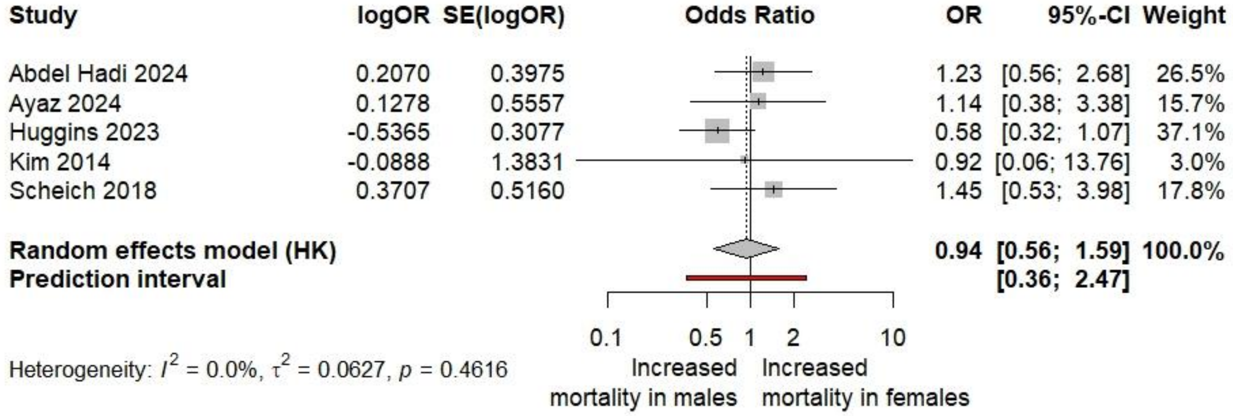

© 2025 La P et al. *JAMA Network Open*.

eAppendix 13. Subset meta-analyses, stratified by bacterial antibiotic resistance phenotype

Studies included in the primary analysis that did not restrict to a particular antibiotic resistance phenotype (A) or restricted to carbapenem-resistant gram-negative bacteria (B) are shown here. No association between biological sex and patient mortality was identified in these subsets.

A.

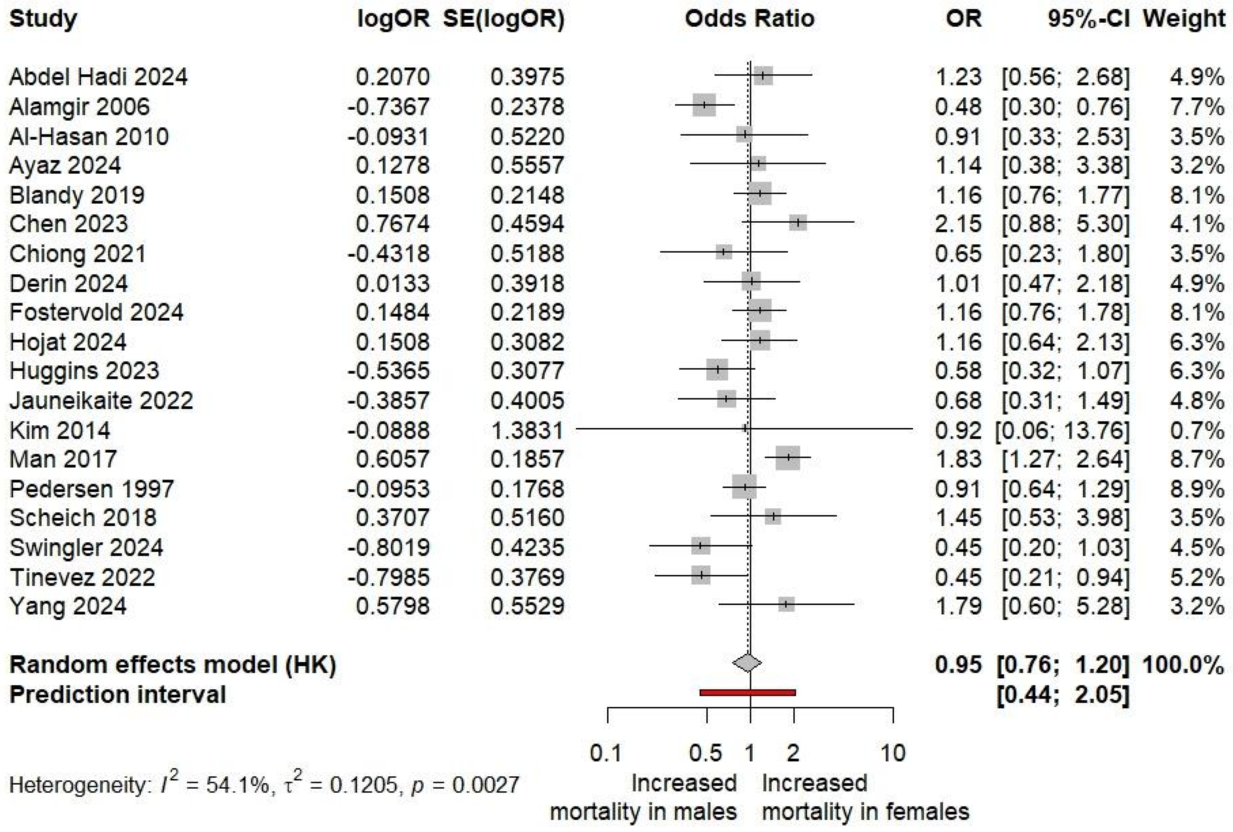

© 2025 La P et al. *JAMA Network Open*.

B.

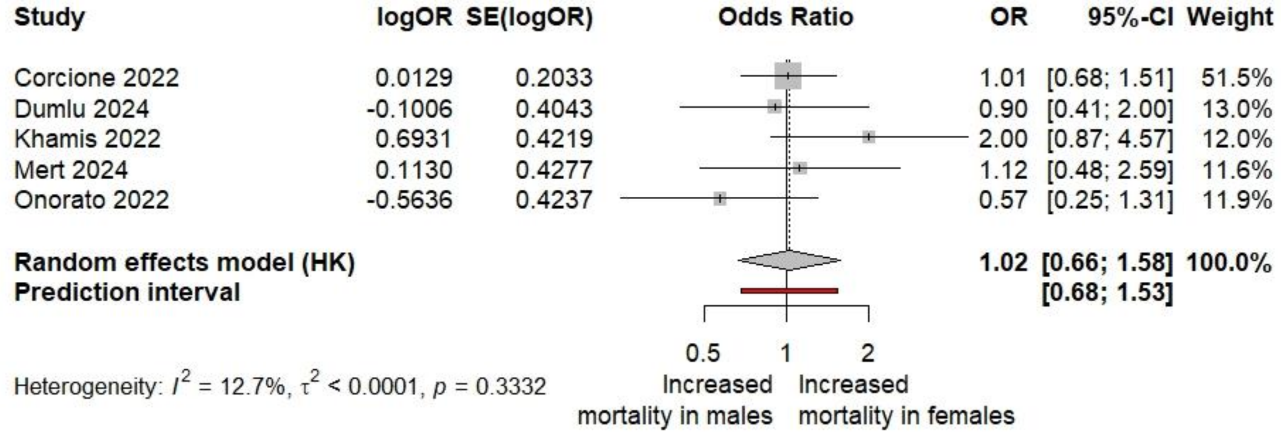

© 2025 La P et al. *JAMA Network Open*.

eAppendix 14. Subset meta-analyses, stratified by publication date

Meta-analyses of studies included in the primary analysis that were published prior to 2020 (A) or 2020 and later (B) are shown. No association between biological sex and patient mortality was identified in these subsets.

A.

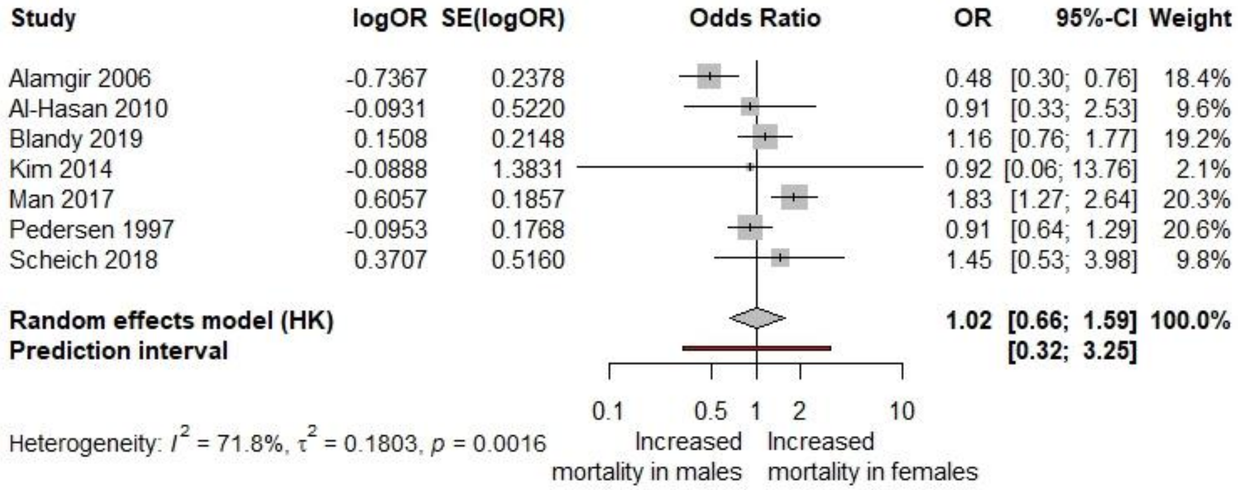

B.

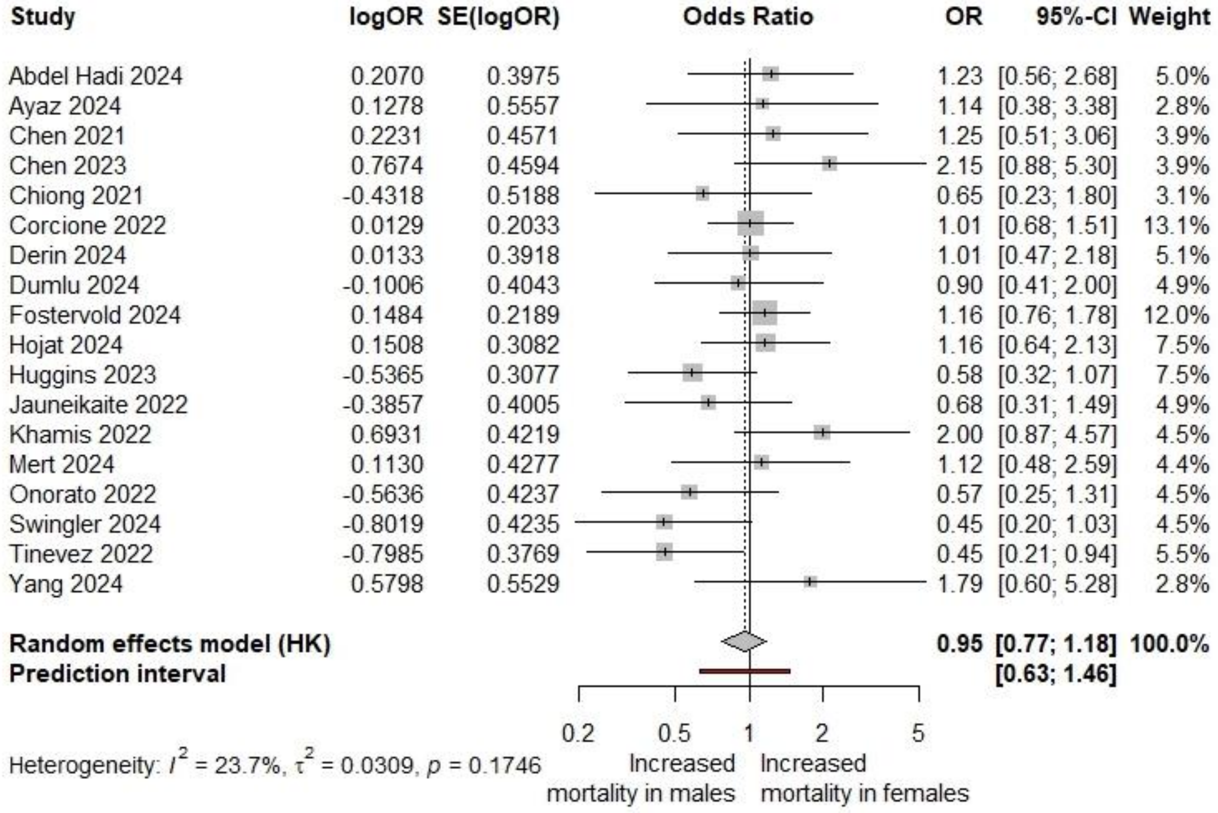

© 2025 La P et al. *JAMA Network Open*.

eAppendix 15. Secondary analysis of unadjusted sex-stratified mortality in patients with gram-negative bloodstream infection

In the 321 studies included in this meta-analysis,[8–328] female sex was associated with decreased mortality.

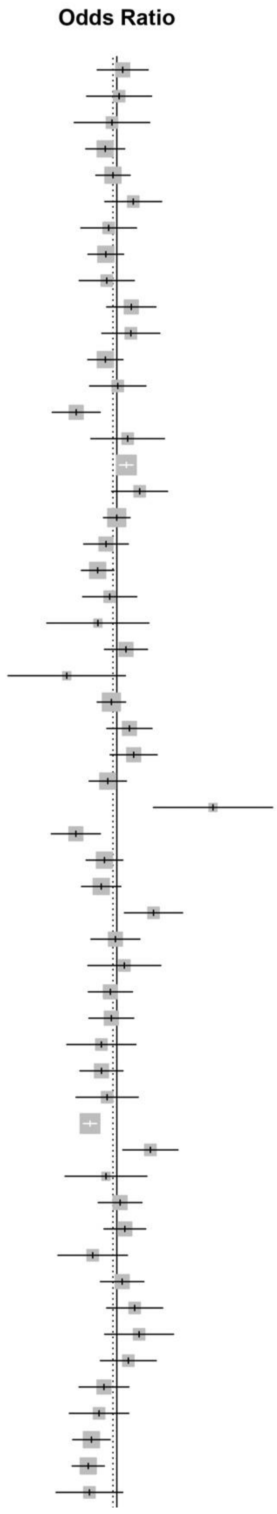

Study	logOR	SE(logOR)	OR	95%−CI	Weight
Ahn 2023	0.1607	0.3547	1.17	[0.59; 2.35]	0.3%
Al−Hasan 2010	0.0634	0.4523	1.07	[0.44; 2.59]	0.2%
Al−Hasan 2010	−0.1323	0.5249	0.88	[0.31; 2.45]	0.2%
Al−Hasan 2012	−0.3157	0.2713	0.73	[0.43; 1.24]	0.4%
Al−Hasan 2013	−0.1047	0.2389	0.90	[0.56; 1.44]	0.4%
Alves 2014	0.4485	0.3958	1.57	[0.72; 3.40]	0.2%
Amat 2018	−0.2220	0.3874	0.80	[0.37; 1.71]	0.2%
Amipara 2021	−0.2996	0.2480	0.74	[0.46; 1.20]	0.4%
Ang 2022	−0.2741	0.3836	0.76	[0.36; 1.61]	0.2%
Anggraini 2022	0.3948	0.3430	1.48	[0.76; 2.91]	0.3%
Arias Ramos 2023	0.3835	0.4036	1.47	[0.67; 3.24]	0.2%
Aryee 2020	−0.3125	0.2449	0.73	[0.45; 1.18]	0.4%
Aslan 2022	0.0261	0.3917	1.03	[0.48; 2.21]	0.2%
Atamna 2019	−1.1078	0.3336	0.33	[0.17; 0.64]	0.3%
Ayaz 2024	0.2959	0.5125	1.34	[0.49; 3.67]	0.2%
Babich 2020	0.2604	0.0947	1.30	[1.08; 1.56]	0.6%
Balkan 2015	0.6238	0.3886	1.87	[0.87; 4.00]	0.2%
Baltas 2021	−0.0018	0.1849	1.00	[0.69; 1.43]	0.5%
Bass 2015	−0.2948	0.3104	0.74	[0.41; 1.37]	0.3%
Battle 2019	−0.5232	0.2260	0.59	[0.38; 0.92]	0.4%
Bavaro 2023	−0.1942	0.3766	0.82	[0.39; 1.72]	0.2%
Benanti 2019	−0.5167	0.7103	0.60	[0.15; 2.40]	0.1%
Ben−David 2012	0.2500	0.2996	1.28	[0.71; 2.31]	0.3%
Biehle 2015	−1.3661	0.8166	0.26	[0.05; 1.26]	0.1%
Blandy 2019	−0.1477	0.1976	0.86	[0.59; 1.27]	0.5%
Boattini 2023	0.3443	0.3148	1.41	[0.76; 2.61]	0.3%
Boattini 2023	0.4598	0.3270	1.58	[0.83; 3.01]	0.3%
Bowers 2013	−0.2469	0.2596	0.78	[0.47; 1.30]	0.4%
Bozok 2024	2.6247	0.8290	13.80	[2.72; 70.07]	0.1%
Bryant 1971	−1.1177	0.3416	0.33	[0.17; 0.64]	0.3%
Burnham 2017	−0.3373	0.2555	0.71	[0.43; 1.18]	0.4%
Cain 2015	−0.4251	0.2739	0.65	[0.38; 1.12]	0.4%
Camins 2011	1.0014	0.4046	2.72	[1.23; 6.02]	0.2%
Caydasi 2024	−0.0381	0.3406	0.96	[0.49; 1.88]	0.3%
Ceken 2018	0.2057	0.5075	1.23	[0.45; 3.32]	0.2%
Cetin 2021	−0.1755	0.3073	0.84	[0.46; 1.53]	0.3%
Chang 2020	−0.1491	0.3120	0.86	[0.47; 1.59]	0.3%
Chapelet 2017	−0.4215	0.4807	0.66	[0.26; 1.68]	0.2%
Chaubey 2014	−0.4187	0.2998	0.66	[0.37; 1.18]	0.3%
Chen 2020	−0.2646	0.4333	0.77	[0.33; 1.79]	0.2%
Chen 2020	−0.7309	0.0931	0.48	[0.40; 0.58]	0.6%
Chen 2021	0.9163	0.3837	2.50	[1.18; 5.30]	0.2%
Chen 2021	−0.2955	0.5693	0.74	[0.24; 2.27]	0.1%
Chen 2021	0.0898	0.3044	1.09	[0.60; 1.99]	0.3%
Chen 2022	0.2188	0.2906	1.24	[0.70; 2.20]	0.3%
Chen 2022	−0.6581	0.4834	0.52	[0.20; 1.34]	0.2%
Chen 2022	0.1460	0.3024	1.16	[0.64; 2.09]	0.3%
Chen 2022	0.4871	0.3898	1.63	[0.76; 3.49]	0.2%
Chen 2023	0.6082	0.4789	1.84	[0.72; 4.70]	0.2%
Cheng 2016	0.3147	0.3886	1.37	[0.64; 2.93]	0.2%
Cheng 2017	−0.3507	0.3465	0.70	[0.36; 1.39]	0.3%
Cheng 2024	−0.4848	0.4134	0.62	[0.27; 1.38]	0.2%
Cheong 2007	−0.6926	0.2609	0.50	[0.30; 0.83]	0.4%
Cheong 2008	−0.7795	0.2241	0.46	[0.30; 0.71]	0.4%
Cheong 2008	−0.7472	0.4637	0.47	[0.19; 1.18]	0.2%

© 2025 La P et al. *JAMA Network Open*.

Cheong 2008	0.1090	0.4713	1.12	[0.44; 2.81]	0.2%
Cheong 2012	0.4898	0.4570	1.63	[0.67; 4.00]	0.2%
Chiang 2022	−0.2045	0.1647	0.82	[0.59; 1.13]	0.5%
Chiong 2021	−0.2222	0.4169	0.80	[0.35; 1.81]	0.2%
Cho 2015	−0.4855	0.5189	0.62	[0.22; 1.70]	0.2%
Choi 2019	−0.1372	0.4004	0.87	[0.40; 1.91]	0.2%
Chopra 2012	0.4840	0.3476	1.62	[0.82; 3.21]	0.3%
Chow 2015	0.2846	0.3603	1.33	[0.66; 2.69]	0.3%
Chuang 2011	0.0426	0.5135	1.04	[0.38; 2.85]	0.2%
Chuang 2011	0.1802	0.3578	1.20	[0.59; 2.41]	0.3%
Chung 2012	−0.0488	0.4204	0.95	[0.42; 2.17]	0.2%
Cooper 2024	−0.3608	0.0725	0.70	[0.60; 0.80]	0.7%
Corcione 2022	−0.1069	0.1953	0.90	[0.61; 1.32]	0.5%
Cristina 2018	−0.0488	0.3281	0.95	[0.50; 1.81]	0.3%
Dekic 2020	0.3259	0.3231	1.39	[0.74; 2.61]	0.3%
Delgado−Valverde 2016	−0.0210	0.4129	0.98	[0.44; 2.20]	0.2%
Derin 2024	−0.0462	0.3341	0.95	[0.50; 1.84]	0.3%
DeRosa 2011	1.5433	0.4903	4.68	[1.79; 12.23]	0.2%
Drozdinsky 2021	0.1813	0.3374	1.20	[0.62; 2.32]	0.3%
Dumlu 2024	0.1992	0.3349	1.22	[0.63; 2.35]	0.3%
Durdu 2016	−0.1231	0.2967	0.88	[0.49; 1.58]	0.3%
Erbay 2009	0.2070	0.4074	1.23	[0.55; 2.73]	0.2%
Ergonul 2016	0.3281	0.1404	1.39	[1.05; 1.83]	0.6%
Escrihuela−Vidal 2024	0.0144	0.2783	1.01	[0.59; 1.75]	0.4%
Falcone 2016	−0.0146	0.4248	0.99	[0.43; 2.27]	0.2%
Falcone 2020	0.5680	0.4158	1.76	[0.78; 3.99]	0.2%
Falcone 2023	0.3435	0.1368	1.41	[1.08; 1.84]	0.6%
Fang 2023	−0.5113	0.3534	0.60	[0.30; 1.20]	0.3%
Fatima 2023	0.1697	0.3531	1.18	[0.59; 2.37]	0.3%
Ferreira 2021	−0.1849	0.7226	0.83	[0.20; 3.43]	0.1%
Fitzpatrick 2016	−0.4986	0.3023	0.61	[0.34; 1.10]	0.3%
Florez Riano 2024	−0.1271	0.4662	0.88	[0.35; 2.20]	0.2%
Fostervold 2024	−0.0303	0.2145	0.97	[0.64; 1.48]	0.4%
Fraenkel−Wandel 2016	0.9111	0.2890	2.49	[1.41; 4.38]	0.3%
Fu 2015	0.0540	0.3674	1.06	[0.51; 2.17]	0.3%
Fuentes−Gonzalez 2024	1.0788	0.4206	2.94	[1.29; 6.71]	0.2%
Galofre 1994	−0.3064	0.5424	0.74	[0.25; 2.13]	0.1%
Gao 2022	0.1931	0.2176	1.21	[0.79; 1.86]	0.4%
Gezer 2024	−0.1491	0.2316	0.86	[0.55; 1.36]	0.4%
Giannella 2018	−0.5586	0.3082	0.57	[0.31; 1.05]	0.3%
Giannella 2018	0.0473	0.2050	1.05	[0.70; 1.57]	0.5%
Giannella 2019	−0.1287	0.1673	0.88	[0.63; 1.22]	0.5%
Giannella 2019	−0.1181	0.2764	0.89	[0.52; 1.53]	0.4%
Giannella 2020	−0.1287	0.1673	0.88	[0.63; 1.22]	0.5%
Girometti 2014	−0.5175	0.3358	0.60	[0.31; 1.15]	0.3%
Gomez−Simmonds 2015	−0.0246	0.3160	0.98	[0.53; 1.81]	0.3%
Gomez−Simmonds 2016	0.4102	0.3653	1.51	[0.74; 3.08]	0.3%
Gozel 2012	−0.0931	0.2887	0.91	[0.52; 1.60]	0.3%
Gradel 2006	−0.0311	0.6198	0.97	[0.29; 3.27]	0.1%
Gu 2016	0.0583	0.4109	1.06	[0.47; 2.37]	0.2%
Gutierrez−Gutierrez 2016	−0.1740	0.2459	0.84	[0.52; 1.36]	0.4%
Gutierrez−Gutierrez 2016	−0.1193	0.2587	0.89	[0.53; 1.47]	0.4%
Gutierrez−Gutierrez 2016	−0.2776	0.3191	0.76	[0.41; 1.42]	0.3%
Hazwan 2022	0.3914	0.3859	1.48	[0.69; 3.15]	0.2%

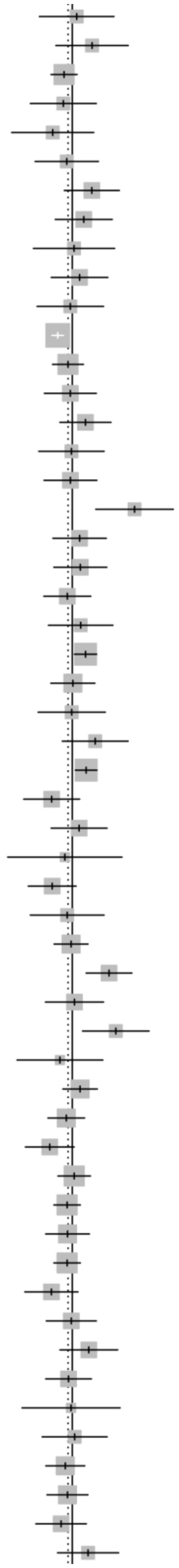

© 2025 La P et al. *JAMA Network Open*.

Herrera 2023	−0.1508	0.1831	0.86	[0.60; 1.23]	0.5%
Hirsch 2012	0.0672	0.4674	1.07	[0.43; 2.67]	0.2%
Hodgin 1965	−0.6176	0.4081	0.54	[0.24; 1.20]	0.2%
Hojat 2024	0.0031	0.1723	1.00	[0.72; 1.41]	0.5%
Hou 2023	0.2914	0.3685	1.34	[0.65; 2.76]	0.3%
Hsieh 2016	−0.2670	0.3496	0.77	[0.39; 1.52]	0.3%
Hsu 2021	−0.1655	0.4686	0.85	[0.34; 2.12]	0.2%
Huang 2015	−0.2518	0.3176	0.78	[0.42; 1.45]	0.3%
Huang 2024	0.1564	0.3027	1.17	[0.65; 2.12]	0.3%
Huang 2025	0.6745	0.4015	1.96	[0.89; 4.31]	0.2%
Huh 2014	−0.3702	0.3799	0.69	[0.33; 1.45]	0.2%
Huh 2020	−0.2915	0.1471	0.75	[0.56; 1.00]	0.6%
Imai 2019	0.7577	0.6498	2.13	[0.60; 7.62]	0.1%
Ioannou 2023	−0.0227	0.2214	0.98	[0.63; 1.51]	0.4%
Isler 2022	−0.4023	0.3685	0.67	[0.32; 1.38]	0.3%
Iwasaki 2025	−0.2064	0.4240	0.81	[0.35; 1.87]	0.2%
Jeon 2021	−0.1166	0.3252	0.89	[0.47; 1.68]	0.3%
Jian 2022	−0.3398	0.2926	0.71	[0.40; 1.26]	0.3%
Joo 2017	1.0112	0.5813	2.75	[0.88; 8.59]	0.1%
Kaki 2024	−0.0153	0.4140	0.98	[0.44; 2.22]	0.2%
Kalam 2014	−0.2624	0.2696	0.77	[0.45; 1.30]	0.4%
Kanchanasuwan 2022	0.4283	0.4212	1.53	[0.67; 3.50]	0.2%
Kang 2004	0.0465	0.4153	1.05	[0.46; 2.36]	0.2%
Kang 2011	−0.5886	0.1422	0.56	[0.42; 0.73]	0.6%
Kang 2020	−0.2329	0.2519	0.79	[0.48; 1.30]	0.4%
Karaiskos 2021	0.4616	0.3827	1.59	[0.75; 3.36]	0.2%
Khamis 2022	0.2390	0.3184	1.27	[0.68; 2.37]	0.3%
Kim 2014	−0.9051	0.5329	0.40	[0.14; 1.15]	0.1%
Kim 2014	−0.2615	0.3183	0.77	[0.41; 1.44]	0.3%
Kim 2019	−0.6287	0.2396	0.53	[0.33; 0.85]	0.4%
Kim 2019	−0.1528	0.2424	0.86	[0.53; 1.38]	0.4%
Kim 2023	−0.0404	0.2648	0.96	[0.57; 1.61]	0.4%
Kim 2023	−0.3185	0.4231	0.73	[0.32; 1.67]	0.2%
Kim 2024	0.0618	0.2909	1.06	[0.60; 1.88]	0.3%
Ko 2000	−0.4336	0.5634	0.65	[0.21; 1.96]	0.1%
Komatsu 2018	−0.5008	0.6744	0.61	[0.16; 2.27]	0.1%
Kong 2022	0.6604	0.3591	1.94	[0.96; 3.91]	0.3%
Ku 2012	−0.7662	0.4462	0.46	[0.19; 1.11]	0.2%
Ku 2014	−0.1858	0.3374	0.83	[0.43; 1.61]	0.3%
Kuikka 1997	−0.7877	0.2982	0.45	[0.25; 0.82]	0.3%
Kuo 2013	−0.0513	0.4675	0.95	[0.38; 2.37]	0.2%
Kuo 2017	−0.9398	0.2030	0.39	[0.26; 0.58]	0.5%
Kurt 2024	−0.0074	0.2910	0.99	[0.56; 1.76]	0.3%
Lay 2010	−0.5518	0.3851	0.58	[0.27; 1.23]	0.2%
Leao 2016	−0.6615	0.4314	0.52	[0.22; 1.20]	0.2%
Lee 2010	−0.6382	0.3278	0.53	[0.28; 1.00]	0.3%
Lee 2012	−0.2482	0.3172	0.78	[0.42; 1.45]	0.3%
Lee 2013	−0.0584	0.3726	0.94	[0.45; 1.96]	0.2%
Lee 2015	−0.0596	0.3468	0.94	[0.48; 1.86]	0.3%
Lee 2017	−0.8091	0.4206	0.45	[0.20; 1.02]	0.2%
Lee 2018	−0.9577	0.1999	0.38	[0.26; 0.57]	0.5%
Lee 2018	−0.8492	0.4193	0.43	[0.19; 0.97]	0.2%
Lee 2019	−1.0485	0.2904	0.35	[0.20; 0.62]	0.3%
Lee 2019	−1.2921	0.5568	0.27	[0.09; 0.82]	0.1%
Lee 2021	−0.1602	0.4356	0.85	[0.36; 2.00]	0.2%
Lee 2021	0.2147	0.4599	1.24	[0.50; 3.05]	0.2%

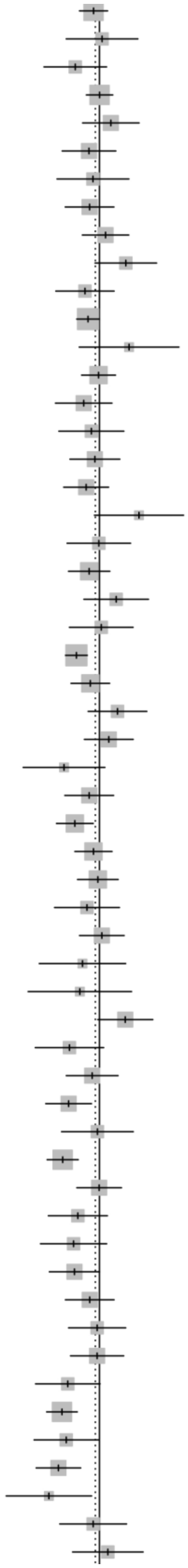

© 2025 La P et al. *JAMA Network Open*.

Lee 2022	−0.3007	0.2965	0.74	[0.41; 1.32]	0.3%
Lee 2022	−0.3412	0.2915	0.71	[0.40; 1.26]	0.3%
Lee 2023	−0.2875	0.2901	0.75	[0.42; 1.32]	0.3%
Lefort 2011	−0.4824	0.1845	0.62	[0.43; 0.89]	0.5%
Li 2012	−0.0597	0.3259	0.94	[0.50; 1.78]	0.3%
Li 2017	−0.8938	0.5188	0.41	[0.15; 1.13]	0.2%
Li 2017	−0.1054	0.3767	0.90	[0.43; 1.88]	0.2%
Li 2018	−0.9799	0.5848	0.38	[0.12; 1.18]	0.1%
Li 2018	0.2211	0.3635	1.25	[0.61; 2.54]	0.3%
Li 2020	0.1389	0.2141	1.15	[0.76; 1.75]	0.4%
Li 2023	−0.4071	0.3963	0.67	[0.31; 1.45]	0.2%
Li 2024	0.3906	0.2565	1.48	[0.89; 2.44]	0.4%
Li 2024	0.3610	0.4904	1.43	[0.55; 3.75]	0.2%
Li 2024	−0.3209	0.3363	0.73	[0.38; 1.40]	0.3%
Li 2024	−0.3458	0.4252	0.71	[0.31; 1.63]	0.2%
Liang 2022	0.1273	0.4771	1.14	[0.45; 2.89]	0.2%
Lim 2019	−0.5339	0.5055	0.59	[0.22; 1.58]	0.2%
Lin 2021	−0.0306	0.2536	0.97	[0.59; 1.59]	0.4%
Lin 2021	0.3846	0.3588	1.47	[0.73; 2.97]	0.3%
Lin 2022	−0.1823	0.6194	0.83	[0.25; 2.81]	0.1%
Lines 2019	−0.5950	0.2244	0.55	[0.36; 0.86]	0.4%
Liu 2020	−0.2451	0.3124	0.78	[0.42; 1.44]	0.3%
Liu 2024	0.3673	0.5067	1.44	[0.53; 3.90]	0.2%
Lodise 2007	0.1978	0.4338	1.22	[0.52; 2.85]	0.2%
Lye 2012	−0.5010	0.1895	0.61	[0.42; 0.88]	0.5%
Machuca 2017	−0.0843	0.4277	0.92	[0.40; 2.13]	0.2%
Maldonado 2024	−0.1244	0.2974	0.88	[0.49; 1.58]	0.3%
Man 2017	0.2862	0.1703	1.33	[0.95; 1.86]	0.5%
Man 2021	0.0975	0.1378	1.10	[0.84; 1.44]	0.6%
Manesh 2023	−0.0966	0.3356	0.91	[0.47; 1.75]	0.3%
Marcos 2008	−0.2647	0.3529	0.77	[0.38; 1.53]	0.3%
Martinez 2010	−0.2898	0.0983	0.75	[0.62; 0.91]	0.6%
McCowan 2022	−0.3694	0.0531	0.69	[0.62; 0.77]	0.7%
Meng 2021	0.3918	0.3081	1.48	[0.81; 2.71]	0.3%
Meng 2022	0.1178	0.4757	1.12	[0.44; 2.86]	0.2%
Mert 2024	0.0829	0.4229	1.09	[0.47; 2.49]	0.2%
Metan 2009	−0.5153	0.4175	0.60	[0.26; 1.35]	0.2%
Metan 2013	−0.2113	0.4141	0.81	[0.36; 1.82]	0.2%
Micek 2011	−0.2014	0.1753	0.82	[0.58; 1.15]	0.5%
Mitsuboshi 2020	−0.9564	0.4982	0.38	[0.14; 1.02]	0.2%
Montero 2020	−0.2021	0.2776	0.82	[0.47; 1.41]	0.4%
Montero 2020	−0.3641	0.2571	0.69	[0.42; 1.15]	0.4%
Mora−Rillo 2015	−0.9740	0.4287	0.38	[0.16; 0.87]	0.2%
Morata 2012	−0.0211	0.1995	0.98	[0.66; 1.45]	0.5%
Nakanishi 2023	−0.3194	0.2879	0.73	[0.41; 1.28]	0.3%
Namikawa 2019	0.1777	0.6420	1.19	[0.34; 4.20]	0.1%
Nasir 2019	−0.3905	0.2869	0.68	[0.39; 1.19]	0.3%
Nasomsong 2022	0.1434	0.4822	1.15	[0.45; 2.97]	0.2%
Naylor 2019	−0.2343	0.0481	0.79	[0.72; 0.87]	0.7%
Nelson 2015	0.2801	0.3416	1.32	[0.68; 2.58]	0.3%
Ngoma 2024	0.2042	0.1077	1.23	[0.99; 1.51]	0.6%
Nham 2020	0.2268	0.3281	1.25	[0.66; 2.39]	0.3%
Ni 2022	−0.7408	0.2406	0.48	[0.30; 0.76]	0.4%
Niu 2018	0.4707	0.2868	1.60	[0.91; 2.81]	0.3%
Niu 2019	−0.6901	0.5169	0.50	[0.18; 1.38]	0.2%
Niu 2019	0.2268	0.2656	1.25	[0.75; 2.11]	0.4%

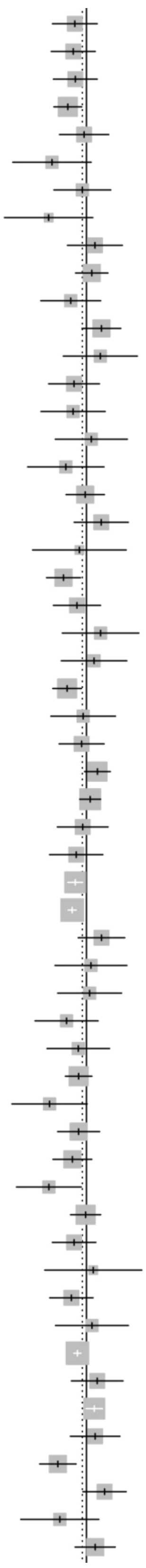

© 2025 La P et al. *JAMA Network Open*.

Oliva 2022	0.1840	0.5155	1.20	[0.44; 3.30]	0.2%
Onorato 2022	−0.7358	0.3786	0.48	[0.23; 1.01]	0.2%
Osih 2007	−0.6598	0.3580	0.52	[0.26; 1.04]	0.3%
Ozdede 2024	−0.1778	0.3637	0.84	[0.41; 1.71]	0.3%
Palacios−Baena 2017	0.0549	0.2080	1.06	[0.70; 1.59]	0.5%
Palacios−Baena 2019	−0.0462	0.3815	0.95	[0.45; 2.02]	0.2%
Papadimitriou−Olivgeris 2017	0.0223	0.4147	1.02	[0.45; 2.31]	0.2%
Papadimitriou−Olivgeris 2021	0.5336	0.3989	1.71	[0.78; 3.73]	0.2%
Park 2011	−0.9510	0.5250	0.39	[0.14; 1.08]	0.2%
Park 2012	0.7340	0.6458	2.08	[0.59; 7.39]	0.1%
Park 2013	0.1178	0.3666	1.12	[0.55; 2.31]	0.3%
Park 2013	−0.2136	0.3830	0.81	[0.38; 1.71]	0.2%
Park 2018	−0.3876	0.3701	0.68	[0.33; 1.40]	0.3%
Park 2019	0.0953	0.3683	1.10	[0.53; 2.26]	0.3%
Park 2022	−0.5611	0.3365	0.57	[0.30; 1.10]	0.3%
Parkins 2010	0.4055	0.2756	1.50	[0.87; 2.57]	0.4%
Pascale 2021	0.1447	0.2905	1.16	[0.65; 2.04]	0.3%
Pedersen 1997	−0.1986	0.1641	0.82	[0.59; 1.13]	0.5%
Pena 2012	0.0956	0.1878	1.10	[0.76; 1.59]	0.5%
Pena 2015	−0.3002	0.2856	0.74	[0.42; 1.30]	0.3%
Peralta 2007	−0.1062	0.3839	0.90	[0.42; 1.91]	0.2%
Peralta 2010	0.0825	0.2617	1.09	[0.65; 1.81]	0.4%
Peralta 2012	−0.1274	0.2723	0.88	[0.52; 1.50]	0.4%
Perez−Nadales 2020	−0.0770	0.3314	0.93	[0.48; 1.77]	0.3%
Perez−Nadales 2023	0.8140	0.3997	2.26	[1.03; 4.94]	0.2%
Persoon 2020	−0.0946	0.3144	0.91	[0.49; 1.68]	0.3%
Phe 2019	0.0123	0.5038	1.01	[0.38; 2.72]	0.2%
Phungoen 2022	0.5632	0.4184	1.76	[0.77; 3.99]	0.2%
Poon 2012	−0.7655	0.5300	0.47	[0.16; 1.31]	0.1%
Qian 2023	−0.2419	0.3346	0.79	[0.41; 1.51]	0.3%
Quillici 2021	0.3320	0.2711	1.39	[0.82; 2.37]	0.4%
Qureshi 2012	0.0000	0.2450	1.00	[0.62; 1.62]	0.4%
Ramanathan 2022	0.0250	0.2661	1.03	[0.61; 1.73]	0.4%
Recio 2020	0.2902	0.3388	1.34	[0.69; 2.60]	0.3%
Reese 2024	−0.3886	0.2304	0.68	[0.43; 1.06]	0.4%
Rhodes 2015	0.0315	0.4343	1.03	[0.44; 2.42]	0.2%
Rigatto 2022	0.4030	0.2471	1.50	[0.92; 2.43]	0.4%
Rodriguez−Bano 2010	−0.0232	0.3810	0.98	[0.46; 2.06]	0.2%
Rodriguez−Bano 2012	−0.1975	0.4450	0.82	[0.34; 1.96]	0.2%
Rodriguez−Bano 2013	0.1518	0.3373	1.16	[0.60; 2.25]	0.3%
Rolo 2022	0.4355	0.2456	1.55	[0.96; 2.50]	0.4%
Russo 2019	0.9877	0.3272	2.68	[1.41; 5.10]	0.3%
Sangsuwan 2021	0.1359	0.2233	1.15	[0.74; 1.77]	0.4%
Sathya Kumar 2023	−0.2810	0.3296	0.76	[0.40; 1.44]	0.3%
Scheich 2018	−0.1137	0.4863	0.89	[0.34; 2.31]	0.2%
Scheuerman 2018	−0.0966	0.2022	0.91	[0.61; 1.35]	0.5%
Scheuerman 2018	0.1066	0.2892	1.11	[0.63; 1.96]	0.3%

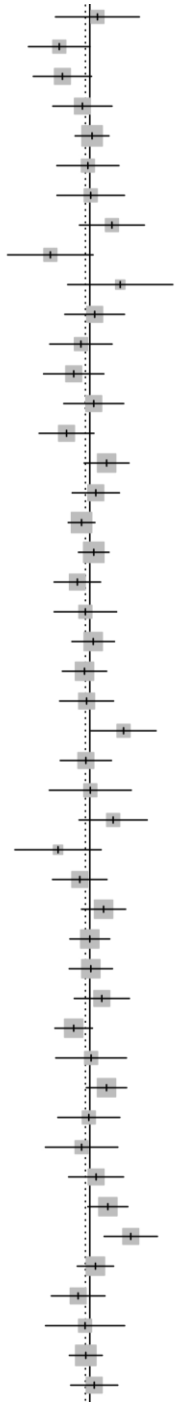

© 2025 La P et al. *JAMA Network Open*.

Schwaber 2006	0.1784	0.3223	1.20 [0.64; 2.25]	0.3%
Seo 2020	−0.2647	0.4831	0.77 [0.30; 1.98]	0.2%
Shalabi 2024	0.2324	0.5543	1.26 [0.43; 3.74]	0.1%
Shi 2019	0.0148	0.2586	1.01 [0.61; 1.68]	0.4%
Shi 2022	−0.2056	0.2047	0.81 [0.55; 1.22]	0.5%
Siedner 2014	0.5325	0.3016	1.70 [0.94; 3.08]	0.3%
Son 2020	0.3311	0.3391	1.39 [0.72; 2.71]	0.3%
Suh 2024	−0.1335	0.5101	0.88 [0.32; 2.38]	0.2%
Swingler 2024	−0.6678	0.3126	0.51 [0.28; 0.95]	0.3%
Szilagyi 2009	−0.5082	0.3466	0.60 [0.31; 1.19]	0.3%
Tam 2010	−0.5988	0.5326	0.55 [0.19; 1.56]	0.1%
Tang 2017	−0.3467	0.3690	0.71 [0.34; 1.46]	0.3%
Tang 2021	−0.1033	0.2101	0.90 [0.60; 1.36]	0.5%
Tinevez 2022	−0.6539	0.3182	0.52 [0.28; 0.97]	0.3%
Tiseo 2024	0.4374	0.3007	1.55 [0.86; 2.79]	0.3%
Trecarichi 2016	−0.1975	0.2513	0.82 [0.50; 1.34]	0.4%
Trecarichi 2019	−1.0821	0.5152	0.34 [0.12; 0.93]	0.2%
Tu 2018	0.0500	0.4018	1.05 [0.48; 2.31]	0.2%
Tumbarello 2007	0.2644	0.3047	1.30 [0.72; 2.37]	0.3%
Tumbarello 2008	0.4285	0.3899	1.53 [0.71; 3.30]	0.2%
Tumbarello 2011	−0.3410	0.4223	0.71 [0.31; 1.63]	0.2%
Tumbarello 2012	−0.2222	0.3700	0.80 [0.39; 1.65]	0.3%
Vieceli 2024	−0.2475	0.4100	0.78 [0.35; 1.74]	0.2%
Wang 2018	−0.7340	0.2620	0.48 [0.29; 0.80]	0.4%
Wang 2019	0.0690	0.3539	1.07 [0.54; 2.14]	0.3%
Wang 2022	−0.0238	0.2187	0.98 [0.64; 1.50]	0.4%
Wang 2024	−0.6261	0.1687	0.53 [0.38; 0.74]	0.5%
Wang 2024	−1.0986	0.3670	0.33 [0.16; 0.68]	0.3%
Wang 2024	−0.5213	0.4058	0.59 [0.27; 1.32]	0.2%
Watanakunakorn 1994	−0.2364	0.4001	0.79 [0.36; 1.73]	0.2%
Wei 2022	0.3272	0.4082	1.39 [0.62; 3.09]	0.2%
Wei 2024	−0.2059	0.3725	0.81 [0.39; 1.69]	0.2%
Wen 2024	−0.2006	0.3822	0.82 [0.39; 1.73]	0.2%
Willmann 2013	−0.0250	0.4024	0.98 [0.44; 2.15]	0.2%
Wu 2012	−1.9063	0.2727	0.15 [0.09; 0.25]	0.4%
Wu 2018	−0.5070	0.5222	0.60 [0.22; 1.68]	0.2%
Wu 2022	0.4388	0.3770	1.55 [0.74; 3.25]	0.2%
Xiao 2018	−0.1901	0.2850	0.83 [0.47; 1.45]	0.3%
Xiao 2019	0.0527	0.3708	1.05 [0.51; 2.18]	0.2%
Xiao 2019	−0.0739	0.2578	0.93 [0.56; 1.54]	0.4%
Xiao 2020	−0.5449	0.2781	0.58 [0.34; 1.00]	0.4%
Xu 2018	−0.2576	0.3415	0.77 [0.40; 1.51]	0.3%
Xu 2022	0.1733	0.3569	1.19 [0.59; 2.39]	0.3%
Xu 2022	1.0224	0.2913	2.78 [1.57; 4.92]	0.3%
Xu 2023	0.3350	0.3434	1.40 [0.71; 2.74]	0.3%
Xu 2023	−0.0296	0.0663	0.97 [0.85; 1.11]	0.7%
Xu 2024	−0.4281	0.4592	0.65 [0.26; 1.60]	0.2%
Yang 2018	−0.0606	0.4819	0.94 [0.37; 2.42]	0.2%
Yang 2022	−0.4955	0.3800	0.61 [0.29; 1.28]	0.2%
Yang 2024	0.1077	0.3325	1.11 [0.58; 2.14]	0.3%
Yang 2024	0.4780	0.4454	1.61 [0.67; 3.86]	0.2%
Yildiz 2023	0.1502	0.1956	1.16 [0.79; 1.70]	0.5%
Yoon 2017	−0.0412	0.2753	0.96 [0.56; 1.65]	0.4%
Yoon 2019	0.1265	0.6048	1.13 [0.35; 3.71]	0.1%
You 2024	−0.3498	0.4763	0.70 [0.28; 1.79]	0.2%
Yu 2024	0.2531	0.4617	1.29 [0.52; 3.18]	0.2%

© 2025 La P et al. *JAMA Network Open*.

Yuan 2023	0.4089	0.3782	1.51	[0.72; 3.16]	0.2%
Yuan 2024	0.0457	0.2442	1.05	[0.65; 1.69]	0.4%
Zhang 2017	0.1722	0.2938	1.19	[0.67; 2.11]	0.3%
Zhang 2019	0.4527	0.2735	1.57	[0.92; 2.69]	0.4%
Zhang 2020	−0.3608	0.2598	0.70	[0.42; 1.16]	0.4%
Zhang 2020	0.0238	0.2513	1.02	[0.63; 1.68]	0.4%
Zhang 2022	−0.1537	0.2675	0.86	[0.51; 1.45]	0.4%
Zhang 2024	−0.2983	0.4389	0.74	[0.31; 1.75]	0.2%
Zhao 2020	−0.9146	0.4619	0.40	[0.16; 0.99]	0.2%
Zhao 2022	−0.1886	0.3009	0.83	[0.46; 1.49]	0.3%
Zhao 2023	−0.6343	0.2978	0.53	[0.30; 0.95]	0.3%
Zhou 2019	0.1956	0.2658	1.22	[0.72; 2.05]	0.4%
Zhou 2021	0.4508	0.3034	1.57	[0.87; 2.84]	0.3%
Zhou 2024	−0.1544	0.2934	0.86	[0.48; 1.52]	0.3%
Zou 2021	−0.6887	0.3059	0.50	[0.28; 0.91]	0.3%
**Random effects model (HK)**			**0.90**	**[0.86; 0.94]**	**100.0%**

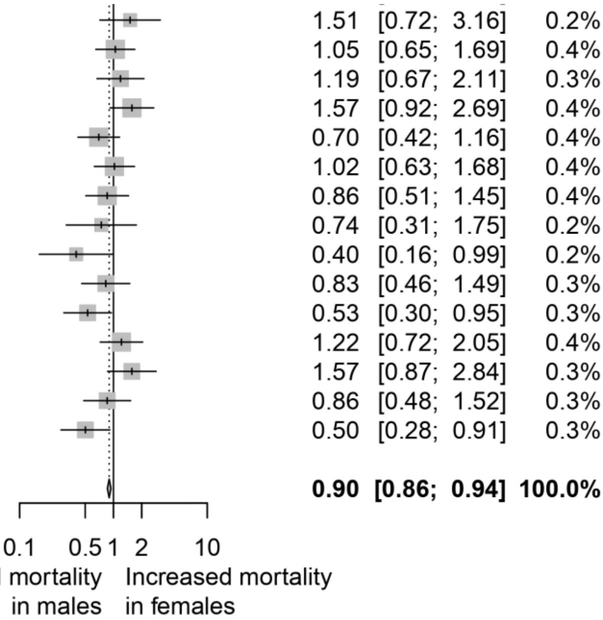

Heterogeneity: $I^2 = 51.0\%$, $\tau^2 = 0.0718$, $p < 0.0001$

© 2025 La P et al. *JAMA Network Open*.

eAppendix 16. Funnel plot of studies included in the secondary analysis

Publication bias was indicated by the Egger's regression test (p = 0.003).

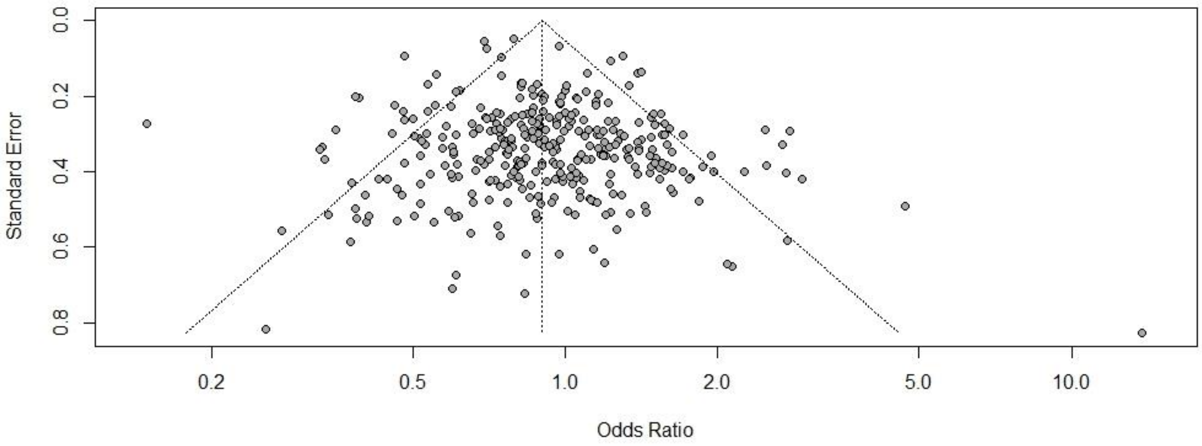

eReferences.

1. Berkman ND, Lohr KN, Ansari MT, et al. Grading the strength of a body of evidence when assessing health care interventions: an EPC update. *J Clin Epidemiol*. 2015;68(11):1312-1324. doi:10.1016/j.jclinepi.2014.11.023

2. Maskarinec SA, Park LP, Ruffin F, et al. Positive follow-up blood cultures identify high mortality risk among patients with Gram-negative bacteraemia. *Clin Microbiol Infect*. 2020;26(7):904-910. doi:10.1016/j.cmi.2020.01.025

3. Al-Hasan MN, Juhn YJ, Bang DW, Yang HJ, Baddour LM. External validation of bloodstream infection mortality risk score in a population-based cohort. *Clin Microbiol Infect Off Publ Eur Soc Clin Microbiol Infect Dis*. 2014;20(9):886-891. doi:10.1111/1469-0691.12607

4. Mischnik A, Baltus H, Walker SV, et al. Gram-negative bloodstream infections in six German university hospitals, 2016-2020: clinical and microbiological features. *Infection*. 2025;53(2):625-633. doi:10.1007/s15010-024-02430-7

5. Charlson ME, Pompei P, Ales KL, MacKenzie CR. A new method of classifying prognostic comorbidity in longitudinal studies: Development and validation. *J Chronic Dis*. 1987;40(5):373-383. doi:10.1016/0021-9681(87)90171-8

6. Elixhauser A, Steiner C, Harris DR, Coffey RM. Comorbidity measures for use with administrative data. *Med Care*. 1998;36(1):8-27. doi:10.1097/00005650-199801000-00004

7. McCABE WR. Gram-Negative Bacteremia: I. Etiology and Ecology. *Arch Intern Med*. 1962;110(6):847. doi:10.1001/archinte.1962.03620240029006

8. Ahn JY, Ahn SM, Kim JH, et al. Clinical Characteristics and Associated Factors for Mortality in Patients with Carbapenem-Resistant Enterobacteriaceae Bloodstream Infection. *Microorganisms*. 2023;11(5):25. doi:10.3390/microorganisms11051121

9. Al-Hasan MN, Eckel-Passow JE, Baddour LM. Impact of healthcare-associated acquisition on community-onset Gram-negative bloodstream infection: A population-based study. *Eur J Clin Microbiol Infect Dis*. 2012;31(6):1163-1171. doi:10.1007/s10096-011-1424-6

10. Al-Hasan MN, Lahr BD, Eckel-Passow JE, Baddour LM. Epidemiology and outcome of Klebsiella species bloodstream infection: a population-based study. *Mayo Clin Proc*. 2010;85(2):139-144. doi:10.4065/mcp.2009.0410

11. Alves MD, Ribeiro VB, Tessari JP, et al. Effect of cefepime dose on mortality of patients with Gram-negative bacterial bloodstream infections: a prospective cohort study. *J Antimicrob Chemother*. 2014;69(6):1681-1687. doi:10.1093/jac/dku001

12. Amat T, Gutierrez-Pizarraya A, Machuca I, et al. The combined use of tigecycline with high-dose colistin might not be associated with higher survival in critically ill patients with bacteraemia due to carbapenem-resistant Acinetobacter baumannii. *Clin Microbiol Infect*. 2018;24(6):630-634. doi:10.1016/j.cmi.2017.09.016

13. Amipara R, Winders HR, Justo JA, Bookstaver PB, Kohn J, Al-Hasan MN. Impact of follow up blood cultures on outcomes of patients with community-onset gram-negative

© 2025 La P et al. *JAMA Network Open*.

bloodstream infection. *eClinicalMedicine*. 2021;34:100811. doi:10.1016/j.eclinm.2021.100811

14. Ang SH, Periyasamy P, Shah SA, Ramli R, Kori N, Lau CL. Risk factors for complications and survival outcomes of Klebsiella pneumoniae Bacteraemia in Hospital Canselor Tuanku Muhriz Universiti Kebangsaan Malaysia. *Med J Malaysia*. 2022;77(4):440-445.

15. Anggraini D, Santosaningsih D, Endraswari PD, et al. Multicenter Study of the Risk Factors and Outcomes of Bloodstream Infections Caused by Carbapenem-Non-Susceptible Acinetobacter baumannii in Indonesia. *Trop Med Infect Dis*. 2022;7(8):31. doi:10.3390/tropicalmed7080161

16. Arias Ramos D, Alzate JA, Moreno Gómez GA, et al. Empirical treatment and mortality in bacteremia due to extended spectrum β-lactamase producing Enterobacterales (ESβL-E), a retrospective cross-sectional study in a tertiary referral hospital from Colombia. *Ann Clin Microbiol Antimicrob*. 2023;22(1). doi:10.1186/s12941-023-00566-2

17. Aryee A, Rockenschaub P, Gill MJ, Hayward A, Shallcross L. The relationship between clinical outcomes and empirical antibiotic therapy in patients with community-onset Gram-negative bloodstream infections: a cohort study from a large teaching hospital. *Epidemiol Infect*. 2020;148:e225. doi:10.1017/S0950268820002083

18. Aslan AT, Kirbas E, Sancak B, et al. A retrospective observational cohort study of the clinical epidemiology of bloodstream infections due to carbapenem-resistant Klebsiella pneumoniae in an OXA-48 endemic setting. *Int J Antimicrob Agents*. 2022;59(4):106554. doi:10.1016/j.ijantimicag.2022.106554

19. Atamna A, Hamud H, Daud W, Shochat T, Bishara J, Elis A. Chronic use of oral iron supplements is associated with poor clinical outcomes in patients with gram-negative bacteremia. *Eur J Clin Microbiol Infect Dis*. 2019;38(4):689-693. doi:10.1007/s10096-019-03481-7

20. Ayaz CM, Turhan O, Yilmaz VT, Adanir H, Sezer B, Ogunc D. Can the pan-immune-inflammation value predict gram negative bloodstream infection-related 30-day mortality in solid organ transplant patients? *BMC Infect Dis*. 2024;24(1):526. doi:10.1186/s12879-024-09413-x

21. Babich T, Naucler P, Valik JK, et al. Risk factors for mortality among patients with Pseudomonas aeruginosa bacteraemia: a retrospective multicentre study. *Int J Antimicrob Agents*. 2020;55(2):105847. doi:10.1016/j.ijantimicag.2019.11.004

22. Balkan II, Batirel A, Karabay O, et al. Comparison of colistin monotherapy and non-colistin combinations in the treatment of multi-drug resistant Acinetobacter spp. bloodstream infections: a multicenter retrospective analysis. *Indian J Pharmacol*. 2015;47(1):95-100. doi:10.4103/0253-7613.150383

23. Baltas I, Stockdale T, Tausan M, et al. Impact of antibiotic timing on mortality from Gram-negative bacteraemia in an English district general hospital: the importance of getting it right every time. *J Antimicrob Chemother*. 2021;76(3):813-819. doi:10.1093/jac/dkaa478

© 2025 La P et al. *JAMA Network Open*.

24. Bass SN, Bauer SR, Neuner EA, Lam SW. Impact of combination antimicrobial therapy on mortality risk for critically ill patients with carbapenem-resistant bacteremia. *Antimicrob Agents Chemother*. 2015;59(7):3748-3753. doi:10.1128/AAC.00091-15

25. Battle SE, Augustine MR, Watson CM, et al. Derivation of a quick Pitt bacteremia score to predict mortality in patients with Gram-negative bloodstream infection. *Infection*. 2019;47(4):571-578. doi:10.1007/s15010-019-01277-7

26. Bavaro DF, Papagni R, Belati A, et al. Cefiderocol Versus Colistin for the Treatment of Carbapenem-Resistant Acinetobacter baumannii Complex Bloodstream Infections: A Retrospective, Propensity-Score Adjusted, Monocentric Cohort Study. *Infect Dis Ther*. 2023;12(8):2147-2163. doi:10.1007/s40121-023-00854-6

27. Ben-David D, Kordevani R, Keller N, et al. Outcome of carbapenem resistant Klebsiella pneumoniae bloodstream infections. *Clin Microbiol Infect*. 2012;18(1):54-60. doi:10.1111/j.1469-0691.2011.03478.x

28. Benanti GE, Brown ART, Shigle TL, et al. Carbapenem versus Cefepime or Piperacillin-Tazobactam for Empiric Treatment of Bacteremia Due to Extended-Spectrum-beta-Lactamase-Producing Escherichia coli in Patients with Hematologic Malignancy. *Antimicrob Agents Chemother*. 2019;63(2):02. doi:10.1128/AAC.01813-18

29. Biehle LR, Cottreau JM, Thompson DJ, et al. Outcomes and Risk Factors for Mortality among Patients Treated with Carbapenems for Klebsiella spp. Bacteremia. *PLoS ONE Electron Resour*. 2015;10(11):e0143845. doi:10.1371/journal.pone.0143845

30. Blandy O, Honeyford K, Gharbi M, et al. Factors that impact on the burden of Escherichia coli bacteraemia: multivariable regression analysis of 2011-2015 data from West London. *J Hosp Infect*. 2019;101(2):120-128. doi:10.1016/j.jhin.2018.10.024

31. Boattini M, Bianco G, Charrier L, et al. Rapid diagnostics and ceftazidime/avibactam for KPC-producing Klebsiella pneumoniae bloodstream infections: impact on mortality and role of combination therapy. *Eur J Clin Microbiol Infect Dis*. 2023;42(4):431-439. doi:10.1007/s10096-023-04577-x

32. Bowers DR, Liew YX, Lye DC, Kwa AL, Hsu LY, Tam VH. Outcomes of appropriate empiric combination versus monotherapy for Pseudomonas aeruginosa bacteremia. *Antimicrob Agents Chemother*. 2013;57(3):1270-1274. doi:10.1128/AAC.02235-12

33. Bryant RE, Hood AF, Hood CE, Koenig MG. Factors affecting mortality of gram-negative rod bacteremia. *Arch Intern Med*. 1971;127(1):120-128.

34. Burnham JP, Micek ST, Kollef MH. Augmented renal clearance is not a risk factor for mortality in Enterobacteriaceae bloodstream infections treated with appropriate empiric antimicrobials. *PLoS ONE Electron Resour*. 2017;12(7):e0180247. doi:10.1371/journal.pone.0180247

35. Cain SE, Kohn J, Bookstaver PB, Albrecht H, Al-Hasan MN. Stratification of the impact of inappropriate empirical antimicrobial therapy for Gram-negative bloodstream infections by predicted prognosis. *Antimicrob Agents Chemother*. 2015;59(1):245-250. doi:10.1128/AAC.03935-14

36. Camins BC, Marschall J, DeVader SR, Maker DE, Hoffman MW, Fraser VJ. The clinical impact of fluoroquinolone resistance in patients with E coli bacteremia. *J Hosp Med Online*. 2011;6(6):344-349. doi:10.1002/jhm.877

37. Caydasi O, Arslan E, Cetin AS, et al. Risk Factors and Outcomes of Patients with Pseudomonas aeruginosa Bloodstream Infection in the Intensive Care Unit. *Jundishapur J Microbiol*. 2024;17(8). doi:10.5812/jjm-150331

38. Ceken S, Iskender G, Gedik H, et al. Risk factors for bloodstream infections due to extended-spectrum beta-lactamase producing Enterobacteriaceae in cancer patients. *J Infect Dev Ctries*. 2018;12(4):265-272. doi:10.3855/jidc.9720

39. Cetin S, Dokmetas I, Hamidi AA, Bayraktar B, Gunduz A, Sevgi DY. Comparison of Risk Factors and Outcomes in Carbapenem-Resistant and Carbapenem-Susceptible Gram-Negative Bacteremia. *Sisli Etfal Hastan Tp Bul*. 2021;55(3):398-404. doi:10.14744/SEMB.2020.49002

40. Chang H, Wei J, Zhou W, et al. Risk factors and mortality for patients with Bloodstream infections of Klebsiella pneumoniae during 2014-2018: Clinical impact of carbapenem resistance in a large tertiary hospital of China. *J Infect Public Health*. 2020;13(5):784-790. doi:10.1016/j.jiph.2019.11.014

41. Chapelet G, Boureau AS, Dylis A, et al. Association between dementia and reduced walking ability and 30-day mortality in patients with extended-spectrum beta-lactamase-producing Escherichia coli bacteremia. *Eur J Clin Microbiol Infect Dis*. 2017;36(12):2417-2422. doi:10.1007/s10096-017-3077-6

42. Chaubey VP, Pitout JDD, Dalton B, Gregson DB, Ross T, Laupland KB. Clinical and microbiological characteristics of bloodstream infections due to AmpC β-lactamase producing Enterobacteriaceae: An active surveillance cohort in a large centralized Canadian region. *BMC Infect Dis*. 2014;14(1). doi:10.1186/s12879-014-0647-4

43. Chen CL, Hou PC, Wang YT, et al. The High mortality and antimicrobial resistance of Klebsiella pneumoniae bacteremia in northern Taiwan. *J Infect Dev Ctries*. 2020;14(4):373-379. doi:10.3855/jidc.11524

44. Chen CP, Yang YY, Tsai IT, Hsu YC. The Prognostic Value of Time to Positivity of Klebsiella Pneumoniae in Blood Cultures of Elderly Patients With Intra-Abdominal Infection. *J Acute Med*. 2023;13(4):137-143. doi:10.6705/j.jacme.202312_13(4).0001

45. Chen FC, Ho YN, Cheng HH, Wu CH, Change MW, Su CM. Does inappropriate initial antibiotic therapy affect in-hospital mortality of patients in the emergency department with Escherichia coli and Klebsiella pneumoniae bloodstream infections? *Int J Immunopathol Pharmacol*. 2020;34:2058738420942375. doi:10.1177/2058738420942375

46. Chen F, Lv T, Xiao Y, Chen A, Xiao Y, Chen Y. Clinical Characteristics of Patients and Whole Genome Sequencing-Based Surveillance of Escherichia coli Community-Onset Bloodstream Infections at a Non-tertiary Hospital in CHINA. *Front Microbiol*. 2021;12:748471. doi:10.3389/fmicb.2021.748471

© 2025 La P et al. *JAMA Network Open*.

47. Chen IR, Lin SN, Wu XN, Chou SH, Wang FD, Lin YT. Clinical and Microbiological Characteristics of Bacteremic Pneumonia Caused by Klebsiella pneumoniae. *Front Cell Infect Microbiol*. 2022;12:903682. doi:10.3389/fcimb.2022.903682

48. Chen J, Ma H, Huang XM, et al. Risk factors and mortality of carbapenem-resistant Klebsiella pneumoniae bloodstream infection in a tertiary-care hospital in China: an eight-year retrospective study. *Antimicrob Resist Infect Control*. 2022;11(1):12. doi:10.1186/s13756-022-01204-w

49. Chen L, Han X, Li Y, Li M. Assessment of Mortality-Related Risk Factors and Effective Antimicrobial Regimens for Treatment of Bloodstream Infections Caused by Carbapenem-Resistant Enterobacterales. *Antimicrob Agents Chemother*. 2021;65(9):e0069821. doi:10.1128/AAC.00698-21

50. Chen WC, Hung CH, Chen YS, et al. Bloodstream infections caused by extended-spectrum beta-lactamase-producing escherichia coli in patients with liver cirrhosis. *Pathogens*. 2021;10(1):1-13. doi:10.3390/pathogens10010037

51. Chen YL, Chen Y, Liu PJ, et al. Risk factors and mortality for elderly patients with bloodstream infection of carbapenem resistance Klebsiella pneumoniae: a 10-year longitudinal study. *BMC Geriatr*. 2022;22(1):8. doi:10.1186/s12877-022-03275-1

52. Chen Y, Ying S, Qiu Y, et al. A Novel Nomogram for Predicting Risk Factors and Outcomes in Bloodstream Infections Caused by Klebsiella pneumoniae. *Infect Drug Resist*. 2022;15:1317-1328. doi:10.2147/IDR.S349236

53. Cheng A, Chuang YC, Sun HY, et al. Should we treat patients with only one set of positive blood cultures for extensively drug-resistant Acinetobacter baumannii the same as multiple sets? *PLoS ONE Electron Resour*. 2017;12(7):e0180967. doi:10.1371/journal.pone.0180967

54. Cheng WL, Hsueh PR, Lee CC, et al. Bacteremic pneumonia caused by extended-spectrum beta-lactamase-producing Escherichia coli and Klebsiella pneumoniae: Appropriateness of empirical treatment matters. *J Microbiol Immunol Infect*. 2016;49(2):208-215. doi:10.1016/j.jmii.2014.05.003

55. Cheng Y, Cheng Q, Zhang R, et al. Retrospective analysis of molecular characteristics, risk factors, and outcomes in carbapenem-resistant Klebsiella pneumoniae bloodstream infections. *BMC Microbiol*. 2024;24(1):309. doi:10.1186/s12866-024-03465-4

56. Cheong HS, Kang CI, Kwon KT, et al. Clinical significance of healthcare-associated infections in community-onset Escherichia coli bacteraemia. *J Antimicrob Chemother*. 2007;60(6):1355-1360. doi:10.1093/jac/dkm378

57. Cheong HS, Kang CI, Wi YM, et al. Clinical significance and predictors of community-onset Pseudomonas aeruginosa bacteremia. *Am J Med*. 2008;121(8):709-714. doi:10.1016/j.amjmed.2008.03.034

58. Cheong HS, Kang CI, Wi YM, et al. Inappropriate initial antimicrobial therapy as a risk factor for mortality in patients with community-onset Pseudomonas aeruginosa

© 2025 La P et al. *JAMA Network Open*.

bacteraemia. *Eur J Clin Microbiol Infect Dis*. 2008;27(12):1219-1225. doi:10.1007/s10096-008-0568-5

59. Cheong HS, Ko KS, Kang CI, Chung DR, Peck KR, Song JH. Clinical significance of infections caused by extended-spectrum beta-lactamase-producing Enterobacteriaceae blood isolates with inducible AmpC beta-lactamase. *Microb Drug Resist-Mech Epidemiol Dis*. 2012;18(4):446-452. doi:10.1089/mdr.2011.0126

60. Cheong HS, Lee JA, Kang CI, et al. Risk factors for mortality and clinical implications of catheter-related infections in patients with bacteraemia caused by Stenotrophomonas maltophilia. *Int J Antimicrob Agents*. 2008;32(6):538-540. doi:10.1016/j.ijantimicag.2008.05.011

61. Chiang TT, Huang TW, Sun JR, et al. Biofilm formation is not an independent risk factor for mortality in patients with Acinetobacter baumannii bacteremia. *Front Cell Infect Microbiol*. 2022;12:964539. doi:10.3389/fcimb.2022.964539

62. Chiong F, Wasef MS, Liew KC, et al. The impact of infectious diseases consultation on the management and outcomes of Pseudomonas aeruginosa bacteraemia in adults: a retrospective cohort study. *BMC Infect Dis*. 2021;21(1):671. doi:10.1186/s12879-021-06372-5

63. Cho SY, Kang CI, Cha MK, et al. Clinical features and treatment outcomes of bloodstream infections caused by extended-spectrum β-lactamase-producing escherichia coli sequence type 131. *Microb Drug Resist*. 2015;21(4):463-469. doi:10.1089/mdr.2014.0261

64. Choi SH, Cho EB, Chung JW, Lee MK. Changes in the early mortality of adult patients with carbapenem-resistant Acinetobacter baumannii bacteremia during 11 years at an academic medical center. *J Infect Chemother*. 2019;25(1):6-11. doi:10.1016/j.jiac.2018.09.011

65. Chopra T, Marchaim D, Veltman J, et al. Impact of cefepime therapy on mortality among patients with bloodstream infections caused by extended-spectrum-β-lactamase-producing Klebsiella pneumoniae and Escherichia coli. *Antimicrob Agents Chemother*. 2012;56(7):3936-3942. doi:10.1128/AAC.05419-11

66. Chow SF, Sheu DC, Liu CP. Biochemical and haematological parameters and the impact of hypoalbuminaemia on mortality in carbapenem-resistant Acinetobacter baumannii complex bacteraemia. *Res J Biotechnol*. 2015;10(8):11-18.

67. Chuang HC, Ho YH, Lay CJ, Wang LS, Tsai YS, Tsai CC. Different clinical characteristics among Aeromonas hydrophila, Aeromonas veronii biovar sobria and Aeromonas caviae monomicrobial bacteremia. *J Korean Med Sci*. 2011;26(11):1415-1420. doi:10.3346/jkms.2011.26.11.1415

68. Chuang YC, Sheng WH, Li SY, et al. Influence of genospecies of Acinetobacter baumannii complex on clinical outcomes of patients with acinetobacter bacteremia. *Clin Infect Dis*. 2011;52(3):352-360. doi:10.1093/cid/ciq154

69. Chung HC, Lai CH, Lin JN, et al. Bacteremia caused by extended-spectrum-beta-lactamase-producing Escherichia coli sequence type ST131 and non-ST131 clones:

comparison of demographic data, clinical features, and mortality. *Antimicrob Agents Chemother*. 2012;56(2):618-622. doi:10.1128/AAC.05753-11

70. Cooper L, Yu K, Van Benten K, et al. Hospital mortality and length of stay associated with Enterobacterales positive blood cultures: a multicenter analysis. *Microbiol Spectr*. 2024;12(8):e0040224. doi:10.1128/spectrum.00402-24

71. Corcione S, De Benedetto I, Shbaklo N, et al. Ten Years of KPC-Kp Bloodstream Infections Experience: Impact of Early Appropriate Empirical Therapy on Mortality. *Biomedicines*. 2022;10(12):12. doi:10.3390/biomedicines10123268

72. Cristina ML, Alicino C, Sartini M, et al. Epidemiology, management, and outcome of carbapenem-resistant Klebsiella pneumoniae bloodstream infections in hospitals within the same endemic metropolitan area. *J Infect Public Health*. 2018;11(2):171-177. doi:10.1016/j.jiph.2017.06.003

73. De Rosa FG, Pagani N, Fossati L, et al. The effect of inappropriate therapy on bacteremia by ESBL-producing bacteria. *Infection*. 2011;39(6):555-561. doi:10.1007/s15010-011-0201-x

74. Dekic M, Dugandzija T, Dragovac G, Medic D, Paut Kusturica M. Risk factors and clinical outcomes for intensive care unit patients with mul-tidrug-resistant Acinetobacter spp. bacteremia. *Hippokratia*. 2020;24(1):21-26.

75. Delgado-Valverde M, Torres E, Valiente-Mendez A, et al. Impact of the MIC of piperacillin/tazobactam on the outcome for patients with bacteraemia due to Enterobacteriaceae: the Bacteraemia-MIC project. *J Antimicrob Chemother*. 2016;71(2):521-530. doi:10.1093/jac/dkv362

76. Derin O, Sahin M, Dumlu R, et al. Registry-Based Retrospective Cohort Study of Mortality among Adults Admitted to Intensive Care Units in Istanbul with Hospital Acquired Pseudomonas aeruginosa Bloodstream-Infection between 2014-2021. *Antibiotics*. 2024;13(1):17. doi:10.3390/antibiotics13010090

77. Drozdinsky G, Neuberger A, Rakedzon S, et al. Treatment of Bacteremia Caused by Enterobacter spp.: Should the Potential for AmpC Induction Dictate Therapy? A Retrospective Study. *Microb Drug Resist-Mech Epidemiol Dis*. 2021;27(3):410-414. doi:10.1089/mdr.2020.0234

78. Dumlu R, Sahin M, Derin O, et al. Ceftazidime-Avibactam Versus Polymyxin-Based Combination Therapies: A Study on 30-Day Mortality in Carbapenem-Resistant Enterobacterales Bloodstream Infections in an OXA-48-Endemic Region. *Antibiotics*. 2024;13(10):18. doi:10.3390/antibiotics13100990

79. Durdu B, Hakyemez IN, Bolukcu S, Okay G, Gultepe B, Aslan T. Mortality markers in nosocomial Klebsiella pneumoniae bloodstream infection. *Springerplus*. 2016;5(1):1892. doi:10.1186/s40064-016-3580-8

80. Erbay A, Idil A, Gözel MG, Mumcuoğlu I, Balaban N. Impact of early appropriate antimicrobial therapy on survival in Acinetobacter baumannii bloodstream infections. *Int J Antimicrob Agents*. 2009;34(6):575-579. doi:10.1016/j.ijantimicag.2009.07.006

© 2025 La P et al. *JAMA Network Open*.

81. Ergonul O, Aydin M, Azap A, et al. Healthcare-associated Gram-negative bloodstream infections: antibiotic resistance and predictors of mortality. *J Hosp Infect*. 2016;94(4):381-385. doi:10.1016/j.jhin.2016.08.012

82. Escrihuela-Vidal F, Palacios-Baena ZR, Agirre JG, et al. Early antibiotic de-escalation in patients with severe infections due to bloodstream infection by enterobacterales: A post hoc analysis of a prospective multicentre cohort. *Int J Antimicrob Agents*. 2024;64(5):107317. doi:10.1016/j.ijantimicag.2024.107317

83. Falcone M, Bassetti M, Tiseo G, et al. Time to appropriate antibiotic therapy is a predictor of outcome in patients with bloodstream infection caused by KPC-producing Klebsiella pneumoniae. *Crit Care Lond Engl*. 2020;24(1):29. doi:10.1186/s13054-020-2742-9

84. Falcone M, Russo A, Iacovelli A, et al. Predictors of outcome in ICU patients with septic shock caused by Klebsiella pneumoniae carbapenemase-producing K. pneumoniae. *Clin Microbiol Infect*. 2016;22(5):444-450. doi:10.1016/j.cmi.2016.01.016

85. Falcone M, Tiseo G, Carbonara S, et al. Mortality Attributable to Bloodstream Infections Caused by Different Carbapenem-Resistant Gram-Negative Bacilli: Results From a Nationwide Study in Italy (ALARICO Network). *Clin Infect Dis*. 2023;76(12):2059-2069. doi:10.1093/cid/ciad100

86. Fang Y, Zhong Q, Chen Y, et al. Ceftazidime/Avibactam, Polymyxin or Tigecycline as a Rescue Strategy for the Treatment of Carbapenem-Resistant Klebsiella pneumoniae in Bloodstream Infection: A Retrospective Cohort Study. *Infect Drug Resist*. 2023;16:2963-2971. doi:10.2147/IDR.S409506

87. Fatima M, Dodani SK, Babar ZU, et al. Outcome, risk factors and therapeutic strategies in carbapenem-resistant Gram-negative bacteraemia from Pakistan. *JAC-Antimicrob Resist*. 2023;5(3):dlad076. doi:10.1093/jacamr/dlad076

88. Ferreira TDC, Martins IS. Risk factors of death in bloodstream infections caused by ampc β-lactamase-producing enterobacterales in patients with neoplasia. *Infect Drug Resist*. 2021;14:3083-3097. doi:10.2147/IDR.S312920

89. Fitzpatrick JM, Biswas JS, Edgeworth JD, et al. Gram-negative bacteraemia; a multi-centre prospective evaluation of empiric antibiotic therapy and outcome in English acute hospitals. *Clin Microbiol Infect*. 2016;22(3):244-251. doi:10.1016/j.cmi.2015.10.034

90. Florez Riano AF, Rojas Castro OJ, Ospina S, Ramirez-Sanchez IC. Association between inappropriate empirical antimicrobial therapy and mortality in gram-negative bloodstream infections in patients with febrile neutropenia and hematological malignancy. *J Infect Chemother*. 2024;11:11. doi:10.1016/j.jiac.2024.10.006

91. Fostervold A, Raffelsberger N, Hetland MAK, et al. Risk of death in Klebsiella pneumoniae bloodstream infections is associated with specific phylogenetic lineages. *J Infect*. 2024;88(5):106155. doi:10.1016/j.jinf.2024.106155

92. Fraenkel-Wandel Y, Raveh-Brawer D, Wiener-Well Y, Yinnon AM, Assous MV. Mortality due to blaKPC Klebsiella pneumoniae bacteraemia. *J Antimicrob Chemother*. 2016;71(4):1083-1087. doi:10.1093/jac/dkv414

© 2025 La P et al. *JAMA Network Open*.

93. Fu Q, Ye H, Liu S. Risk factors for extensive drug-resistance and mortality in geriatric inpatients with bacteremia caused by Acinetobacter baumannii. *Am J Infect Control*. 2015;43(8):857-860. doi:10.1016/j.ajic.2015.03.033

94. Fuentes-Gonzalez MF, Fernandez-Rodriguez D, Colin-Castro CA, Hernandez-Duran M, Lopez-Jacome LE, Franco-Cendejas R. Gram-Negative Bacilli Blood Stream Infection in Patients with Severe Burns: Microbiological and Clinical Evidence from a 9-Year Cohort. *Int J Mol Sci*. 2024;25(19):28. doi:10.3390/ijms251910458

95. Galofré J, Moreno A, Mensa J, et al. Analysis of factors influencing the outcome and development of septic metastasis or relapse in salmonella bacteremia. *Clin Infect Dis*. 1994;18(6):873-878.

96. Gao Y, Lin H, Xu Y, et al. Prognostic Risk Factors of Carbapenem-Resistant Gram-Negative Bacteria Bloodstream Infection in Immunosuppressed Patients: A 7-Year Retrospective Cohort Study. *Infect Drug Resist*. 2022;15:6451-6462. doi:10.2147/IDR.S386342

97. Gezer Y, Taysi MR, Tarakci A, et al. Evaluation of clinical outcomes and risk factors associated with mortality in patients with Stenotrophomonas maltophilia bloodstream infection: a multicenter study. *BMC Infect Dis*. 2024;24(1):1387. doi:10.1186/s12879-024-10293-4

98. Giannella M, Pascale R, Pancaldi L, et al. Follow-up blood cultures are associated with improved outcome of patients with gram-negative bloodstream infections: retrospective observational cohort study. *Clin Microbiol Infect*. 2020;26(7):897-903. doi:10.1016/j.cmi.2020.01.023

99. Girometti N, Lewis RE, Giannella M, et al. Klebsiella pneumoniae bloodstream infection epidemiology and impact of inappropriate empirical therapy. *Med U S*. 2014;93(17):298-308. doi:10.1097/MD.0000000000000111

100. Gomez-Simmonds A, Greenman M, Sullivan SB, et al. Population Structure of Klebsiella pneumoniae Causing Bloodstream Infections at a New York City Tertiary Care Hospital: Diversification of Multidrug-Resistant Isolates. *J Clin Microbiol*. 2015;53(7):2060-2067. doi:10.1128/JCM.03455-14

101. Gomez-Simmonds A, Nelson B, Eiras DP, et al. Combination Regimens for Treatment of Carbapenem-Resistant Klebsiella pneumoniae Bloodstream Infections. *Antimicrob Agents Chemother*. 2016;60(6):3601-3607. doi:10.1128/AAC.03007-15

102. Gözel MG, Erbay A, Bodur H, Eren SS, Balaban N. Risk factors for mortality in patients with nosocomial gram-negative bacteremia. *Turk Klin J Med Sci*. 2012;32(6):1641-1647. doi:10.5336/medsci.2011-27342

103. Gradel KO, Schonheyder HC, Pedersen L, Thomsen RW, Norgaard M, Nielsen H. Incidence and prognosis of non-typhoid Salmonella bacteraemia in Denmark: a 10-year county-based follow-up study. *Eur J Clin Microbiol Infect Dis*. 2006;25(3):151-158. doi:10.1007/s10096-006-0110-6

© 2025 La P et al. *JAMA Network Open*.

104. Gu Z, Han Y, Meng T, et al. Risk Factors and Clinical Outcomes for Patients With Acinetobacter baumannii Bacteremia. *Medicine (Baltimore)*. 2016;95(9):e2943. doi:10.1097/MD.0000000000002943

105. Gutiérrez-Gutiérrez B, Pérez-Galera S, Salamanca E, et al. A multinational, preregistered cohort study of β-lactam/β-lactamase inhibitor combinations for treatment of bloodstream infections due to extended-spectrum-β-lactamase-producing enterobacteriaceae. *Antimicrob Agents Chemother*. 2016;60(7):4159-4169. doi:10.1128/AAC.00365-16

106. Hazwan NM, Najma K, Ramliza R, Rozita H, Chee LL, Petrick P. Risk Factors for Severe Outcomes in Extended-Spectrum Beta-Lactamase (ESBL) Bacteremia: A Single-center Study. *Med Health*. 2022;17(2):56-69. doi:10.17576/mh.2022.1702.05

107. Herrera S, Morata L, Sempere A, et al. Pseudomonas aeruginosa Bloodstream Infection, Resistance, and Mortality: Do Solid Organ Transplant Recipients Do Better or Worse? *Antibiotics*. 2023;12(2):13. doi:10.3390/antibiotics12020380

108. Hirsch EB, Cottreau JM, Chang KT, Caeiro JP, Johnson ML, Tam VH. A model to predict mortality following Pseudomonas aeruginosa bacteremia. *Diagn Microbiol Infect Dis*. 2012;72(1):97-102. doi:10.1016/j.diagmicrobio.2011.09.018

109. Hodgin UG, Sanford JP. Gram-negative rod bacteremia. An analysis of 100 patients. *Am J Med*. 1965;39(6):952-960. doi:10.1016/0002-9343(65)90118-x

110. Hojat LS, Wilson BM, Satlin MJ, et al. 14-Year Epidemiologic study of Pseudomonas aeruginosa bloodstream infection incidence and resistance in the Veterans Health Administration system, 2009-2022. *JAC-Antimicrob Resist*. 2024;6(2):dlae031. doi:10.1093/jacamr/dlae031

111. Hou W, Han T, Qu G, Sun Y, Yang D, Lin Y. Is early time to positivity of blood culture associated with clinical prognosis in patients with Klebsiella pneumoniae bloodstream infection? *Epidemiol Infect*. 2023;151:e43. doi:10.1017/S0950268823000262

112. Hsieh CC, Lee CH, Li MC, Hong MY, Chi CH, Lee CC. Empirical third-generation cephalosporin therapy for adults with community-onset Enterobacteriaceae bacteraemia: Impact of revised CLSI breakpoints. *Int J Antimicrob Agents*. 2016;47(4):297-303. doi:10.1016/j.ijantimicag.2016.01.010

113. Hsu JY, Chuang YC, Wang JT, Chen YC, Hsieh SM. Healthcare-associated carbapenem-resistant Klebsiella pneumoniae bloodstream infections: Risk factors, mortality, and antimicrobial susceptibility, 2017-2019. *J Formos Med Assoc*. 2021;120(11):1994-2002. doi:10.1016/j.jfma.2021.04.014

114. Huang C, Gao Y, Lin H, Fan Q, Chen L, Feng Y. Prognostic Factors That Affect Mortality Patients with Acinetobacter baumannii Bloodstream Infection. *Infect Drug Resist*. 2024;17:3825-3837. doi:10.2147/IDR.S475073

115. Huang CH, Tsai JS, Chen IW, Hsu BR, Huang MJ, Huang YY. Risk factors for in-hospital mortality in patients with type 2 diabetes complicated by community-acquired Klebsiella pneumoniae bacteremia. *J Formos Med Assoc*. 2015;114(10):916-922. doi:10.1016/j.jfma.2015.07.011

© 2025 La P et al. *JAMA Network Open*.

116. Huang X, Ding J, Yang X, et al. Clinical characteristics and prognosis analysis of pseudomonas aeruginosa bloodstream infection in adults: a retrospective study. *Clin Exp Med*. 2025;25(1). doi:10.1007/s10238-024-01517-7

117. Huh K, Chung DR, Ha YE, et al. Impact of Difficult-to-Treat Resistance in Gram-negative Bacteremia on Mortality: Retrospective Analysis of Nationwide Surveillance Data. *Clin Infect Dis*. 2020;71(9):e487-e496. doi:10.1093/cid/ciaa084

118. Huh K, Kang CI, Kim J, et al. Risk factors and treatment outcomes of bloodstream infection caused by extended-spectrum cephalosporin-resistant Enterobacter species in adults with cancer. *Diagn Microbiol Infect Dis*. 2014;78(2):172-177. doi:10.1016/j.diagmicrobio.2013.11.002

119. Imai K, Ishibashi N, Kodana M, et al. Clinical characteristics in blood stream infections caused by Klebsiella pneumoniae, Klebsiella variicola, and Klebsiella quasipneumoniae: a comparative study, Japan, 2014-2017. *BMC Infect Dis*. 2019;19(1):946. doi:10.1186/s12879-019-4498-x

120. Ioannou P, Alexakis K, Maraki S, Kofteridis DP. Pseudomonas Bacteremia in a Tertiary Hospital and Factors Associated with Mortality. *Antibiotics*. 2023;12(4):29. doi:10.3390/antibiotics12040670

121. Isler B, Ozer B, Cinar G, et al. Characteristics and outcomes of carbapenemase harbouring carbapenem-resistant Klebsiella spp. bloodstream infections: a multicentre prospective cohort study in an OXA-48 endemic setting. *Eur J Clin Microbiol Infect Dis*. 2022;41(5):841-847. doi:10.1007/s10096-022-04425-4

122. Iwasaki M, Ishikawa K, Isoya T, Matsumoto-Takahashi ELA, Mori N. Association between in-hospital weight change and 28-day mortality in adults with gram-negative rod bacteremia. *Infect Dis Now*. 2025;55(1). doi:10.1016/j.idnow.2024.105015

123. Jeon M, Huh K, Ko JH, et al. Difference in the Clinical Outcome of Bloodstream Infections Caused by Klebsiella aerogenes and Enterobacter cloacae Complex. *Open Forum Infect Dis*. 2021;8(8):ofab390. doi:10.1093/ofid/ofab390

124. Jian X, Du S, Zhou X, et al. Development and validation of nomograms for predicting the risk probability of carbapenem resistance and 28-day all-cause mortality in gram-negative bacteremia among patients with hematological diseases. *Front Cell Infect Microbiol*. 2022;12:969117. doi:10.3389/fcimb.2022.969117

125. Joo EJ, Park DA, Lee NR, et al. Impact of appropriateness of empiric therapy on outcomes in community-onset bacteremia by extended-spectrum-beta-lactamase producing Escherichia coli and Klebisella pneumoniae definitively treated with carbapenems. *Eur J Clin Microbiol Infect Dis*. 2017;36(11):2093-2100. doi:10.1007/s10096-017-3031-7

126. Kaki R. Retrospective analysis of Acinetobacter baumannii bacteraemia risk factors, complications and mortality in a tertiary university hospital in Saudi Arabia. *Access Microbiol*. 2024;6(9). doi:10.1099/acmi.0.000826.v4

© 2025 La P et al. *JAMA Network Open*.

127. Kalam K, Qamar F, Kumar S, Ali S, Baqi S. Risk factors for carbapenem resistant bacteraemia and mortality due to gram negative bacteraemia in a developing country. *JPMA - J Pak Med Assoc*. 2014;64(5):530-536.

128. Kanchanasuwan S, Rongmuang J, Siripaitoon P, et al. Clinical Characteristics, Outcomes, and Risk Factors for Mortality in Patients with Stenotrophomonas maltophilia Bacteremia. *J Clin Med*. 2022;11(11):30. doi:10.3390/jcm11113085

129. Kang CI, Kim SH, Wan BP, et al. Bloodstream infections due to extended-spectrum β-lactamase-producing Escherichia coli and Klebsiella pneumoniae: Risk factors for mortality and treatment outcome, with special emphasis on antimicrobial therapy. *Antimicrob Agents Chemother*. 2004;48(12):4574-4581. doi:10.1128/AAC.48.12.4574-4581.2004

130. Kang CI, Song JH, Chung DR, et al. Risk factors and pathogenic significance of severe sepsis and septic shock in 2286 patients with gram-negative bacteremia. *J Infect*. 2011;62(1):26-33. doi:10.1016/j.jinf.2010.10.010

131. Kang FY, How CK, Wang YC, et al. Influence of severity of infection on the effect of appropriate antimicrobial therapy for Acinetobacter baumannii bacteremic pneumonia. *Antimicrob Resist Infect Control*. 2020;9(1):160. doi:10.1186/s13756-020-00824-4

132. Karaiskos I, Daikos GL, Gkoufa A, et al. Ceftazidime/avibactam in the era of carbapenemase-producing Klebsiella pneumoniae: experience from a national registry study. *J Antimicrob Chemother*. 2021;76(3):775-783. doi:10.1093/jac/dkaa503

133. Khamis F, Al-Zakwani I, Molai M, et al. Demographic, clinical, and outcome characteristics of carbapenem-resistant Enterobacteriaceae over a 10-year period (2010-2020) in Oman. *IJID Reg Online*. 2022;4:165-170. doi:10.1016/j.ijregi.2022.08.001

134. Kim D, Park BY, Choi MH, et al. Antimicrobial resistance and virulence factors of Klebsiella pneumoniae affecting 30 day mortality in patients with bloodstream infection. *J Antimicrob Chemother*. 2019;74(1):190-199. doi:10.1093/jac/dky397

135. Kim HW, Yoon JH, Jin SJ, et al. Delta neutrophil index as a prognostic marker of early mortality in gram negative bacteremia. *Infect Chemother*. 2014;46(2):94-102. doi:10.3947/ic.2014.46.2.94

136. Kim SH, Jeon CH, Kim HT, Wi YM. Clinical characteristics and manifestations in patients with hypermucoviscous Klebsiella pneumoniae bacteremia from extra-hepatobiliary tract infection. *Infection*. 2023;51(3):689-696. doi:10.1007/s15010-022-01940-6

137. Kim SH, Wi YM, Peck KR. Clinical Effectiveness of Tetracycline-Class Agents Based Regimens in Patients With Carbapenem-Resistant Acinetobacter baumannii Bacteremia: A Single-Center Retrospective Cohort Study. *J Korean Med Sci*. 2023;38(34):e263. doi:10.3346/jkms.2023.38.e263

138. Kim S, Ji S, Cho D, et al. Persistence of Stenotrophomonas maltophilia in Patients with Bacteremia: Incidence, Clinical and Microbiologic Characters, and Outcomes. *Microorganisms*. 2024;12(12):12. doi:10.3390/microorganisms12122477

© 2025 La P et al. *JAMA Network Open*.

139. Kim T, Park KH, Yu SN, et al. Early Intravenous Colistin Therapy as a Favorable Prognostic Factor for 28-day Mortality in Patients with CRAB Bacteremia: a Multicenter Propensity Score-Matching Analysis. *J Korean Med Sci*. 2019;34(39):e256. doi:10.3346/jkms.2019.34.e256

140. Kim YJ, Jun YH, Kim YR, et al. Risk factors for mortality in patients with Pseudomonas aeruginosa bacteremia; retrospective study of impact of combination antimicrobial therapy. *BMC Infect Dis*. 2014;14:161. doi:10.1186/1471-2334-14-161

141. Ko WC, Lee HC, Chuang YC, Liu CC, Wu JJ. Clinical features and therapeutic implications of 104 episodes of monomicrobial Aeromonas bacteraemia. *J Infect*. 2000;40(3):267-273. doi:10.1053/jinf.2000.0654

142. Komatsu Y, Kasahara K, Inoue T, et al. Molecular epidemiology and clinical features of extended-spectrum beta-lactamase- or carbapenemase-producing Escherichia coli bacteremia in Japan. *PLoS ONE Electron Resour*. 2018;13(8):e0202276. doi:10.1371/journal.pone.0202276

143. Kong ZX, Karunakaran RN, Jabar KA, Ponnampalavanar S, Chong CW, Teh CSJ. A retrospective study on molecular epidemiology trends of carbapenem resistant Enterobacteriaceae in a teaching hospital in Malaysia. *PeerJ*. 2022;10:18. doi:10.7717/peerj.12830

144. Ku NS, Kim HW, Oh HJ, et al. Red blood cell distribution width is an independent predictor of mortality in patients with gram-negative bacteremia. *Shock*. 2012;38(2):123-127. doi:10.1097/SHK.0b013e31825e2a85

145. Ku NS, Kim YC, Kim MH, et al. Risk factors for 28-day mortality in elderly patients with extended-spectrum beta-lactamase (ESBL)-producing Escherichia coli and Klebsiella pneumoniae bacteremia. *Arch Gerontol Geriatr*. 2014;58(1):105-109. doi:10.1016/j.archger.2013.07.002

146. Kuikka A, Sivonen A, Emelianova A, Valtonen VV. Prognostic factors associated with improved outcome of Escherichia coli bacteremia in a Finnish university hospital. *Eur J Clin Microbiol Infect Dis*. 1997;16(2):125-134. doi:10.1007/BF01709471

147. Kuo SC, Lee YT, Yang SP, et al. Evaluation of the effect of appropriate antimicrobial therapy on mortality associated with Acinetobacter nosocomialis bacteraemia. *Clin Microbiol Infect*. 2013;19(7):634-639. doi:10.1111/j.1469-0691.2012.03967.x

148. Kuo TH, Yang CY, Lee CH, Hsieh CC, Ko WC, Lee CC. Propensity score matched analysis comparing the clinical outcome of Klebsiella pneumoniae and Escherichia coli causing community-onset monomicrobial bacteremia. *Medicine (Baltimore)*. 2017;96(26):e7075. doi:10.1097/MD.0000000000007075

149. Kurt AF, Tanriverdi ES, Yalcin M, et al. Resistance Genes and Mortality in Carbapenem-resistant Klebsiella pneumoniae Bacteremias: Effects of the COVID-19 Pandemic. *Balk Med J*. 2024;41(5):357-368. doi:10.4274/balkanmedj.galenos.2024.2024-5-99

© 2025 La P et al. *JAMA Network Open*.

150. Lay CJ, Zhuang HJ, Ho YH, Tsai YS, Wang LS, Tsai CC. Different clinical characteristics between polymicrobial and monomicrobial aeromonas bacteremia-A study of 216 cases. *Intern Med*. 2010;49(22):2415-2421. doi:10.2169/internalmedicine.49.4117

151. Leao ACQ, Menezes PR, Oliveira MS, Levin AS. Acinetobacter spp. are associated with a higher mortality in intensive care patients with bacteremia: a survival analysis. *BMC Infect Dis*. 2016;16:8. doi:10.1186/s12879-016-1695-8

152. Lee CC, Lee CH, Chen PL, Hsieh CC, Tang HJ, Ko WC. Definitive Cefazolin Treatment for Community-Onset Enterobacteriaceae Bacteremia Based on the Contemporary CLSI Breakpoint: Clinical Experience of a Medical Center in Southern Taiwan. *Antibiotics*. 2019;8(4):10. doi:10.3390/antibiotics8040216

153. Lee CC, Lee CH, Hong MY, Hsieh CC, Tang HJ, Ko WC. Propensity-matched analysis of the impact of extended-spectrum beta-lactamase production on adults with community-onset Escherichia coli, Klebsiella species, and Proteus mirabilis bacteremia. *J Microbiol Immunol Infect*. 2018;51(4):519-526. doi:10.1016/j.jmii.2017.05.006

154. Lee CC, Lee NY, Chen PL, et al. Impact of antimicrobial strategies on clinical outcomes of adults with septic shock and community-onset Enterobacteriaceae bacteremia: de-escalation is beneficial. *Diagn Microbiol Infect Dis*. 2015;82(2):158-164. doi:10.1016/j.diagmicrobio.2015.03.004

155. Lee CC, Yang CY, Lee CH, et al. Clinical Benefit of Empiric High-Dose Levofloxacin Therapy for Adults With Community-onset Enterobacteriaceae Bacteremia. *Clin Ther*. 2019;41(10):1996-2007. doi:10.1016/j.clinthera.2019.07.010

156. Lee CM, Kim CJ, Kim SE, et al. Risk factors for early mortality in patients with carbapenem-resistant Acinetobacter baumannii bacteraemia. *J Glob Antimicrob Resist*. 2022;31:45-51. doi:10.1016/j.jgar.2022.08.010

157. Lee IR, Thein TL, Ang LW, et al. Cefazolin versus ceftriaxone as definitive treatment for Klebsiella pneumoniae bacteraemia: a retrospective multicentre study in Singapore. *J Antimicrob Chemother*. 2021;76(5):1303-1310. doi:10.1093/jac/dkab009

158. Lee NY, Chang TC, Wu CJ, et al. Clinical manifestations, antimicrobial therapy, and prognostic factors of monomicrobial Acinetobacter baumannii complex bacteremia. *J Infect*. 2010;61(3):219-227. doi:10.1016/j.jinf.2010.07.002

159. Lee NY, Lee CC, Huang WH, Tsui KC, Hsueh PR, Ko WC. Cefepime therapy for monomicrobial bacteremia caused by cefepime-susceptible extended-spectrum beta-lactamase-producing Enterobacteriaceae: MIC matters. *Clin Infect Dis*. 2013;56(4):488-495. doi:10.1093/cid/cis916

160. Lee NY, Lo CL, Chen PL, et al. Clinical impact of cefepime breakpoint in patients with carbapenem-resistant Klebsiella pneumoniae bacteraemia. *Int J Antimicrob Agents*. 2021;57(2):106250. doi:10.1016/j.ijantimicag.2020.106250

161. Lee SH, Kim CH, Lee HY, Park KH, Han SH. Epidemiology of Carbapenem-Resistant Enterobacteriaceae Bacteremia in Gyeonggi Province, Republic of Korea, between 2018 and 2021. *Antibiotics*. 2023;12(8). doi:10.3390/antibiotics12081286

© 2025 La P et al. *JAMA Network Open*.

162. Lee Y, Lee YT, Wang YC, et al. Risk of Mortality of Catheter-Related Bloodstream Infections Caused by Acinetobacter Species: Is Early Removal of the Catheters Associated With a Better Survival Outcome? *J Intensive Care Med*. 2018;33(6):361-369. doi:10.1177/0885066616677710

163. Lee YT, Huang TW, Liu IF, et al. The prediction values of carbapenemase detection methods and carbapenem susceptibility testing for clinical outcomes of patients with Acinetobacter bacteremia under carbapenem treatment. *J Microbiol Immunol Infect*. 2022;55(2):9. doi:10.1016/j.jmii.2021.03.013

164. Lee YT, Kuo SC, Yang SP, et al. Impact of appropriate antimicrobial therapy on mortality associated with Acinetobacter baumannii bacteremia: relation to severity of infection. *Clin Infect Dis*. 2012;55(2):209-215. doi:10.1093/cid/cis385

165. Lee YT, Wang YC, Kuo SC, et al. Multicenter Study of Clinical Features of Breakthrough Acinetobacter Bacteremia during Carbapenem Therapy. *Antimicrob Agents Chemother*. 2017;61(9):09. doi:10.1128/AAC.00931-17

166. Lefort A, Panhard X, Clermont O, et al. Host Factors and Portal of Entry Outweigh Bacterial Determinants To Predict the Severity of Escherichia coli Bacteremia. *J Clin Microbiol*. 2011;49(3):777-783. doi:10.1128/jcm.01902-10

167. Li CW, Chen PL, Lee NY, et al. Non-typhoidal Salmonella bacteremia among adults: An adverse prognosis in patients with malignancy. *J Microbiol Immunol Infect*. 2012;45(5):343-349. doi:10.1016/j.jmii.2011.12.015

168. Li F, Zhu J, Hang Y, et al. Clinical Characteristics and Prognosis of Hospital-Acquired Klebsiella pneumoniae Bacteremic Pneumonia versus Escherichia coli Bacteremic Pneumonia: A Retrospective Comparative Study. *Infect Drug Resist*. 2023;16:4977-4994. doi:10.2147/IDR.S419699

169. Li F, Zhu J, Zheng Y, Fang Y, Hu L, Xiong J. Comparison of bacteremic pneumonia caused by Escherichia coli and Klebsiella pneumoniae: A retrospective study. *Saudi Med J*. 2024;45(3):241-251. doi:10.15537/smj.2024.45.3.20230428

170. Li J, Ren J, Wang W, et al. Risk factors and clinical outcomes of hypervirulent Klebsiella pneumoniae induced bloodstream infections. *Eur J Clin Microbiol Infect Dis*. 2018;37(4):679-689. doi:10.1007/s10096-017-3160-z

171. Li J, Wu W, Wu M, et al. Clinical and Molecular Characteristics of Patients with Bloodstream Infections Caused by KPC and NDM Co-Producing Carbapenem-Resistant Klebsiella pneumoniae. *Infect Drug Resist*. 2024;17:1685-1697. doi:10.2147/IDR.S455146

172. Li L, Huang H. Risk factors of mortality in bloodstream infections caused by Klebsiella pneumonia: A single-center retrospective study in China. *Medicine (Baltimore)*. 2017;96(35):e7924. doi:10.1097/MD.0000000000007924

173. Li S, Jia XJ, Li CY, et al. Carbapenem-resistant and cephalosporin-susceptible Pseudomonas aeruginosa: a notable phenotype in patients with bacteremia. *Infect Drug Resist*. 2018;11:1225-1235. doi:10.2147/idr.S174876

174. Li X, Cai D, Mei C, Huang X. Construction and Validation of a Predictive Model for Mortality Risk in Patients with Acinetobacter baumannii Bloodstream Infection. *Infect Drug Resist*. 2024;17:5247-5260. doi:10.2147/IDR.S491537

175. Li X, Ye H. Clinical and Mortality Risk Factors in Bloodstream Infections with Carbapenem-Resistant Enterobacteriaceae. *Can J Infect Dis Med Microbiol*. 2017;2017:6212910. doi:10.1155/2017/6212910

176. Li YY, Chen Y, Li S, et al. Impact of Immunosuppressed Status on Prognosis of Carbapenem-Resistant Organisms Bloodstream Infections. *Infect Dis Ther*. 2024;13(4):861-874. doi:10.1007/s40121-024-00956-9

177. Li YY, Li JH, Hu T, et al. Five-year change of prevalence and risk factors for infection and mortality of carbapenem-resistant Klebsiella pneumoniae bloodstream infection in a tertiary hospital in North China. *Antimicrob Resist Infect Control*. 2020;9(1):14. doi:10.1186/s13756-020-00728-3

178. Liang X, Chen P, Deng B, et al. Outcomes and Risk Factors of Bloodstream Infections Caused by Carbapenem-Resistant and Non-Carbapenem-Resistant Klebsiella pneumoniae in China. *Infect Drug Resist*. 2022;15:3161-3171. doi:10.2147/IDR.S367588

179. Lim CL, Spelman D. Mortality impact of empirical antimicrobial therapy in ESBL- and AmpC-producing Enterobacteriaceae bacteremia in an Australian tertiary hospital. *Infect Dis Health*. 2019;24(3):124-133. doi:10.1016/j.idh.2019.02.001

180. Lin SY, Lu PL, Wu TS, et al. Correlation Between Cefoperazone/Sulbactam MIC Values and Clinical Outcomes of Escherichia coli Bacteremia. *Infect Dis Ther*. 2022;11(5):1853-1867. doi:10.1007/s40121-022-00672-2

181. Lin TC, Hung YP, Lee CC, et al. Clinical impact and risk factors of nonsusceptibility to third-generation cephalosporins among hospitalized adults with monomicrobial enterobacteriaceae bacteremia in southern Taiwan: A multicenter study. *Infect Drug Resist*. 2021;14:689-697. doi:10.2147/IDR.S297978

182. Lines J, Yang Z, Bookstaver PB, et al. Association between body mass index and mortality in patients with gram-negative bloodstream infections. *Infect Dis Clin Pract*. 2019;27(2):90-95. doi:10.1097/IPC.0000000000000711

183. Liu YD, Wang Q, Zhao CJ, et al. Prospective multi-center evaluation on risk factors, clinical characteristics and outcomes due to carbapenem resistance in Acinetobacter baumannii complex bacteraemia: experience from the Chinese Antimicrobial Resistance Surveillance of Nosocomial Infections (CARES) Network. *J Med Microbiol*. 2020;69(7):949-959. doi:10.1099/jmm.0.001222

184. Liu Y, Li F, Fang Y, et al. Clinical Characteristics, Prognosis and Treatment of Bloodstream Infections with Enterobacter Cloacae Complex in a Chinese Tertiary Hospital: A Retrospective Study. *Infect Drug Resist*. 2024;17:1811-1825. doi:10.2147/IDR.S460744

185. Lodise TP, Patel N, Kwa A, et al. Predictors of 30-day mortality among patients with Pseudomonas aeruginosa bloodstream infections:: Impact of delayed appropriate antibiotic

© 2025 La P et al. *JAMA Network Open*.

selection. *Antimicrob Agents Chemother*. 2007;51(10):3510-3515. doi:10.1128/aac.00338-07

186. Lye DC, Earnest A, Ling ML, et al. The impact of multidrug resistance in healthcare-associated and nosocomial Gram-negative bacteraemia on mortality and length of stay: cohort study. *Clin Microbiol Infect*. 2012;18(5):502-508. doi:10.1111/j.1469-0691.2011.03606.x

187. Machuca I, Gutierrez-Gutierrez B, Gracia-Ahufinger I, et al. Mortality Associated with Bacteremia Due to Colistin-Resistant Klebsiella pneumoniae with High-Level Meropenem Resistance: Importance of Combination Therapy without Colistin and Carbapenems. *Antimicrob Agents Chemother*. 2017;61(8):08. doi:10.1128/AAC.00406-17

188. Maldonado N, Lopez-Hernandez I, Lopez-Cortes LE, et al. Association of microbiological factors with mortality in Escherichia coli bacteraemia presenting with sepsis/septic shock: a prospective cohort study. *Clin Microbiol Infect*. 2024;30(8):1035-1041. doi:10.1016/j.cmi.2024.04.001

189. Man MY, Shum HP, Chan YH, et al. Clinical predictors and outcomes of Klebsiella pneumoniae bacteraemia in a regional hospital in Hong Kong. *J Hosp Infect*. 2017;97(1):35-41. doi:10.1016/j.jhin.2017.06.007

190. Man MY, Shum HP, Li KC, Yan WW. Impact of appropriate empirical antibiotics on clinical outcomes in Klebsiella pneumoniae bacteraemia. *Hong Kong Med J*. 2021;27(4):247-257. doi:10.12809/hkmj208698

191. Manesh A, Shankar C, George MM, et al. Clinical and Genomic Evolution of Carbapenem-Resistant Klebsiella pneumoniae Bloodstream Infections over Two Time Periods at a Tertiary Care Hospital in South India: A Prospective Cohort Study. *Infect Dis Ther*. 2023;12(5):1319-1335. doi:10.1007/s40121-023-00803-3

192. Marcos M, Inurrieta A, Soriano A, et al. Effect of antimicrobial therapy on mortality in 377 episodes of Enterobacter spp. bacteraemia. *J Antimicrob Chemother*. 2008;62(2):397-403. doi:10.1093/jac/dkn155

193. Martínez JA, Cobos-Trigueros N, Soriano A, et al. Influence of empiric therapy with a β-lactam alone or combined with an aminoglycoside on prognosis of bacteremia due to gram-negative microorganisms. *Antimicrob Agents Chemother*. 2010;54(9):3590-3596. doi:10.1128/AAC.00115-10

194. McCowan C, Bakhshi A, McConnachie A, et al. E. coli bacteraemia and antimicrobial resistance following antimicrobial prescribing for urinary tract infection in the community. *BMC Infect Dis*. 2022;22(1):805. doi:10.1186/s12879-022-07768-7

195. Meng H, Han L, Niu M, et al. Risk Factors for Mortality and Outcomes in Hematological Malignancy Patients with Carbapenem-Resistant Klebsiella pneumoniae Bloodstream Infections. *Infect Drug Resist*. 2022;15:4241-4251. doi:10.2147/IDR.S374904

196. Meng X, Fu J, Zheng Y, et al. Ten-Year Changes in Bloodstream Infection With Acinetobacter Baumannii Complex in Intensive Care Units in Eastern China: A Retrospective Cohort Study. *Front Med*. 2021;8:715213. doi:10.3389/fmed.2021.715213

197. Mert A, Derin O, Akalin H, et al. Multicenter evaluation of ceftazidime-avibactam use in carbapenem-resistant Klebsiella pneumoniae bloodstream infections in OXA-48 endemic regions. *Sci Rep*. 2024;14(1):26337. doi:10.1038/s41598-024-77259-z

198. Metan G, Demiraslan H, Kaynar LG, Zararsiz G, Alp E, Eser B. Factors influencing the early mortality in haematological malignancy patients with nosocomial Gram negative bacilli bacteraemia: a retrospective analysis of 154 cases. *Braz J Infect Dis*. 2013;17(2):143-149. doi:10.1016/j.bjid.2012.09.010

199. Metan G, Sariguzel F, Sumerkan B. Factors influencing survival in patients with multi-drug-resistant Acinetobacter bacteraemia. *Eur J Intern Med*. 2009;20(5):540-544. doi:10.1016/j.ejim.2009.05.005

200. Micek ST, Welch EC, Khan J, et al. Resistance to empiric antimicrobial treatment predicts outcome in severe sepsis associated with Gram-negative bacteremia. *J Hosp Med Online*. 2011;6(7):405-410. doi:10.1002/jhm.899

201. Mitsuboshi S, Tsuruma N, Watanabe K, et al. Advanced age is not a risk factor for mortality in patients with bacteremia caused by extended-spectrum β-lactamase-producing organisms: A multicenter cohort study. *Jpn J Infect Dis*. 2020;73(4):288-292. doi:10.7883/yoken.JJID.2019.411

202. Montero MM, Lopez Montesinos I, Knobel H, et al. Risk Factors for Mortality among Patients with Pseudomonas aeruginosa Bloodstream Infections: What Is the Influence of XDR Phenotype on Outcomes? *J Clin Med*. 2020;9(2):14. doi:10.3390/jcm9020514

203. Mora-Rillo M, Fernandez-Romero N, Navarro-San Francisco C, et al. Impact of virulence genes on sepsis severity and survival in Escherichia coli bacteremia. *Virulence*. 2015;6(1):93-100. doi:10.4161/21505594.2014.991234

204. Morata L, Cobos-Trigueros N, Martinez JA, et al. Influence of multidrug resistance and appropriate empirical therapy on the 30-day mortality rate of Pseudomonas aeruginosa bacteremia. *Antimicrob Agents Chemother*. 2012;56(9):4833-4837. doi:10.1128/AAC.00750-12

205. Nakanishi Y, Fukui S, Inui A, Kobayashi D, Saita M, Naito T. Predictive Rule for Mortality of Inpatients With Escherichia coli Bacteremia: Chi-Square Automatic Interaction Detector Decision Tree Analysis Model. *Cureus*. 2023;15(10):e46804. doi:10.7759/cureus.46804

206. Namikawa H, Niki M, Niki M, et al. Clinical and virulence factors related to the 30-day mortality of Klebsiella pneumoniae bacteremia at a tertiary hospital: a case-control study. *Eur J Clin Microbiol Infect Dis*. 2019;38(12):2291-2297. doi:10.1007/s10096-019-03676-y

207. Nasir N, Ahmed S, Razi S, Awan S, Mahmood SF. Risk factors for mortality of patients with ceftriaxone resistant E. coli bacteremia receiving carbapenem versus beta lactam/beta lactamase inhibitor therapy. *BMC Res Notes*. 2019;12(1):611. doi:10.1186/s13104-019-4648-7

208. Nasomsong W, Changpradub D, Vasikasin V. Impact of Inappropriate Empirical Antibiotic on Outcomes in Community-acquired Third Generation Cephalosporin Resistant

Enterobacterales Bacteremia. *Infect Chemother*. 2022;54(4):722-732. doi:10.3947/ic.2022.0096

209. Naylor NR, Pouwels KB, Hope R, et al. The health and cost burden of antibiotic resistant and susceptible Escherichia coli bacteraemia in the English hospital setting: A national retrospective cohort study. *PLoS ONE Electron Resour*. 2019;14(9):e0221944. doi:10.1371/journal.pone.0221944

210. Nelson BC, Eiras DP, Gomez-Simmonds A, et al. Clinical outcomes associated with polymyxin B dose in patients with bloodstream infections due to carbapenem-resistant Gram-negative rods. *Antimicrob Agents Chemother*. 2015;59(11):7000-7006. doi:10.1128/AAC.00844-15

211. Ngoma N, Perovic O, de Voux A, Musekiwa A, Shuping L. The impact of colistin-based regimens on mortality compared to other antimicrobials in patients with carbapenem-resistant Enterobacterales bacteremia in South African hospitals: a cross-sectional study. *BMC Infect Dis*. 2024;24(1):561. doi:10.1186/s12879-024-09459-x

212. Nham E, Huh K, Cho SY, et al. Characteristics and Clinical Outcomes of Extended-Spectrum beta-lactamase-producing Klebsiella pneumoniae Bacteremia in Cancer Patients. *Infect Chemother*. 2020;52(1):59-69. doi:10.3947/ic.2020.52.1.59

213. Ni S, Xu P, Zhang K, et al. A novel prognostic model for malignant patients with Gram-negative bacteremia based on real-world research. *Sci Rep*. 2022;12(1):11644. doi:10.1038/s41598-022-15126-5

214. Niu T, Luo Q, Li Y, Zhou Y, Yu W, Xiao Y. Comparison of Tigecycline or Cefoperazone/Sulbactam therapy for bloodstream infection due to Carbapenem-resistant Acinetobacter baumannii. *Antimicrob Resist Infect Control*. 2019;8:52. doi:10.1186/s13756-019-0502-x

215. Niu T, Xiao T, Guo L, et al. Retrospective comparative analysis of risk factors and outcomes in patients with carbapenem-resistant Acinetobacter baumannii bloodstream infections: cefoperazone-sulbactam associated with resistance and tigecycline increased the mortality. *Infect Drug Resist*. 2018;11:2021-2030. doi:10.2147/IDR.S169432

216. Niu X, Shi X, Li Q. Differences in clinical characteristics of bloodstream infections caused by escherichia coli and acinetobacter baumannii. *Int J Clin Exp Med*. 2019;12(4):4330-4338.

217. Oliva A, Volpicelli L, Di Bari S, et al. Effect of ceftazidime/avibactam plus fosfomycin combination on 30 day mortality in patients with bloodstream infections caused by KPC-producing Klebsiella pneumoniae: results from a multicentre retrospective study. *JAC-Antimicrob Resist*. 2022;4(6):dlac121. doi:10.1093/jacamr/dlac121

218. Onorato L, Sarnelli B, D’Agostino F, et al. Epidemiological, Clinical and Microbiological Characteristics of Patients with Bloodstream Infections Due to Carbapenem-Resistant K. Pneumoniae in Southern Italy: A Multicentre Study. *Antibiotics*. 2022;11(5):08. doi:10.3390/antibiotics11050633

© 2025 La P et al. *JAMA Network Open*.

219. Osih RB, McGregor JC, Rich SE, et al. Impact of empiric antibiotic therapy on outcomes in patients with Pseudomonas aeruginosa bacteremia. *Antimicrob Agents Chemother*. 2007;51(3):839-844. doi:10.1128/AAC.00901-06

220. Özdede M, Zarakolu P, Metan G, et al. Predictive modeling of mortality in carbapenem-resistant Acinetobacter baumannii bloodstream infections using machine learning. *J Investig Med*. 2024;72(7):684-696. doi:10.1177/10815589241258964

221. Palacios-Baena ZR, Delgado-Valverde M, Valiente Mendez A, et al. Impact of De-escalation on Prognosis of Patients With Bacteremia due to Enterobacteriaceae: A Post Hoc Analysis From a Multicenter Prospective Cohort. *Clin Infect Dis*. 2019;69(6):956-962. doi:10.1093/cid/ciy1032

222. Palacios-Baena ZR, Gutierrez-Gutierrez B, De Cueto M, et al. Development and validation of the INCREMENT-ESBL predictive score for mortality in patients with bloodstream infections due to extended-spectrum-beta-lactamase-producing Enterobacteriaceae. *J Antimicrob Chemother*. 2017;72(3):906-913. doi:10.1093/jac/dkw513

223. Papadimitriou-Olivgeris M, Bartzavali C, Georgakopoulou A, et al. Mortality of Pandrug-Resistant Klebsiella pneumoniae Bloodstream Infections in Critically Ill Patients: A Retrospective Cohort of 115 Episodes. *Antibiotics*. 2021;10(1):15. doi:10.3390/antibiotics10010076

224. Papadimitriou-Olivgeris M, Fligou F, Bartzavali C, et al. Carbapenemase-producing Klebsiella pneumoniae bloodstream infection in critically ill patients: risk factors and predictors of mortality. *Eur J Clin Microbiol Infect Dis*. 2017;36(7):1125-1131. doi:10.1007/s10096-017-2899-6

225. Park JH, Choi SH, Chung JW. The impact of early adequate antimicrobial therapy on 14-day mortality in patients with monomicrobial Pseudomonas aeruginosa and Acinetobacter baumannii bacteremia. *J Infect Chemother*. 2013;19(5):843-849. doi:10.1007/s10156-013-0571-3

226. Park JJ, Jung EJ, Kim JY, Seo YB, Lee J, Jung Y. Thirty-Day Mortality Rates in Patients with Extended-Spectrum beta-Lactamase-Producing Enterobacterales Bacteremia Receiving Ertapenem versus Other Carbapenems. *Antimicrob Agents Chemother*. 2022;66(7):e0028722. doi:10.1128/aac.00287-22

227. Park JW, Lee H, Park SY, Kim TH. Epidemiological, clinical, and microbiological characteristics of carbapenemase-producing Enterobacteriaceae bloodstream infection in the Republic of Korea. *Antimicrob Resist Infect Control*. 2019;8:48. doi:10.1186/s13756-019-0497-3

228. Park SH, Choi SM, Lee DG, et al. Emergence of extended-spectrum β-lactamase-producing escherichia coli as a cause of community-onset bacteremia in south korea: Risk factors and clinical outcomes. *Microb Drug Resist*. 2011;17(4):537-544. doi:10.1089/mdr.2011.0072

229. Park SY, Choo JW, Kwon SH, et al. Risk Factors for Mortality in Patients with Acinetobacter baumannii Bacteremia. *Infect Chemother*. 2013;45(3):325-330. doi:10.3947/ic.2013.45.3.325

© 2025 La P et al. *JAMA Network Open*.

230. Park SY, Lee EJ, Kim T, et al. Early administration of appropriate antimicrobial agents to improve the outcome of carbapenem-resistant Acinetobacter baumannii complex bacteraemic pneumonia. *Int J Antimicrob Agents*. 2018;51(3):407-412. doi:10.1016/j.ijantimicag.2017.10.018

231. Park SY, Park HJ, Moon SM, et al. Impact of adequate empirical combination therapy on mortality from bacteremic Pseudomonas aeruginosa pneumonia. *BMC Infect Dis*. 2012;12:308. doi:10.1186/1471-2334-12-308

232. Parkins MD, Gregson DB, Pitout JD, Ross T, Laupland KB. Population-based study of the epidemiology and the risk factors for Pseudomonas aeruginosa bloodstream infection. *Infection*. 2010;38(1):25-32. doi:10.1007/s15010-009-9145-9

233. Pascale R, Corcione S, Bussini L, et al. Non-fermentative gram-negative bloodstream infection in northern Italy: a multicenter cohort study. *BMC Infect Dis*. 2021;21(1):806. doi:10.1186/s12879-021-06496-8

234. Pedersen G, Schonheyder HC, Sorensen HT. Antibiotic therapy and outcome of monomicrobial gram-negative bacteraemia: a 3-year population-based study. *Scand J Infect Dis*. 1997;29(6):601-606. doi:10.3109/00365549709035903

235. Peña C, Cabot G, Gómez-Zorrilla S, et al. Influence of Virulence Genotype and Resistance Profile in the Mortality of Pseudomonas aeruginosa Bloodstream Infections. *Clin Infect Dis*. 2015;60(4):539-548. doi:10.1093/cid/ciu866

236. Pena C, Suarez C, Gozalo M, et al. Prospective multicenter study of the impact of carbapenem resistance on mortality in Pseudomonas aeruginosa bloodstream infections. *Antimicrob Agents Chemother*. 2012;56(3):1265-1272. doi:10.1128/AAC.05991-11

237. Peralta G, Lamelo M, Alvarez-Garcia P, et al. Impact of empirical treatment in extended-spectrum beta-lactamase-producing Escherichia coli and Klebsiella spp. bacteremia. A multicentric cohort study. *BMC Infect Dis*. 2012;12:245. doi:10.1186/1471-2334-12-245

238. Peralta G, Roiz MP, Sanchez MB, et al. Time-to-positivity in patients with Escherichia coli bacteraemia. *Clin Microbiol Infect*. 2007;13(11):1077-1082. doi:10.1111/j.1469-0691.2007.01817.x

239. Peralta G, Sanchez MB, Garrido JC, et al. Altered blood glucose concentration is associated with risk of death among patients with community-acquired Gram-negative rod bacteremia. *BMC Infect Dis*. 2010;10:181. doi:10.1186/1471-2334-10-181

240. Perez-Nadales E, Fernandez-Ruiz M, Natera AM, et al. Efficacy of ceftazidime-avibactam in solid organ transplant recipients with bloodstream infections caused by carbapenemase-producing Klebsiella pneumoniae. *Am J Transplant*. 2023;23(7):1022-1034. doi:10.1016/j.ajt.2023.03.011

241. Pérez-Nadales E, Gutiérrez-Gutiérrez B, Natera AM, et al. Predictors of mortality in solid organ transplant recipients with bloodstream infections due to carbapenemase-producing Enterobacterales: The impact of cytomegalovirus disease and lymphopenia. *Am J Transplant*. 2020;20(6):1629-1641. doi:10.1111/ajt.15769

© 2025 La P et al. *JAMA Network Open*.

242. Persoon MC, Voor In't Holt AF, Wielders CCH, Gommers D, Vos MC, Severin JA. Mortality associated with carbapenem-susceptible and Verona Integron-encoded Metallo-beta-lactamase-positive Pseudomonas aeruginosa bacteremia. *Antimicrob Resist Infect Control*. 2020;9(1):25. doi:10.1186/s13756-020-0682-4

243. Phe K, Bowers DR, Babic JT, Tam VH. Outcomes of empiric aminoglycoside monotherapy for Pseudomonas aeruginosa bacteremia. *Diagn Microbiol Infect Dis*. 2019;93(4):346-348. doi:10.1016/j.diagmicrobio.2018.10.019

244. Phungoen P, Sarunyaparit J, Apiratwarakul K, Wonglakorn L, Meesing A, Sawanyawisuth K. The association of ESBL Escherichia coli with mortality in patients with Escherichia coli bacteremia at the emergency department. *Drug Target Insights*. 2022;16(1):12-16. doi:10.33393/dti.2022.2422

245. Poon LM, Jin J, Chee YL, et al. Risk factors for adverse outcomes and multidrug-resistant Gram-negative bacteraemia in haematology patients with febrile neutropenia in a Singaporean university hospital. *Singapore Med J*. 2012;53(11):720-725.

246. Qian C, Wu Q, Ruan Z, et al. A Visualized Mortality Prediction Score Model in Hematological Malignancies Patients with Carbapenem-Resistant Organisms Bloodstream Infection. *Infect Drug Resist*. 2023;16:201-215. doi:10.2147/IDR.S393932

247. Quillici MCB, Resende DS, Goncalves IR, et al. Gram-negative bacilli bacteremia: a 7 year retrospective study in a referral Brazilian tertiary-care teaching hospital. *J Med Microbiol*. 2021;70(1). doi:10.1099/jmm.0.001277

248. Qureshi ZA, Paterson DL, Peleg AY, et al. Clinical characteristics of bacteraemia caused by extended-spectrum beta-lactamase-producing Enterobacteriaceae in the era of CTX-M-type and KPC-type beta-lactamases. *Clin Microbiol Infect*. 2012;18(9):887-893. doi:10.1111/j.1469-0691.2011.03658.x

249. Recio R, Mancheno M, Viedma E, et al. Predictors of Mortality in Bloodstream Infections Caused by Pseudomonas aeruginosa and Impact of Antimicrobial Resistance and Bacterial Virulence. *Antimicrob Agents Chemother*. 2020;64(2):27. doi:10.1128/AAC.01759-19

250. Reese M, Bookstaver PB, Kohn J, et al. Missed Opportunities for Early De-Escalation of Antipseudomonal Beta-Lactam Antimicrobial Therapy in Enterobacterales Bloodstream Infection. *Antibiotics*. 2024;13(11):31. doi:10.3390/antibiotics13111031

251. Rhodes NJ, Kuti JL, Nicolau DP, et al. Defining Clinical Exposures of Cefepime for Gram-Negative Bloodstream Infections That Are Associated with Improved Survival. *Antimicrob Agents Chemother*. 2015;60(3):1401-1410. doi:10.1128/AAC.01956-15

252. Rigatto MH, Ramos F, Barros A, et al. Double-, single- and none-carbapenem-containing regimens for the treatment of carbapenem-resistant Enterobacterales (CRE) bloodstream infections: a retrospective cohort. *J Antimicrob Chemother*. 2022;77(11):3118-3125. doi:10.1093/jac/dkac292

253. Rodríguez-Baño J, Navarro MD, Retamar P, Picón E, Pascual A. β-Lactam/β-lactam inhibitor combinations for the treatment of bacteremia due to extended-spectrum β-

lactamase-producing Escherichia coli: A post hoc analysis of prospective cohorts. *Clin Infect Dis*. 2012;54(2):167-174. doi:10.1093/cid/cir790

254. Rodriguez-Bano J, Picon E, Gijon P, et al. Community-onset bacteremia due to extended-spectrum beta-lactamase-producing Escherichia coli: risk factors and prognosis. *Clin Infect Dis*. 2010;50(1):40-48. doi:10.1086/649537

255. Rolo M, Martín-Higuera MC, Viedma E, et al. Clinical impact of time-to-positivity of blood cultures on mortality in patients with Pseudomonas aeruginosa bacteremia. *J Glob Antimicrob Resist*. 2022;30:269-275. doi:10.1016/j.jgar.2022.06.026

256. Russo A, Bassetti M, Ceccarelli G, et al. Bloodstream infections caused by carbapenem-resistant Acinetobacter baumannii: Clinical features, therapy and outcome from a multicenter study. *J Infect*. 2019;79(2):130-138. doi:10.1016/j.jinf.2019.05.017

257. Sangsuwan T, Komet O, Laochareonsuk W, Jamulitrat S. Comparison of virulence attributable to different levels of antimicrobial resistant acinetobacter baumannii bacteremia. *J Med Assoc Thai*. 2021;104(5):715-722. doi:10.35755/jmedassocthai.2021.05.10894

258. Sathya Kumar AM, George MM, Bhanuprasad K, et al. Persistent bacteremia predicts poor outcomes among neutropenic patients with carbapenem-resistant gram-negative bloodstream infections receiving appropriate therapy. *Ann Clin Microbiol Antimicrob*. 2023;22(1). doi:10.1186/s12941-023-00561-7

259. Scheich S, Weber S, Reinheimer C, et al. Bloodstream infections with gram-negative organisms and the impact of multidrug resistance in patients with hematological malignancies. *Ann Hematol*. 2018;97(11):2225-2234. doi:10.1007/s00277-018-3423-5

260. Scheuerman O, Schechner V, Carmeli Y, et al. Comparison of Predictors and Mortality Between Bloodstream Infections Caused by ESBL-Producing Escherichia coli and ESBL-Producing Klebsiella pneumoniae. *Infect Control Hosp Epidemiol*. 2018;39(6):660-667. doi:10.1017/ice.2018.63

261. Schwaber MJ, Navon-Venezia S, Kaye KS, Ben-Ami R, Schwartz D, Carmeli Y. Clinical and economic impact of bacteremia with extended-spectrum-β- lactamase-producing Enterobacteriaceae. *Antimicrob Agents Chemother*. 2006;50(4):1257-1262. doi:10.1128/AAC.50.4.1257-1262.2006

262. Seo H, Lee SC, Chung H, et al. Clinical and Microbiological Analysis of Risk Factors for Mortality in Patients with Carbapenem-Resistant Enterobacteriaceae Bacteremia. *Int J Antimicrob Agents*. 2020;56(4):106126. doi:10.1016/j.ijantimicag.2020.106126

263. Shalabi O, Kashat L, Murik O, Zevin S, Assous MV, Ben-Chetrit E. Clinical Outcomes of Patients with AmpC-Beta-Lactamase-Producing Enterobacterales Bacteremia Treated with Carbapenems versus Non-Carbapenem Regimens: A Single-Center Study. *Antibiotics*. 2024;13(8):29. doi:10.3390/antibiotics13080709

264. Shi N, Kang J, Wang S, et al. Bacteriological Profile and Antimicrobial Susceptibility Patterns of Gram-Negative Bloodstream Infection and Risk Factors Associated with

Mortality and Drug Resistance: A Retrospective Study from Shanxi, China. *Infect Drug Resist*. 2022;15:3561-3578. doi:10.2147/IDR.S370326

265. Shi Q, Huang C, Xiao T, Wu Z, Xiao Y. A retrospective analysis of Pseudomonas aeruginosa bloodstream infections: prevalence, risk factors, and outcome in carbapenem-susceptible and -non-susceptible infections. *Antimicrob Resist Infect Control*. 2019;8:68. doi:10.1186/s13756-019-0520-8

266. Siedner MJ, Galar A, Guzman-Suarez BB, et al. Cefepime vs other antibacterial agents for the treatment of Enterobacter species bacteremia. *Clin Infect Dis*. 2014;58(11):1554-1563. doi:10.1093/cid/ciu182

267. Simsek Bozok T, Bozok T, Sahinoglu MS, Kaya H, Horasan ES, Kaya A. Bloodstream infections caused by carbapenem-resistant Klebsiella pneumoniae: analysis of risk factors, treatment responses and mortality. *Infect Dis*. Published online 2024:1-11. doi:10.1080/23744235.2024.2436991

268. Son HJ, Cho EB, Bae M, et al. Clinical and Microbiological Analysis of Risk Factors for Mortality in Patients With Carbapenem-Resistant Acinetobacter baumannii Bacteremia. *Open Forum Infect Dis*. 2020;7(10):ofaa378. doi:10.1093/ofid/ofaa378

269. Suh JW, Park SM, Ju YK, et al. Clinical and molecular predictors of mortality in patients with carbapenem-resistant Acinetobacter baumannii bacteremia: A retrospective cohort study. *J Microbiol Immunol Infect*. 2024;57(1):148-155. doi:10.1016/j.jmii.2023.11.001

270. Swingler EA, Clark M, Moore SE, et al. Effect of a pharmacy-driven rapid bacteremia response program on outcomes in adult patients with extended-spectrum beta-lactamase bacteremia: A retrospective, quasi-experimental study. *J Am Coll Clin Pharm*. Published online 2024:8. doi:10.1002/jac5.2063

271. Szilagyi E, Fuzi M, Borocz K, Kurcz A, Toth A, Nagy K. Risk factors and outcomes for bloodstream infections with extended-spectrum beta -lactamase-producing Klebsiella pneumoniae ; Findings of the nosocomial surveillance system in Hungary. *Acta Microbiol Immunol Hung*. 2009;56(3):251-262. doi:10.1556/AMicr.56.2009.3.5

272. Tam VH, Rogers CA, Chang KT, Weston JS, Caeiro JP, Garey KW. Impact of multidrug-resistant Pseudomonas aeruginosa bacteremia on patient outcomes. *Antimicrob Agents Chemother*. 2010;54(9):3717-3722. doi:10.1128/AAC.00207-10

273. Tang PC, Lee CC, Li CW, Li MC, Ko WC, Lee NY. Time-to-positivity of blood culture: An independent prognostic factor of monomicrobial Pseudomonas aeruginosa bacteremia. *J Microbiol Immunol Infect*. 2017;50(4):486-493. doi:10.1016/j.jmii.2015.08.014

274. Tang Y, Xu C, Xiao H, Wang L, Cheng Q, Li X. Gram-Negative Bacteria Bloodstream Infections in Patients with Hematological Malignancies - The Impact of Pathogen Type and Patterns of Antibiotic Resistance: A Retrospective Cohort Study. *Infect Drug Resist*. 2021;14:3115-3124. doi:10.2147/IDR.S322812

275. Tinevez C, Velardo F, Ranc AG, et al. Retrospective Multicentric Study on Campylobacter spp. Bacteremia in France: The Campylobacteremia Study. *Clin Infect Dis*. 2022;75(4):702-709. doi:10.1093/cid/ciab983

276. Tiseo G, Galfo V, Carbonara S, et al. Bacteremic nosocomial pneumonia caused by Gram-negative bacilli: results from the nationwide ALARICO study in Italy. *Infection*. 2024;06:06. doi:10.1007/s15010-024-02423-6

277. Trecarichi EM, Giuliano G, Cattaneo C, et al. Bloodstream infections caused by Escherichia coli in onco-haematological patients: Risk factors and mortality in an Italian prospective survey. *PLoS ONE Electron Resour*. 2019;14(10):e0224465. doi:10.1371/journal.pone.0224465

278. Trecarichi EM, Pagano L, Martino B, et al. Bloodstream infections caused by Klebsiella pneumoniae in onco-hematological patients: clinical impact of carbapenem resistance in a multicentre prospective survey. *Am J Hematol*. 2016;91(11):1076-1081. doi:10.1002/ajh.24489

279. Tu B, Bi J, Wu D, et al. Bloodstream infection due to Escherichia coli in liver cirrhosis patients: Clinical features and outcomes. *Oncotarget*. 2018;9(87):35780-35789.

280. Tumbarello M, Repetto E, Trecarichi EM, et al. Multidrug-resistant Pseudomonas aeruginosa bloodstream infections: risk factors and mortality. *Epidemiol Infect*. 2011;139(11):1740-1749. doi:10.1017/S0950268810003055

281. Tumbarello M, Sali M, Trecarichi EM, et al. Bloodstream infections caused by extended-spectrum-β-lactamase- producing Escherichia coli: Risk factors for inadequate initial antimicrobial therapy. *Antimicrob Agents Chemother*. 2008;52(9):3244-3252. doi:10.1128/AAC.00063-08

282. Tumbarello M, Sanguinetti M, Montuori E, et al. Predictors of mortality in patients with bloodstream infections caused by extended-spectrum-beta-lactamase-producing Enterobacteriaceae: importance of inadequate initial antimicrobial treatment. *Antimicrob Agents Chemother*. 2007;51(6):1987-1994. doi:10.1128/AAC.01509-06

283. Tumbarello M, Viale P, Viscoli C, et al. Predictors of mortality in bloodstream infections caused by Klebsiella pneumoniae carbapenemase-producing K. pneumoniae: importance of combination therapy. *Clin Infect Dis*. 2012;55(7):943-950. doi:10.1093/cid/cis588

284. Vieceli T, Henrique LR, Rech TH, Zavascki AP. Colistin versus polymyxin B for the treatment of carbapenem-resistant Klebsiella pneumoniae bloodstream infections. *J Infect Chemother*. 2024;30(7):621-625. doi:10.1016/j.jiac.2024.01.012

285. Wang CP, Hsieh MS, Hu SY, Huang SC, Tsai CA, Shen CH. Risk Factors and Scoring Systems to Predict the Mortality Risk of Afebrile Adult Patients with Monomicrobial Gram-Negative Bacteremia: A 10-Year Observational Study in the Emergency Department. *Diagnostics*. 2024;14(9):23. doi:10.3390/diagnostics14090869

286. Wang JL, Lee CC, Lee CH, et al. Clinical impact of sequence type 131 in adults with community-onset monomicrobial Escherichia coli Bacteremia. *J Clin Med*. 2018;7(12). doi:10.3390/jcm7120508

287. Wang J, Mu M, Zhu J, et al. Adult acute leukemia patients with gram-negative bacteria bloodstream infection: Risk factors and outcomes of antibiotic-resistant bacteria. *Ann Hematol*. 2024;103(10):4021-4031. doi:10.1007/s00277-024-05866-x

© 2025 La P et al. *JAMA Network Open*.

288. Wang J, Zhang J, Wu ZH, et al. Clinical Characteristics and Prognosis Analysis of Acinetobacter baumannii Bloodstream Infection Based on Propensity Matching. *Infect Drug Resist*. 2022;15:6963-6974. doi:10.2147/IDR.S387898

289. Wang L, Zeng C, Li X, Li Y, Liu Z, Hu J. Mortality associated with carbapenem resistance in Klebsiella pneumoniae bloodstream infection: A propensity score-matched study. *Infect Control Hosp Epidemiol*. 2024;45(7):839-846. doi:10.1017/ice.2024.21

290. Wang X, Wang Q, Cao B, et al. Retrospective Observational Study from a Chinese Network of the Impact of Combination Therapy versus Monotherapy on Mortality from Carbapenem-Resistant Enterobacteriaceae Bacteremia. *Antimicrob Agents Chemother*. 2019;63(1):01. doi:10.1128/AAC.01511-18

291. Watanakunakorn C, Perni SC. Proteus mirabilis bacteremia: a review of 176 cases during 1980-1992. *Scand J Infect Dis*. 1994;26(4):361-367. doi:10.3109/00365549409008605

292. Wei ZY, Zhao LN, Yan J, et al. Dynamic monitoring of neutrophil/lymphocyte ratio, APACHE II score, and SOFA score predict prognosis and drug resistance in patients with Acinetobacter baumannii-calcoaceticus complex bloodstream infection: a single-center retrospective study. *Front Microbiol*. 2024;15:13. doi:10.3389/fmicb.2024.1296059

293. Wen CY, Hu SY, Hsieh MS, Huang SC, Shen CH, Tsai YC. Good Performance of Revised Scoring Systems in Predicting Clinical Outcomes of Aeromonas Bacteremia in the Emergency Department: A Retrospective Observational Study. *Diagnostics*. 2024;14(2):05. doi:10.3390/diagnostics14020124

294. Willmann M, Kuebart I, Marschal M, et al. Effect of metallo-β-lactamase production and multidrug resistance on clinical outcomes in patients with Pseudomonas aeruginosa bloodstream infection: A retrospective cohort study. *BMC Infect Dis*. 2013;13(1). doi:10.1186/1471-2334-13-515

295. Wu H, Mao YH, Du XX, Zhao F, Jiang Y, Yu YS. The Value of Neutrophil-To-Lymphocyte Ratio for Evaluating Blood Stream Infection Caused by Carbapenem-Resistant Klebsiella pneumoniae: A Retrospective Cohort Study. *Front Med*. 2022;9:10. doi:10.3389/fmed.2022.832655

296. Wu HS, Wang FD, Tseng CP, Wu TH, Lin YT, Fung CP. Characteristics of healthcare-associated and community-acquired Klebsiella pneumoniae bacteremia in Taiwan. *J Infect*. 2012;64(2):162-168. doi:10.1016/j.jinf.2011.11.005

297. Wu PF, Lin YT, Wang FD, Yang TC, Fung CP. Is fluoroquinolone monotherapy a useful alternative treatment for Pseudomonas aeruginosa bacteraemia? *Infection*. 2018;46(3):365-373. doi:10.1007/s15010-018-1131-7

298. Xiao T, Yang K, Zhou Y, et al. Risk factors and outcomes in non-transplant patients with extended-spectrum beta-lactamase-producing Escherichia coli bacteremia: a retrospective study from 2013 to 2016. *Antimicrob Resist Infect Control*. 2019;8:144. doi:10.1186/s13756-019-0599-y

299. Xiao T, Yu W, Niu T, Huang C, Xiao Y. A retrospective, comparative analysis of risk factors and outcomes in carbapenem-susceptible and carbapenem-nonsusceptible Klebsiella

pneumoniae bloodstream infections: tigecycline significantly increases the mortality. *Infect Drug Resist*. 2018;11:595-606. doi:10.2147/IDR.S153246

300. Xiao T, Zhu Y, Zhang S, et al. A Retrospective Analysis of Risk Factors and Outcomes of Carbapenem-Resistant Klebsiella pneumoniae Bacteremia in Nontransplant Patients. *J Infect Dis*. 2020;221(Suppl 2):S174-S183. doi:10.1093/infdis/jiz559

301. Xu J, Chen XJ, Zheng X. Acinetobacter baumannii complex-caused bloodstream infection in ICU during a 12-year period: Predicting fulminant sepsis by interpretable machine learning. *Front Microbiol*. 2022;13:9. doi:10.3389/fmicb.2022.1037735

302. Xu M, Fu Y, Kong H, et al. Bloodstream infections caused by Klebsiella pneumoniae: Prevalence of bla KPC, virulence factors and their impacts on clinical outcome. *BMC Infect Dis*. 2018;18(1). doi:10.1186/s12879-018-3263-x

303. Xu P, Zhang X, Chen Q, et al. Clinical features and risk factors for mortality in patients with Klebsiella pneumoniae bloodstream infections. *J Infect Dev Ctries*. 2024;18(6):843-850. doi:10.3855/jidc.18649

304. Xu Q, Zheng B, Shen P, Xiao Y. Protective efficacy of statins in patients with Klebsiella pneumoniae bloodstream infection. *Front Cell Infect Microbiol*. 2022;12:1087701. doi:10.3389/fcimb.2022.1087701

305. Xu S, Song Z, Han F, Zhang C. Effect of appropriate empirical antimicrobial therapy on mortality of patients with Gram-negative bloodstream infections: a retrospective cohort study. *BMC Infect Dis*. 2023;23(1):344. doi:10.1186/s12879-023-08329-2

306. Yang JL, Yang CJ, Chuang YC, Sheng WH, Chen YC, Chang SC. Association of capsular polysaccharide locus 2 with prognosis of Acinetobacter baumannii bacteraemia. *Emerg Microbes Infect*. 2022;11(1):83-90. doi:10.1080/22221751.2021.2011624

307. Yang S, Sun J, Wu X, Zhang L. Determinants of Mortality in Patients with Nosocomial Acinetobacter baumannii Bacteremia in Southwest China: A Five-Year Case-Control Study. *Can J Infect Dis Med Microbiol*. 2018;2018:3150965. doi:10.1155/2018/3150965

308. Yang X, Man MY, Heng H, et al. Molecular epidemiology and clinical impact of Klebsiella spp. causing bloodstream infections in Hong Kong. *EBioMedicine*. 2024;101:104998. doi:10.1016/j.ebiom.2024.104998

309. Yang YY, Tsai IT, Lai CH, Chen CP, Chen CC, Hsu YC. Time to positivity of Klebsiella pneumoniae in blood cultures as prognostic marker in patients with intra-abdominal infection: A retrospective study. *Virulence*. 2024;15(1):2329397. doi:10.1080/21505594.2024.2329397

310. Yildiz M, Habibi H, Altin FB, Corbacioglu SK, Ozger HS. The effect of follow-up blood cultures on mortality and antibiotic use in gram-negative bloodstream infections. *BMC Infect Dis*. 2023;23(1):564. doi:10.1186/s12879-023-08500-9

311. Yoon EJ, Kim D, Lee H, et al. Mortality dynamics of Pseudomonas aeruginosa bloodstream infections and the influence of defective OprD on mortality: prospective

observational study. *J Antimicrob Chemother*. 2019;74(9):2774-2783. doi:10.1093/jac/dkz245

312. Yoon YK, Kim HA, Ryu SY, et al. Tree-structured survival analysis of patients with Pseudomonas aeruginosa bacteremia: A multicenter observational cohort study. *Diagn Microbiol Infect Dis*. 2017;87(2):180-187. doi:10.1016/j.diagmicrobio.2016.10.008

313. You TY, Lo CL, Tsai WC, Jan HE, Ko WC, Lee NY. Efficacy of short- versus prolonged-courses of antimicrobial therapy for carbapenem-resistant Klebsiella pneumoniae bloodstream infections: A propensity score-matched cohort study. *J Microbiol Immunol Infect*. 2024;57(4):594-600. doi:10.1016/j.jmii.2024.05.010

314. Yu H, Hu R, Hu X, Lu Y, Yao Y, Su J. Risk factors for bacteremia and mortality due to multidrug-resistant Acinetobacter baumannii: a retrospective study. *Lett Appl Microbiol*. 2024;77(2):01. doi:10.1093/lambio/ovae006

315. Yuan F, Li M, Wang X, Fu Y. Risk factors and mortality of carbapenem-resistant Pseudomonas aeruginosa bloodstream infection in haematology department: A 10-year retrospective study. *J Glob Antimicrob Resist*. 2024;37:150-156. doi:10.1016/j.jgar.2024.03.018

316. Yuan Q, Guo L, Li B, et al. Risk factors and outcomes of inpatients with carbapenem-resistant Pseudomonas aeruginosa bloodstream infections in China: a 9-year trend and multicenter cohort study. *Front Microbiol*. 2023;14:1137811. doi:10.3389/fmicb.2023.1137811

317. Zhang G, Zhang M, Sun F, et al. Epidemiology, mortality and risk factors for patients with K. pneumoniae bloodstream infections: Clinical impact of carbapenem resistance in a tertiary university teaching hospital of Beijing. *J Infect Public Health*. 2020;13(11):1710-1714. doi:10.1016/j.jiph.2020.09.012

318. Zhang Q, Gao HY, Li D, et al. Clinical outcome of Escherichia coli bloodstream infection in cancer patients with/without biofilm formation: a single-center retrospective study. *Infect Drug Resist*. 2019;12:359-371. doi:10.2147/IDR.S192072

319. Zhang Q, Wang P, Shen M, Shan C. Clinical characteristics and risk factors for Klebsiella pneumoniae bloodstream infection in 152 immunocompetent patients. *J Infect Dev Ctries*. 2024;18(2):219-226. doi:10.3855/jidc.18204

320. Zhang Q, Zhang W, Li Z, et al. Bacteraemia due to AmpC beta-lactamase-producing Escherichia coli in hospitalized cancer patients: risk factors, antibiotic therapy, and outcomes. *Diagn Microbiol Infect Dis*. 2017;88(3):247-251. doi:10.1016/j.diagmicrobio.2017.04.006

321. Zhang S, Yang Z, Sun L, et al. Clinical Observation and Prognostic Analysis of Patients With Klebsiella pneumoniae Bloodstream Infection. *Front Cell Infect Microbiol*. 2020;10:577244. doi:10.3389/fcimb.2020.577244

322. Zhao C, Zheng Y, Hang Y, et al. Risk Factors for 30-Day Mortality in Patients with Bacteremic Pneumonia Caused by Escherichia coli and Klebsiella pneumoniae: A Retrospective Study. *Int J Gen Med*. 2023;16:6163-6176. doi:10.2147/IJGM.S447354

323. Zhao S, Wu Y, Dai Z, Chen Y, Zhou X, Zhao J. Risk factors for antibiotic resistance and mortality in patients with bloodstream infection of Escherichia coli. *Eur J Clin Microbiol Infect Dis*. 2022;41(5):713-721. doi:10.1007/s10096-022-04423-6

324. Zhao Y, Lin Q, Liu L, et al. Risk Factors and Outcomes of Antibiotic-resistant Pseudomonas aeruginosa Bloodstream Infection in Adult Patients With Acute Leukemia. *Clin Infect Dis*. 2020;71(Suppl 4):S386-S393. doi:10.1093/cid/ciaa1522

325. Zhou C, Jin L, Wang Q, et al. Bloodstream Infections Caused by Carbapenem-Resistant Enterobacterales: Risk Factors for Mortality, Antimicrobial Therapy and Treatment Outcomes from a Prospective Multicenter Study. *Infect Drug Resist*. 2021;14:731-742. doi:10.2147/IDR.S294282

326. Zhou H, Yao Y, Zhu B, et al. Risk factors for acquisition and mortality of multidrug-resistant Acinetobacter baumannii bacteremia: A retrospective study from a Chinese hospital. *Medicine (Baltimore)*. 2019;98(13):e14937. doi:10.1097/MD.0000000000014937

327. Zhou J, Sun J, Lu S, et al. Clinical characteristics and prognosis of bloodstream infections with carbapenem-resistant Gram-negative organisms in patients with hematological malignancies: A multicenter case-control study in China. *J Infect*. 2024;89(6):106331. doi:10.1016/j.jinf.2024.106331

328. Zou XL, Feng DY, Wu WB, Yang HL, Zhang TT. Blood urea nitrogen to serum albumin ratio independently predicts 30-day mortality and severity in patients with Escherichia coli bacteraemia. *Med Clínica*. 2021;157(5):219-225. doi:10.1016/j.medcli.2020.06.060

© 2025 La P et al. *JAMA Network Open*.
