# Supplementary material for: Female Sex and Mortality in Patients With Gram-Negative Bacteremia: A Systematic Review and Meta-Analysis
Source: JAMA Netw Open. 2025 Nov 13;8(11):e2543552. doi: 10.1001/jamanetworkopen.2025.43552 (PMC12616467; doi:10.1001/jamanetworkopen.2025.43552)
Supplement: Supplement 2. — Data Sharing Statement [file jamanetwopen-e2543552-s002.pdf]

Data Sharing Statement

La. Female Sex and Mortality in Patients With Gram-Negative Bacteremia. *JAMA Netw Open*. Published November 13, 2025. doi:10.1001/jamanetworkopen.2025.43552

Data
Data available: No

Additional Information
Explanation for why data not available: We do not have patient level data in this study as it is a systematic review and meta-analysis.
